# Synergistic mastery: Advancing mechanical and electrical harmony in conducting polymer hydrogel bioelectronics

**DOI:** 10.1016/j.bioactmat.2025.06.015

**Published:** 2025-06-11

**Authors:** Ting Wang, Jiajun Liu, Yuli Zhao, Yuan Lu

**Affiliations:** a Department of Chemical Engineering, Tsinghua University, Beijing, 100084, China; b State Key Laboratory of Green Biomanufacturing, Department of Chemical Engineering, Tsinghua University, Beijing, 100084, China; c Key Laboratory of Industrial Biocatalysis, Ministry of Education, Department of Chemical Engineering, Tsinghua University, Beijing, 100084, China; d Ordos Laboratory, Ordos, Inner Mongolia, 017000, China

**Keywords:** Conducting polymer hydrogel, Mechanical properties, Electrical properties, Epidermal or implantable bioelectrodes

## Abstract

Conducting polymer hydrogels offer promising electrical interfaces with biological tissues for electrophysiological signal recording, sensing, and stimulation due to their favorable electrical properties, biocompatibility, and stability. Among them, Poly (3,4-ethylenedioxythiophene): Polystyrene sulfonate (PEDOT:PSS) is widely used as a conductive filler, forming a network of conjugated nanofibers within the hydrogel matrix. This structure enables robust electronic conductivity while preserving ionic transport and biocompatibility in physiological environments. However, the mechanical integrity of these hydrogels is often compromised by micellar microstructures in aqueous colloidal dispersions. The absence of interconnected conducting polymer nanofibers to maintain mechanical integrity during swelling hinders the mechanical properties of hydrogels. Here, three modification strategies were explored to enhance the flexibility and stretchability: constructing an interpenetrating network, phase separation induced by ionic compounds, and pure conductive hydrogels formed through polar solvent additives and dry-annealing. These strategies synergistically enhance conductivity and flexibility by controlling interchain entanglement and redesigning the distribution of conjugated crystal regions and soft regions. The resulting hydrogels exhibit excellent conductivity (1.99–5.25 S/m), softness (elastic modulus as low as 280 Pa), and elasticity (tensile properties up to 800 %). When used as epidermal or implantable bioelectrodes, they provided a soft interface, ensuring longer-lasting and more stable electromyogram, electrocardiogram, and electroencephalogram signals compared to commercial gel electrodes, with a signal-to-noise ratio of up to 20.0 dB. Therefore, the conducting polymer hydrogels developed in this study leverage the synergy between conductivity and flexibility, paving the way for further transformative applications in bioelectronics.

## Highlights

• Multi-strategy framework enables full-spectrum modulus (0.28–15 kPa) with high conductivity (1.99–5.25 S/m).
• Multi-criteria screening optimizes mechanics, conductivity, and biocompatibility.
• Hydrogels retain >79 % stress after 1,000 mechanical cycles and >87 % charge capacity after 100,000 electrochemical cycles.
• Demonstrated stable epidermal or implantable EMG/ECG/EEG recordings with SNR up to 20.0 dB.
• Through coordinated optimization, systematic screening, and application verification, the paradigm guides rational design of bioelectronics.

## Graphical abstract

Three modification strategies were explored to enhance the flexibility and stretchability: the construction of an interpenetrating network, phase separation induced by ionic compounds, and pure conductive hydrogels formed through polar solvent additives and dry-annealing. The resulting hydrogels exhibit excellent conductivity, softness, and elasticity for use as epidermal or implantable bioelectrodes.

**Figure:**
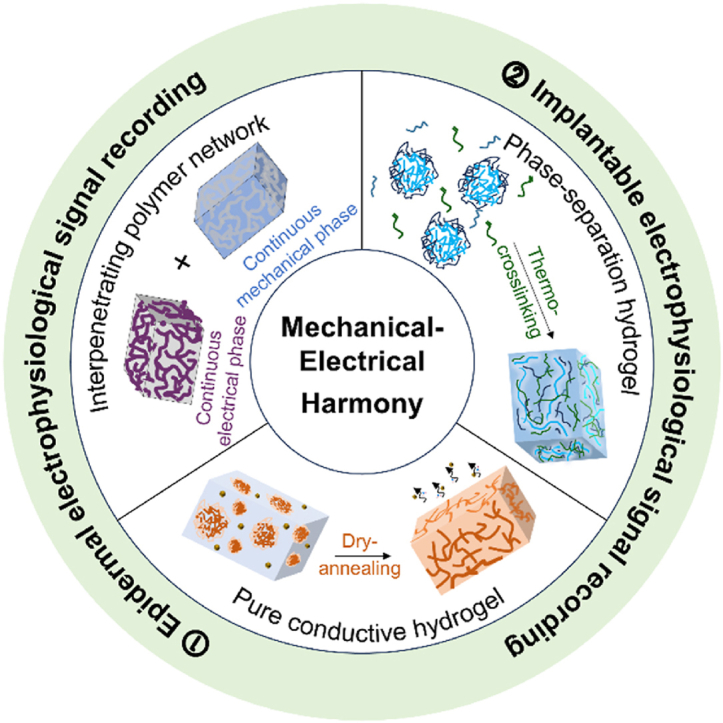

## 1 Introduction

Advanced bioelectronics hold revolutionary potential in biomedical applications, offering exceptional diagnostic and therapeutic capabilities such as enabling electrical stimuli to bio-tissues and accurately recording biological signals. Unfortunately, traditional bioelectronics are made of rigid materials with large mechanical mismatch with organs, resulting in a non-conformable electronics-tissue interface [1]. Previous studies have demonstrated that minimizing stiffness mismatch reduces damage to underlying tissues [2]. A number of bioelectronics have emerged recently, including commercially available silicon probes, epidermal electrodes, and nerve interfaces, narrowing the gap between electronic systems and the human body [[3], [4], [5]]. However, most approaches have primarily focused on the structural design of the electronics, such as employing ultrathin electronics and macroporous mesh structures to decrease the bending stiffness [6,7]. Hence, exploring intrinsically stretchable electronic materials with a low elastic modulus could present an alternative approach to achieving the versatility of tissue-like bioelectronics.

Conductive hydrogels stand out as promising electronic interfacing materials for biological tissues, owing to their tissue-mimicking mechanical properties. Biological tissues typically exhibit softness with low elastic moduli (1 kPa–1 MPa) and contain significant amounts of water, often exceeding 70 % [8]. In contrast, most inorganic materials and dry polymers utilized in bioelectronic devices, such as metal and polycarbonate, exhibit much higher elastic moduli (100 MPa–10 GPa) with virtually no water content [9]. Among engineering materials, conductive hydrogels are the ideal alternatives due to their unique combination of water-rich composition, superior biocompatibility, intrinsic mechanical compliance, and electrical conductivity [[10], [11], [12], [13]].

Conducting polymer hydrogels uniquely combine high electrical conductivity, stability in physiological environments, and excellent biocompatibility—advantages not shared by systems based solely on ionic salts, metals, or carbon nanomaterials [[14], [15], [16], [17]]. Conducting polymers rely on π-conjugated backbones to form continuous electronic pathways [18,19], yet their rigid aromatic structures render them hydrophobic and brittle, with fracture strains typically below 2 % [20,21]. Among them, PEDOT:PSS stands out for its dual electronic and ionic conductivity and has been integrated into wearable electronic fabric, bioelectronic interfaces, soft robotics, electrochromic devices, and flexible solar cells [[22], [23], [24], [25], [26]]. Nevertheless, PEDOT:PSS films remain intrinsically brittle, and even chemically modified hydrogels seldom exceed 200 % elongation. Their elastic moduli also far exceed that of soft tissues such as the brain (<1 kPa) [22,23], leaving a critical gap between electrical performance and the mechanical compliance required for truly flexible bioelectronics.

Various methods have been designed to engineer conducting polymer hydrogels. These methods typically include direct gelation of the mixture of conducting polymers and hydrophilic polymers/monomers through the self-assembly [27,28], in-situ polymerization of conducting polymers within a preformed hydrogel matrix [29,30], and self-crosslinked conducting polymer hydrogels that avoid the use of insulating components [31,32]. While these strategies have improved conductivity and processability, they have not overcome the fundamental trade-off: tensile strains remain under 200 % [33,34], and moduli stay well above the sub-kilopascal range needed for seamless tissue interfaces [35,36]. Addressing this challenge demands new design paradigms that simultaneously deliver ultra-low modulus, high conductivity, and long-term mechanical and electrochemical stability.

**Fig. 1:**
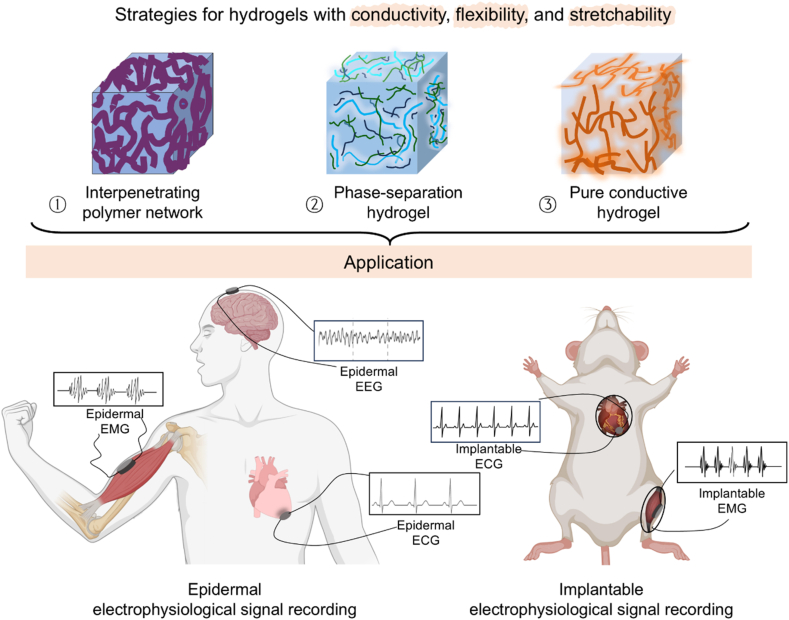
Three modification strategies were explored to enhance the flexibility and stretchability of conducting polymer hydrogel using as epidermal or implantable bioelectrodes: the construction of an interpenetrating network, phase separation induced by ionic compounds, and pure conductive hydrogels formed through polar solvent additives and dry-annealing.

Herein, to systematically address the conductivity-flexibility trade-off, we propose three complementary strategies targeting distinct yet synergistic aspects of microstructure engineering. These strategies involved: (1) Utilizing conducting polymers as conductive dopants within non-conductive hydrogel templates to form interpenetrating network conductive hydrogels (IPNCHs), (2) Blending ionic compounds into conducting polymers to promote polymer aggregation to form phase separation conductive hydrogels (PSCHs), and (3) Introducing polar solvent additives into conducting polymers followed by controlled dry-annealing to yield pure conductive hydrogels (PCHs). Together, these strategies span the broad mechanical-electrical spectrum required for diverse bioelectronic applications. By synergistically balancing mechanical compliance and electrical conductivity, the resulting hydrogels function effectively as bioelectrodes for both epidermal and implantable electrophysiological signal recording, delivering stable, reliable signals with high signal-to-noise ratios (Fig. 1). The modular design approach developed in this work addresses a critical gap in the current literature, where simultaneous optimization of multiple key properties has often been overlooked. This framework not only advances the fundamental understanding of structure–property relationships in conducting polymer hydrogels, but also offers valuable insights for the development of highly flexible, stretchable materials in bioelectronics.

## 2 Experimental section

### 2.1 Materials

PEDOT:PSS aqueous solution (solid content 1.1–1.3 %, Clevios PH1000) was purchased from Heraeus electronic materials. κ-Carrageenan (CA, CAS #11114-20-8), Dopamine hydrochloride (DA·HCl, CAS #62-31-7), Acrylamide (AM, CAS #79-06-1), N,N′-Methylenebisacrylamide (BIS, CAS # 110-26-9), Sodium persulfate (SPS, CAS #7775-27-1), Sodium hydroxide (NaOH, CAS #1310-73-2), Acrylic acid (AAc, CAS # 79-10-7), Poly (vinyl alcohol) (PVA, CAS #9002-89-5), [2-(Methacryloyloxy)ethyl]dimethyl-(3-sulfopropyl)ammonium hydroxide (SBMA, CAS #3637-26-1), and Ethylene glycol (EG, CAS #107-21-1) were purchased from Sigma-Aldrich. All reagents were used as received unless otherwise indicated. Dulbecc's Modified Eagle Medium (DMEM) and Fetal bovine serum (FBS) were purchased from Gibco.

### 2.2 Preparation of conducting polymer hydrogels

IPNCH:

Pre-procedure: Freeze-dried PEDOT:PSS aqueous solution.

IPNCH@CAPAM: Firstly, κ-Carrageenan CA (0.05 g) is dissolved fully in deionized water (5 mL) at 60 °C, and NaOH (300 μL, 0.5 g mL^−1^) is added to form an alkaline environment. Then add dopamine hydrochloride (DA·HCl, 10 mg). After 20 min of reaction, 0.06 wt% freeze-dried PEDOT:PSS is dispersed into the solution. Finally, 2.2 M of monomer acrylamide (AM), 0.06 wt% of crosslinker N,N′-Methylenebisacrylamide (BIS) and 0.16 wt% of initiator sodium persulfate (SPS) are completely dissolved in the above solution, and then poured into the corresponding mold to self-cure into hydrogel.

IPNCH@PAM: Add NaOH (300 μL, 0.5 g mL^−1^) to deionized water (5 mL) to form an alkaline environment. Then add 10 mg DA·HCl. After 20 min of reaction, 0.06 wt% freeze-dried PEDOT:PSS is dispersed into the solution. Finally, 2.2 M of monomer AM, 0.06 wt% of crosslinker BIS and 0.16 wt% of initiator SPS are completely dissolved in the above solution, and then poured into the corresponding mold to self-cure into hydrogel.

IPNCH@PAAc: Add NaOH (300 μL, 0.5 g mL^−1^) to deionized water (5 mL) to form an alkaline environment. Then add 10 mg DA·HCl. After 20 min of reaction, 0.06 wt% freeze-dried PEDOT:PSS is dispersed into the solution. Finally, 2.2 M monomer acrylic acid, 0.06 wt% crosslinker BIS and 0.16 wt% initiator SPS are completely dissolved in the above solution, and then poured into the corresponding mold to self-cure into hydrogel.

IPNCH@PAAc-PVA: Dissolve 0.55 M PVA in deionized water (5 ml) and heat to 80–90 °C to dissolve completely. NaOH (300 μL, 0.5 g mL^−1^) was added to form an alkaline environment. Then add 10 mg DA·HCl After 20 min of reaction, 0.06 wt% freeze-dried PEDOT:PSS is dispersed into the solution. Finally, 1.65 M monomer acrylic acid, 0.06 wt% crosslinker BIS and 0.16 wt% initiator SPS are completely dissolved in the above solution, and then poured into the corresponding mold to self-cure into hydrogel.

PSCH:

PEDOT:PSS aqueous solution is vigorously stirred for 6 h, and then 25 wt% ionic compound, 0.06 wt% BIS, and 0.16 wt% ammonium persulfate (APS) were added and stirred for an additional 24 h to ensure homogeneous mixing. The resulting solution was then poured into a mold, covered with a glass slide, and heated on a hot plate at 90 °C for 45 min. During the thermal curing process, APS decomposes to generate free radicals that initiate polymerization of zwitterions, resulting in the formation of a covalently cross-linked network interpenetrated with PEDOT:PSS micelles. The ionic liquids function as dopant molecules, softening the PSS shell and promoting aggregation-induced phase separation of PEDOT.

PCH:

PEDOT: PSS aqueous solution is vigorously stirred for 6 h, and then a polar solvent, such as ethylene glycol (EG), is added to 25 vol% of the final solution. After a further 24 h of stirring at room temperature, the mixed solution is poured directly into the template as a solvent and dried at 60 °C for 24 h, followed by multiple anneals at 130 °C (three cycles of 30 min each) and rehydration to obtain pure PEDOT:PSS hydrogels.

### 2.3 Mechanical characterization

The elastic and viscous moduli of hydrogels were measured using a MCR 301 rheometer (Anton Paar GmbH, Graz, Austria). For a frequency-sweep test, the shear modulus was measured in a frequency range of 0.1–10 Hz with 1 % strain. The mechanical properties of the hydrogels were tested by using a WDW-3020 universal testing machine (Changchun Kexin Test Instrument Co., Ltd., Changchun, China) with a 100 N load cell. The tensile test was performed on the rectangular sample (25 mm × 8 mm × 2 mm) at a tensile rate of 60 mm min^−1^. The cyclic stretching rate was 60 mm min^−1^ with a 200 % tensile ratio. The tensile stress (*σ*) was calculated using the following equation:

(1) $$\begin{array}{c} \sigma =\frac{F}{A} \end{array}$$

where F is the tensile load, and A is the cross-sectional area.

The extension ratio (*λ*) was calculated using the following equation:

(2) $$\begin{array}{c} \lambda =\frac{l}{l_{0}} \end{array}$$

where l is the extended length, and $l_{0}$ is the original length.

To measure the interfacial toughness of hydrogels with different organs, the organs were cut after freezing at −20 °C. The hearts were cut into flat sheets with dimensions of 1 cm × 1 cm × 5 mm. The skins were cut into flat sheets with a length and width of 1 cm, and their original thickness was retained. The organ specimens were kept in PBS at 4 °C to retain in a wet condition before use. Then the hydrogels were attached to hearts, skins, and glasses. The prepared samples were tested by the standard 90° peel test with a WDW-3020 universal testing machine (Changchun Kexin Test Instrument Co., Ltd., Changchun, China). All tests were performed with a constant peeling speed of 30 mm min^−1^. Peel-off strength was calculated by dividing the plateau force by the width of the adhered samples [37].

### 2.4 Electrical and electrochemical characterization

The electrical and electrochemical properties of hydrogels were measured by the CHI660E electrochemical workstation (Shanghai Chenhua Instrument Co., Ltd., Shanghai, China). To measure conductivity, a hydrogel was filled between two parallel copper electrodes which were connected within an electrical loop, and the corresponding voltage data was obtained by applying 5 mA DC current. The electrical resistance (R) was calculated using Ohm's law:

(3) $$\begin{array}{c} R=\frac{U}{I} \end{array}$$

where U is the voltage, and I is the current. Subsequently, the conductivity (σ) was determined according to the following equation:

(4) $$\begin{array}{c} \sigma =\frac{1}{\rho }=\frac{L}{RA} \end{array}$$

where ρ is the resistivity, L is the distance between the two copper electrodes, and A is the cross-sectional area of the hydrogel sample (calculated as length × width). In general, hydrogels were fabricated using molds of 10 × 10 × 1 mm in size. All the actual geometric parameters, including the length and width of the material and the electrode spacing, have been precisely measured with a vernier caliper to ensure their accuracy.

The interfacial resistance (R_b_) was measured by adhering the hydrogel electrode in the middle of two reference electrodes (R_a_ and R_c_), ensuring equal spacing (L_ab_ = L_bc_). The resistances at three points (R_ab_, R_bc_, and R_ac_) were measured, and the interfacial resistance R_b_ was calculated using:

(5) $$\begin{array}{c} R_{b}=\frac{R_{ab}+R_{bc}-R_{ac}}{2} \end{array}$$

The value reflects the contact resistance at the hydrogel–electrode interface, providing insight into the stability and quality of electrical contact in bioelectronic applications.

To measure the electrical impedance (EIS), the hydrogel was sandwiched between two steel electrodes with an overlapped area (10 mm × 10 mm) and equilibrated in PBS before tests. The electrodes were connected to the electrochemical workstation. Impedance measurements were taken between 10^−1^ and 10^4^ Hz with an amplitude of 5 mV. To measure the cyclic voltammetry (CV), a hydrogel film was clamped to act as an electrode with a coating area of 1 cm^2^. The CV was measured from open-circuit voltage (OCV) −0.2 to OCV+0.2 V at 100 mV s^−1^. The charge storage capacity (CSC) was calculated from the CV data using the following equation:

(6) $$\begin{array}{c} \mathrm{CSC}=\int_{E2}^{E1}\frac{i\left(E\right)}{2vA}dE \end{array}$$

where v is the scan rate, E1 and E2 are the lower and upper potential limits, i(E) is the current at each potential, and A is the area of the electrode.

### *2.5* *In vitro* biocompatibility

Hydrogels were prepared and immersed in medium to remove the unreacted monomers. Simultaneously, ultraviolet irradiation was applied for a 24-h sterilization treatment. Typically, 10 mL of Dulbecco's modified Eagle medium (DMEM) was soaked with 1 g of the above samples at 37 °C for 24 h, respectively, and then supplemented with 10 % v/v fetal bovine serum and 100 U mL^−1^ penicillin-streptomycin before use. The supplemented DMEM without incubating the above samples was used as a control. Except for the blank group, BHK21 cells of logarithmic growth stage were inoculated on 96-well plates with a density of 5000 cells. After cell adhesion (about 4 h), the experimental groups were replaced with 100 μl extraction medium of different hydrogels, and continued to culture for 24 and 72 h. After culture, 100 μl fresh DMEM was replaced and 10 μl CCK-8 reagent was then added to each well. After continuous culture for 2 h, the optical density (OD, representing cell density) of each well was measured at 450 nm using an enzyme-labeler. The cell viability was quantified by the formula:

(7) $$\begin{array}{c} \text{Cell}\;\text{viability}=\frac{A_{s}-A_{b}}{A_{c}-A_{b}}*100\% \end{array}$$

where $A_{s}$ is the absorbance of the experimental group, $A_{b}$ is the absorbance of the blank group (without cells), and $A_{c}$ is the absorbance of the control group (supplemented DMEM without incubating the hydrogels).

### *2.6* *In vivo* biocompatibility

The animal experiments were performed in accordance with the relevant rules and regulations, and were reviewed and approved by the Institutional Animal Care and Use Committee (IACUC) of Tsinghua University (approval number: 24-LY2, approval date: 2024-1-18).

The hydrogels were cut into samples with a length and width of 1 cm for implantation, and the sham operation group was set as a control group. After prepared aseptically, all samples were immersed in PBS for 24 h to remove the unreacted monomers, and simultaneously sterilized by UV light before use. The animals were anesthetized with 10 % chloral hydrate, and the implants were placed in the dorsal subcutaneous area without overlapping with each other. The animals were killed after 7-day and 14-day implantation, and the subcutaneous areas of interest were excised and fixed with paraformaldehyde for 24 h for histological analysis. Fixed tissue samples were placed in 70 % ethanol and submitted for histological processing and H&E staining.

### 2.7 Monitoring of epidermal electrophysiological signal

Epidermal EMG, ECG, and EEG signals were detected by using ESP32, AD8232, and TGAM module (Jiangsu Xinweilai Technology Co., Ltd., Jiangsu, China) on the volunteer participant. Informed written consent from the participants was obtained prior to the research. Because this experiment did not require any samples from the human body, they were not required by the approval from a national or institutional ethics board/committee. To obtain the EMG, the hydrogels were adhered to both ends of the tested muscle as the working and reference electrodes. To obtain the ECG, the hydrogels were adhered to both wrists. The hydrogels were adhered to both ears and above the left eyebrow to obtain EEG. Once the signals were extracted, they were then processed with a band-pass filter using Matlab. The signal-to-noise ratio (SNR) was then calculated according to the standard definition in signal-processing literature [38,39]:

(8) $$\begin{array}{c} SNR=20*log_{10}\left(\frac{V_{signal}}{V_{noise}}\right) \end{array}$$

where $V_{signal}$ is the root-mean-square (RMS) amplitude of the signal segment, and $V_{noise}$ is the RMS amplitude of the noise segment. The preprocessing procedures for various electrophysiological signals, along with the methods used to identify and process signal and noise segments, are presented in Table S5 and the Supplementary data processing program.

### 2.8 Monitoring of implantable electrophysiological signal

All animal experiments were conducted under protocols approved by the Institutional Animal Care and Use Committee of Tsinghua University. The PhysioTel™ fully implantable telemetry system (Data Sciences International, St. Paul, MN, USA) was combined with hydrogel electrodes for implantable ECG and EMG recording. The wires of the implant PhysioTel™ and the hydrogel electrodes were co-formed in the mold so that the tough wires were completely covered by the hydrogels. Four hydrogel electrodes were implanted on each side of the dorsal muscle and on both ends of the line at the apex of the heart. The surgical details are described below: Adult BALB/c mice were only used to evaluate the performance of the hydrogel electrodes. The mice were anesthetized with pentobarbital (1 wt%, 80 mg/kg), and an intermuscular pocket was created between the muscles of the internal oblique and transversus abdominus muscles through aponeurosis incision. The transmitter was inserted and secured with nylon sutures with the antenna perpendicular to midline in the lower abdominal muscle fascia of the external oblique, and then hydrogel electrodes were implanted into the set sites and the skin was sutured. After implantation, the muscles and skin returned to normal. ECG and EMG signals were measured for 3 weeks by turning on the machine for 30 min every week.

### 2.9 Material characterization

SEM: The prepared hydrogel is flash-frozen in liquid nitrogen, and brittle in liquid nitrogen, exposing the cross section. Pay attention to avoid contacting or squeezing the section with tweezers when brittle, so as not to affect its pore structure. The hydrogel is then quickly transferred to a freeze-dryer under vacuum conditions of 4 × 10^−3^ Torr to dry for 24 h. Cross-sectional images were obtained by S-4800 field emission scanning electron microscope (Hitachi High-Technologies Corporation, Tokyo, Japan).

The Fourier-transform infrared (FTIR) spectra were obtained on a Nicolet 6700FTIR spectrometer (Thermo Scientific, Technology Instrument Co., Shanghai, China) over a scan range of 400–4000 cm^−1^. UV–Vis absorption spectra were recorded using a AvaSpec-ULS4096CL-EVO spectrometer (Avantes BV, Apeldoorn, The Netherlands) over the relevant wavelength range. Raman spectra were acquired on a LabRAM HR800 confocal Raman microscope (HORIBA Ltd., Kyoto, Japan) equipped with a 633 nm laser, and the spectra were collected under ambient conditions. Atomic force microscopy (AFM) measurements were performed in tapping mode using MFP-3D-SA atomic force microscope (Asylum Research, an Oxford Instruments Company, Santa Barbara, CA, USA) with silicon cantilevers to assess the surface topography and phase distribution of the samples. X-ray diffraction (XRD) patterns were collected on a D8 ADVANCE X-ray diffractometer (Bruker AXS GmbH, Karlsruhe, Germany) using Cu-Kα radiation operated at 40.0 kV and 120 mA.

### 2.10 Statistical analysis

GraphPad Prism software (GraphPad Software, San Diego, CA, USA) and Origin software (OriginLab Corporation, Northampton, MA, USA) were used for statistical analysis. All data are expressed as the mean ± standard deviation (SD). Error bars indicate SD. Tukey's multiple comparison test were employed to assess the statistical significance with threshold of ∗P < 0.05, ∗∗P < 0.01, ∗∗∗P < 0.001. Significance was assigned at p < 0.001.

## 3 Results and discussion

### 3.1 Fabrication and exploration of hydrogels in synergistic mechanical and electrical performances

**Fig. 2:**
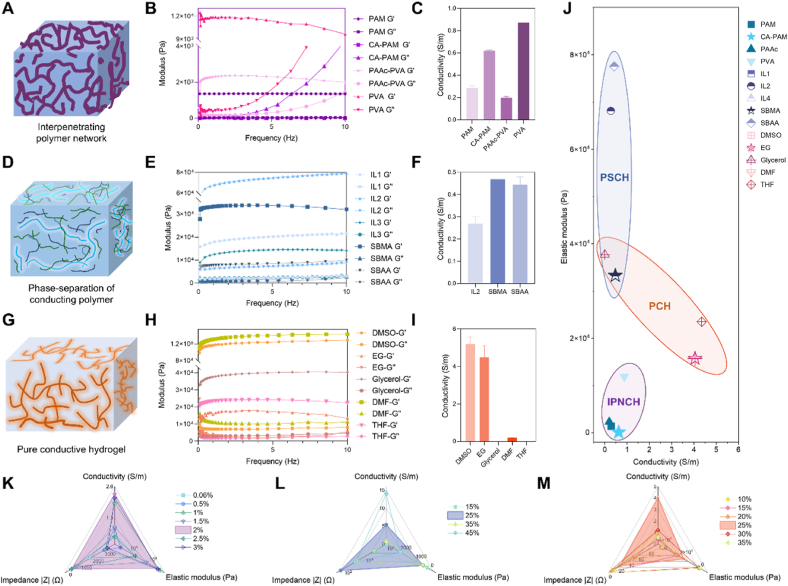
Rational design of flexible and stretchable modification strategies for conducting polymer hydrogels. A) Schematic illustration of conducting polymer based interpenetrating network conductive hydrogels (IPNCHs). B) Comparison of modulus between IPNCHs. C) Comparison of conductivity between IPNCHs. D) Schematic illustration of conducting polymer-based phase separation conductive hydrogels (PSCHs). E) Comparison of modulus between PSCHs. F) Comparison of conductivity between PSCHs. G) Schematic illustration of pure conducting polymer hydrogels (PCHs). H) Comparison of modulus between PCHs. I) Comparison of conductivity between PSCHs. J) Performance comparison and screening of IPNCHs, PSCHs and PCHs in different formulations. K-M) Comparison and screening of IPNCH@CAPAM, PSCH@SBMA and PCH@EG properties of varying raw material ratios.

To harmonize the electrical properties of conducting polymers and the mechanical properties of hydrogels, three modification strategies were explored to develop conducting polymer hydrogels with high flexibility, stretchability, and conductivity. The first strategy involves mixing or in-situ polymerization of conducting polymers within non-conductive hydrogel templates to form a typical interpenetrating polymer network (IPN) (Fig. 2A–C). PEDOT:PSS was utilized as the model polymer due to its dual conductivity and biocompatibility [16,40]. Four types of typical non-conductive hydrogel templates (polyacrylamide (PAM), polyacrylamide-carrageenan (CAPAM), polyacrylic acid (PAAc), and polyvinyl alcohol (PVA)) were used as support substrates. These templates possess inherent softness and moisture due to their porosity and high water content, facilitating conformal contact with biological tissues. This approach combines the electrical conductivity of conducting polymers with the mechanical properties of non-conductive substrates. The second strategy introduces molecules with sulfonates or sulfonimide anions as effective dopants to modulate phase separation and promote polymer aggregation (Fig. 2D–F) [[41], [42], [43], [44]]. Ionic compounds, including three types of ionic liquids (IL1: 1-ethyl-3-methylimidazolium ethyl sulfate (EMIM:ES), IL2: 4-(3-butyl-1-imidazolium)-1-butanesulfonic acid triflate (BIM:BSA3), and IL3: 1-butyl-3-methylimidazolium tetrafluoroborate (Bmim:BF4)) and two types of zwitterions [2-(Methacryloyloxy)ethyl]dimethyl-(3-sulfopropyl)ammonium hydroxide (SBMA), and 3-[dimethyl-[3-(2-methylprop-2-enoylamino)propyl]azaniumyl]propane-1-sulfonate (SBAA)), were incorporated into the conducting polymer hydrogels. Owing to their excellent solubility in both water and the polymer matrix, these compounds effectively soften the PSS domains within PEDOT:PSS. The highly acidic sulfonate or sulfonimide groups engage in strong electrostatic interactions and hydrogen bonding with the positively charged PEDOT segments and polar groups along the polymer chains. This dual interaction not only stabilizes the doped state but also induces a controlled, uniform phase separation and polymer aggregation, resulting in an interconnected conductive network, achieving a synergistic effect of conductivity and stretchability. The third modification strategy is to introduce polar solvent additives into the conducting polymer, followed by controlled dry-annealing to yield pure conducting polymer hydrogels (Fig. 2G–I) [[45], [46], [47]]. Five types of polar solvents (dimethyl sulfoxide (DMSO), ethylene glycol (EG), glycerol, N,N-dimethylacetamide (DMF), and tetrahydrofuran (THF)) were added to the conducting polymer systems. These polar solvents can be removed to a certain high extent through the heating, dry-annealing, and rehydrating process, to produce nearly pure hydrogels. Solvents such as DMSO can still be partially removed at elevated temperatures, although it is not typically considered volatile. This removal is not simply boiling volatilization, but is due to diffusion and desorption processes, as well as differences in interaction with the substrate. These polar solvents improve the crystallinity and order of the conducting polymer nanocrystals, enhancing electrical properties. In addition, pure conducting polymer hydrogels, free from other components, avoid issues such as impaired conductivity, uneven mechanical and electrical properties, and cytotoxicity from other fillers in the hydrogel network. All three strategies were employed to prepare conducting polymer hydrogels, resulting in 14 different conducting polymer hydrogels. These were further characterized for their mechanical and electrical properties to identify systems suitable for bioelectronic applications.

The mechanical compatibility of conductive hydrogels with cells or tissues is a crucial factor to consider for their bioelectronic applications. Improved mechanical properties could potentially reduce mechanical mismatches between hydrogels and various human tissues. Oscillatory testing was performed to investigate the viscoelastic characteristics of conducting polymer hydrogels under the above three cross-linking mechanisms over a frequency from 0.1 to 10 Hz. The elastic modulus (G’) of all the conducting polymer hydrogels exceeded the viscous modulus (G”), conforming to their gel-like nature. Specifically, in the PEDOT:PSS hydrogel systems forming IPNs, except for the hydrogel based on PVA, the elastic modulus of the other three systems was lower than 3.0 kPa, which is close to the modulus of soft brain tissue (0.2–3.1 kPa) (Fig. 2B) [48,49]. Among them, the hydrogel IPNCH@CAPAM had elastic modulus of only 0.1 kPa, exhibiting ultra-soft properties. The brain-level softness of the hydrogel is because of the polymerization inhibition effect of catechol groups of polydopamine, which restrains the chain growth of PAM, reducing the entanglement between long PAM chains. The uniformly dispersed conducting polymer interacts non-covalently with the CA and PAM polymer chains in the hydrogel, dissipating energy and maintaining the integrity of the hydrogel main network during deformation, resulting in soft and stretchable characteristics. This suggests it could function as a bridge for mechanical matching and improve the long-term biomechanical interaction between bioelectronic devices and biological tissues. The elastic modulus of phase separation PEDOT:PSS hydrogels constructed from ionic compounds, which ranged from 12 to 77 kPa, covering the modulus range of most relevant tissues (1–100 kPa) (Fig. 2E) [50,51]. The partial aggregation of conjugated polymers induced by ionic compounds, while softening the dopant PSS domain, effectively reduced elastic modulus of conducting polymer hydrogels, making them highly malleable. For pure conducting polymer hydrogel systems constructed with polar solvents, the elastic modulus of such systems was generally high, in the range of 15–130 kPa, due to the lack of a supporting matrix (Fig. 2H). However, such a modulus is still several orders of magnitude lower than conventional rigid materials in bioelectronic devices [9,52], and even lower than that of soft elastomers such as polydimethylsiloxane (PDMS) (1–10 MPa) [53,54]. This is due to the fact that the polar solvent as additive has facilitated the recrystallization of PEDOT-rich nanofibrils and chain rearrangement of PEDOT:PSS during the dry-annealing process [55,56]. The achieved uniform distribution of rigid conjugated PEDOT-rich crystalline regions and PSS-rich soft regions could result in excellent mechanical compliance in biological tissues [57,58]. These results confirmed the advantages of hydrogels in matching the mechanical properties of biological tissues.

In addition to mechanical properties, the electrical properties of hydrogels in physiological environments, matched to that of biological tissues, are crucial for ensuring stable two-way communication between devices and tissues. The conductivity and impedance of conducting polymer hydrogels were measured via an electrochemical workstation (Fig. 2C,F,2I, and Fig. S1). Except for the hydrogels with poor mechanical properties (PSCH@IL1 and PSCH@IL3), which were extremely fragile and difficult to test for conductivity, the conductivity of IPNCHs and PSCHs was distributed in the range of 0.19–0.87 S/m. Some PCHs exhibited significantly higher conductivity, reaching up to 5.21 S/m (PCH@DMSO), attributed to the removal of the non-conductive insulation components. Additionally, the introduction of polar solvents facilitated the curling of the conjugated polymer chains, enhancing their crystallinity and establishing robust connectivity between the conjugated polymers.

The three strategies are mutually complementary – the IPNCH forms the soft foundation, the PSCH refines the conductive pathways, and the PCH maximizes conductivity – thereby collectively covering a broad mechanical-electrical spectrum that can be tailored for diverse bioelectronic applications. As shown in Table S1, the different formulations within each strategy offer various advantages and trade-offs. Relying on the test results for the modulus and conductivity of 14 conducting polymer hydrogel formulations, three formulations stood out for their preferred properties: IPNCH@CAPAM, PSCH@SBMA, and PCH@EG (Fig. 2J). Compared with other reported hydrogels, the conductivity of the conducting polymer hydrogels could fully meet the requirements for electrophysiological signal monitoring and other bioelectronic applications (Table S6) [[59], [60], [61], [62], [63]]. And these improvements underscore the effectiveness of our screening system in optimizing multi-dimensional properties and validate its scientific rigor.

For the IPNCH@CAPAM, PSCH@SBMA, and PCH@EG with outstanding performance, subsequent selection and optimization of the best formulation were carried out to achieve synergistic enhancement (Fig. 2K–M, Fig. S2–S6). In general, the relationship between hydrogel properties and component proportions showed consistency. Specifically, a higher conducting polymer content, or improved rearrangement of conjugated polymer nanofibrils due to phase separation, tended to increase the hydrogel's conductivity while reducing its impedance at physiologically relevant frequencies. However, the introduction of more rigid conjugated PEDOT-rich regions inevitably led to an increase in the hydrogel's modulus. Based on the experimental results, the raw material ratios yielding high conductivity, low impedance, and low elastic modulus were selected as follows: IPNCH@CAPAM with 2 wt% PEDOT:PSS, PSCH@SBMA with 25 wt% SBMA, and PCH@EG with 25 vol% EG. As showed in Table S2, the three strategies collectively cover a broad mechanical-electronic landscape: IPNCH@CAPAM occupies the ultra-soft region (0.28 kPa, 1.99 S/m), ideal for neural interfaces; PSCH@SBMA bridges moderate modulus (0.44 kPa) with peak conductivity (5.25 S/m), suited for cardiac/muscular implants; PCH@EG achieves skin-matching modulus (15 kPa) while retaining high conductivity (3.79 S/m), optimal for epidermal electronics. This complementary coverage ensures adaptability to varied tissue interfaces. The cross-linking efficiency of the three modification strategies was further measured as important parameter in evaluating the synthesis efficiency and application potential (Fig. S20). These formulations offer a balance of desirable properties, making them suitable for bioelectronic applications.

### 3.2 Characterization of crosslinking mechanisms

**Fig. 3:**
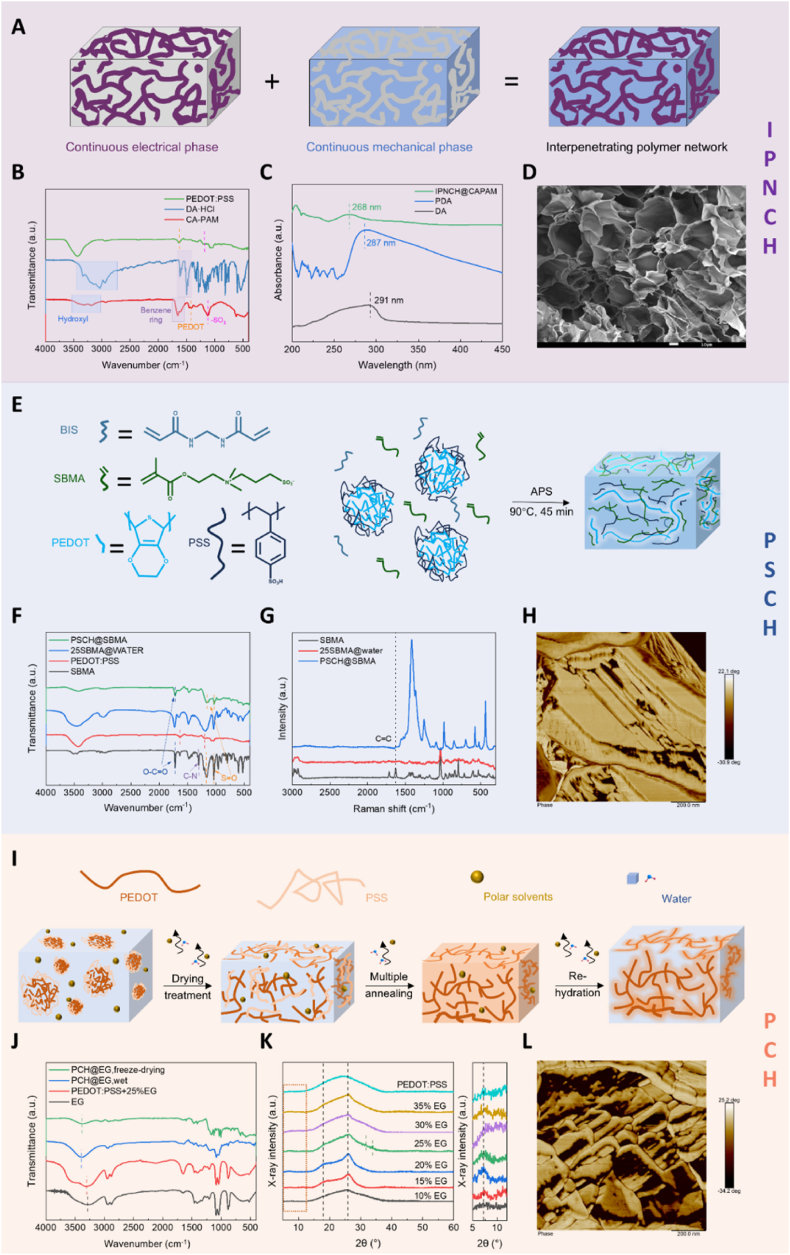
Characterization of crosslinking mechanisms of conducting polymer hydrogels. A) Schematic illustration of crosslinking mechanism of IPNCH@CAPAM. B) Fourier transform–IR (FTIR) spectroscopy of IPNCH@CAPAM. C) Ultraviolet–visible (UV–Vis) spectroscopy of IPNCH@CAPAM. D) SEM image of the porous structure of IPNCH@CAPAM, scale bar: 10 μm. E) Schematic illustration of crosslinking mechanism of PSCH@SBMA. F) Fourier Transform infrared spectroscopy (FTIR) of PSCH@SBMA. G) RAMAN spectroscopy of PSCH@SBMA. H) AFM images of the phase distribution of PSCH@SBMA, scale bar: 200 nm. I) Schematic illustration of crosslinking mechanism of PCH@EG. J) Fourier Transform infrared spectroscopy (FTIR) of PCH@EG. K) X-Ray diffraction (XRD) pattern of PCH@EG. L) AFM images of the phase distribution of PCH@EG, scale bar: 200 nm.

Based on the selected hydrogel formulations, the crosslinking mechanisms of the conducting polymer hydrogel systems constructed under these three modification strategies were analysed and verified for bioelectronic applications. Firstly, for the interpenetrating polymer network hydrogel IPNCH@CAPAM with a yield of 95.22 %, as shown in Fig. 3A, dopamine (DA) was initially polymerized in an alkaline carrageenan (CA) solution containing NaOH to produce polydopamine (PDA). The cured PEDOT:PSS obtained by freeze-drying was then combined with acrylamide (Am, monomer), N,N′-methylenebisacrylamide (BIS, crosslinker), and sodium persulfide (SPS, initiator) to spontaneously polymerize to a conducting polymer hydrogel with an interpenetrating network within 3 min (Fig. S7). Scanning electron microscope (SEM) images of the freeze-dried IPNCH@CAPAM revealed a typical three-dimensional (3D) interconnected porous microstructure, which provides condition for the hydrogels to withstand large deformations (Fig. 3D, Fig. S13). Fourier Transform infrared spectroscopy (FTIR) also confirmed the successful synthesis of the IPNCH@CAPAM hydrogel, indicated by additional peaks from catechol groups (a broad hydroxyl stretching vibration absorption peak at 3500 cm^−1^,benzene ring stretching vibration absorption peaks at 1450-1600 cm^−1^), and PEDOT:PSS (a -SO_3_ characteristic peak at 1122 cm^−1^, and a PEDOT characteristic peak at 1458 cm^−1^) (Fig. 3B). Moreover, the characteristic absorption peak of catechol groups at 280 nm observed in ultraviolet–visible (UV–Vis) spectroscopy further confirmed successful incorporation of PDA into IPNCH@CAPAM (Fig. 3C) [64]. The XRD patterns reveal a broad and diffuse peak around 2θ ≈ 24° after PEDOT is incorporated into the IPNCH@CAPAM system (Fig. S8A). This indicates increased interaction between PEDOT and the hydrogel matrix, which contributes to greater structural disorder. The resulting amorphous nature facilitates the formation of a flexible conductive network, improving both the electrical and mechanical properties of the system. Conducting polymer was dispersed throughout the hydrogel network of CA and PAM chains, creating conductive pathways. Additionally, the binding energy of the C-S bond in PEDOT in the XPS spectra shows a slight shift in IPNCH@CAPAM (Fig. S8B). This shift suggests physical interactions, such as hydrogen bonding or π-π interactions, between PEDOT and components like CA, PAM, or PDA. These interactions alter the local environment of PEDOT, further enhancing the system's electrical conductivity and mechanical strength. PDA and PEDOT formed an electron donor-acceptor system, with PEDOT facilitating electron transfer to quinone and preventing further oxidation of catechols. The dynamic redox-active system within the hydrogel network maintained adequate catechols, endowing the hydrogel with adhesiveness. Simultaneously, the polymerization inhibition of catechol groups in PDA inhibited the chain growth of PAM, reducing the entanglement between the long chains of PAM. The physical interaction between the linear chain of CA and the PAM chain improved the stretchability and toughness of the hydrogel. Therefore, the construction of IPNCH@CAPAM has proven to be an excellent strategy for obtaining flexible and stretchable conducting polymer hydrogels.

For the second modification strategy with a yield of 89.84 %, involving the introduction of ionic compounds, the PSCH@SBMA hydrogel was successfully constructed from a conducting polymer network and a multifunctional poly (sulfobetaine) (pSB) network (Fig. 3E). SBMA can self-polymerize in water as SBMA@water via a radical mechanism, the association-induced polymerization effect leads to the formation of nanosized associations in concentrated aqueous solution of SBMA monomer, augmenting the effective monomer concentration and enabling successful polymerization [65]. Ammonium persulfate (APS) functions as a thermal initiator, and N,N′-methylenebisacrylamide (BIS) acts as a crosslinking agent to form a covalently cross-linked pSB network under high-temperature heating for about 45 min (Fig. S14–15). The formation of a covalently cross-linked pSB network within PSCH@SBMA was investigated using FTIR spectroscopy (Fig. 3F). The SBMA monomer exhibited a C–N characteristic absorption peak at 1305 cm^−1^, which was negligible in the spectra of SBMA@water and PSCH@SBMA due to the electrostatic shielding effect of the –N ${\left({\text{CH}}_{3}^{+}\right)}_{2}^{-}$ group on the -${\text{SO}}_{3}^{-}$ group after polymerization. The peaks from pSB appeared at 1731 cm^−1^ (O-C=O stretching vibration), 1160 cm^−1^, and 1036 cm^−1^ (S=O asymmetric stretching vibration) in the spectrum of both SBMA@water and PSCH@SBMA. Raman peaks at 1637 cm^−1^ contributed to C=C bonds also disappeared in the spectra of SBMA@water and PSCH@SBMA (Fig. 3G). Characterizing PSCH@SBMA with different SBMA contents, it can be found that the electrostatic shielding provided by the pSB network reduces the attraction between PEDOT and PSS, promotes the transition from the compact core-shell structure to the more extended PEDOT chain, ultimately facilitating the formation of a percolated conductive network (Fig. S9). X-Ray diffraction (XRD) results showed the characteristic peak of monomer in the PSCH@SBMA spectrum disappeared, and a characteristic peak at 2θ = 19 °consistent with that of SBMA@WATER polymer appeared (Fig. S9A). Meanwhile, the large packet peak might cover the characteristic peak of PEDOT. For PSCH@SBMA with 5–25 wt% SBMA, a new peak appeared at 2θ = 20–30°, which might be attributed to the changes in the crystal structure, more pronounced phase separation, and improved charge transport performance. Phase images measured by AFM also showed phase separation between PEDOT-rich domains (bright) and PSS-rich domains (dark) (Fig. 3H). The core-shell structure of PEDOT: PSS transitioned to longer chains, with 25 wt% PSCH@SBMA exhibiting a clearer conductive path (Fig. S18, and Table S4), facilitating the formation of a more efficient electron transfer pathway. Additionally, the covalently cross-linked pSB network was semi-interpenetrated with the conducting polymer network, further imparting PSCH@SBMA with tissue-like mechanical properties. Moreover, the strong electrostatic interaction between pSB and charged groups on the tissue surface conferred bioadhesion to PSCH@SBMA. Consequently, the inherent brittleness of conducting polymer was overcome, and the characteristics of high flexibility and stretchability were introduced into the phase separation conducting polymer hydrogel.

For the final modification strategy, a pure conducting polymer hydrogel with a yield of 85.48 % was created by introducing a polar solvent. Ethylene glycol (EG) was added to the conducting polymer and dried for 24 h. Gelation was basically completed in about 4 h, and then subjected to controlled dry annealing and rehydration to obtain PCH@EG hydrogel (Fig. 3I, Fig. S7D, and Fig. S16-17). The characteristic FTIR spectral absorption peaks for EG (3100-3500 cm^−1^, hydroxyl absorption peak) were clearly observed in EG-containing aqueous PEDOT:PSS solutions (Fig. 3J). However, these absorption peaks diminished significantly in FTIR spectra of dry-annealed PEDOT:PSS, and further disappeared in PCH@EG after freeze-drying. This indicated that the added EG had been mostly removed during the dry-annealing process, yielding a pure conducting polymer hydrogel, and the nuclear magnetic resonance (NMR) spectroscopy of PCH@EG further demonstrated the removal of EG (Fig. S10). In aqueous colloidal dispersion, PEDOT:PSS tends to form micellar microstructures consisting of hydrophobic PEDOT-rich cores and hydrophilic PSS-rich shells. In pristine solutions, the lack of interconnected conducting polymer nanofibrils leads to dissociation into fragmented microgels in a wet environment [56,66]. Adding polar solvents like ethylene glycol effectively extends conducting polymer microgel particles from the trapped and/or folded states into linear long chains. The process facilitates the formation of larger crystalline PEDOT-rich nanofibrils and interchain entanglements between PSS chains during the drying process [67,68]. XRD analysis was performed to elucidate the structure of PCH@EG (Fig. 3K). It was found that PCH@EG with varying EG content all contained diffraction peaks of PEDOT at 2θ = 7°, 18°, 26°. The peak at 2θ = 18° in Fig. 2I might be obscured by the wide background peak at 26°, and the sub-peaks varied with the changes in EG content, indicating structural rearrangements in the hydrogels due to alterations in doping levels [[69], [70], [71]]. For 25 vol% PCH@EG, a new peak emerged at 2θ = 30–35°, attributed to changes in crystal structure, more pronounced phase separation, and improved charge transport performance. Raman spectroscopy further supported these findings, revealing enhanced π–π stacking interactions among PEDOT chains (Fig. S11). This structural refinement correlates with the AFM images, where the 25 vol% PCH@EG sample displayed brighter and more continuous PEDOT-rich domains, indicative of well-defined nanoscale phase separation and improved electrical pathways (Fig. 3L, Fig. S19). AFM topography also showed increased surface roughness (Ra) (Table S4), consistent with the emergence of distinct nanofibrillar features. Additionally, thermogravimetric-mass spectrometry (TG-MS) and high-performance liquid chromatography (HPLC) analyses confirmed the effective removal of EG after annealing and soaking (Fig. S12), validating its role as a transient processing aid that promotes chain rearrangement without leaving behind residual solvent that could impede performance. Redesigning the phase distribution of conducting polymer is a key strategy for transforming it into flexible and stretchable hydrogels. A strong interconnection between the conductive and hydrophilic domains provides excellent electrical conductivity and aqueous stability [72].

Collectively, these structural analyses confirmed that specific strategies have their characteristic microstructures, which in turn lead to variations in the mechanical and electrical properties of the hydrogels constructed under different strategies.

### 3.3 Mechanical performance of the conducting polymer hydrogels

The conducting polymer hydrogels prepared using the three strategies demonstrated distinct mechanical behaviours that are optimized for various bioelectronic applications. The inherent softness and physical properties of hydrogel systems offer effective means to bridge mechanical mismatch between electronic devices and soft biological tissues, addressing the limitations of traditional metal- or polymer-based bioelectronics.

**Fig. 4:**
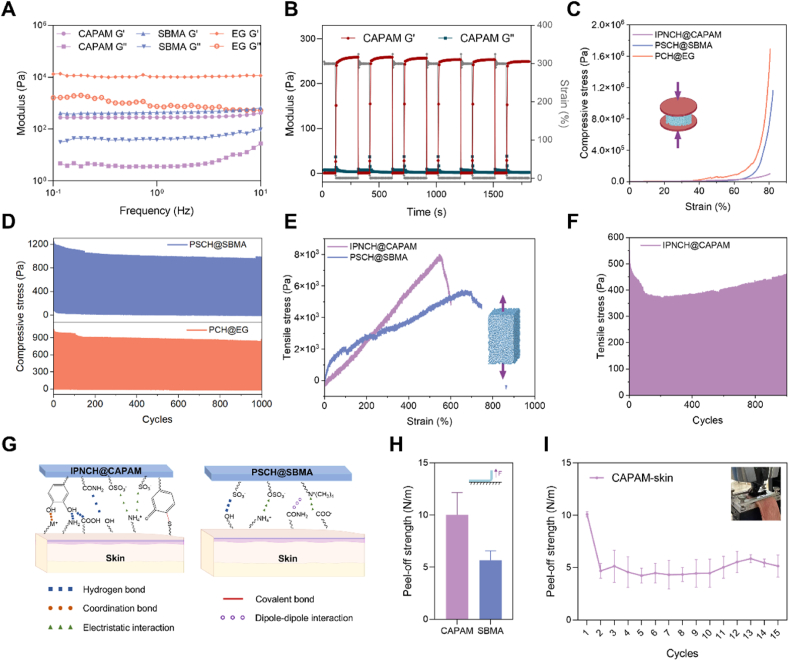
Mechanical properties of the conducting polymer hydrogels IPNCH@CAPAM, PSCH@SBMA, and PCH@EG. A) Rheological analysis showing the elastic modulus (G′) and viscous modulus (G″) of the hydrogels as a function of frequency (0.1–10 Hz). B) The G′ and G″ of the IPNCH@CAPAM when alternate step strain is switched from small strain (0.5 %, 200 s) to large strain (300 %, 100 s) at a fixed frequency (1 Hz). C) Compressive stress-strain curves for IPNCH@CAPAM, PSCH@SBMA, and PCH@EG. The inset diagram illustrates the experimental setup for compressive testing. D) Stress curves for 1000 successive compression load-unload cycles at 30 % strain for PSCH@SBMA and PCH@EG. E) Tensile stress-strain curves of IPNCH@CAPAM and PSCH@SBMA. The inset diagram represents the tensile testing setup. F) Stress curves for 1000 successive tension load-unload cycles at 30 % strain for IPNCH@CAPAM. G) Schematic illustration of the interaction between IPNCH@CAPAM and PSCH@SBMA hydrogels and biological tissues. H) Peel-off strength measurements of IPNCH@CAPAM and PSCH@SBMA on pig skin, indicating their adhesive performance. I) The peel-off strength of IPNCH@CAPAM on the pig skin repeated 15 times.

The mechanical properties of the hydrogels are intrinsically linked to their microstructural architectures, which vary according to the three design strategies. The IPNCH@CAPAM hydrogel was prepared through an interpenetrating polymer network (IPN) strategy, which resulted in one of the softest hydrogels. This ultra-soft material exhibited an elastic modulus of approximately 280 Pa, which is well within the range of super-soft neural tissues (100–1500 Pa) [73]. The inhibition of PAM chain entanglement by the catechol groups of PDA effectively reduced the modulus. As shown in Fig. 4A, this ultra-soft property renders IPNCH@CAPAM highly suitable for implantable bioelectronics, especially in soft neural interfaces. Swelling reduced its modulus further, and this change has underscored the importance of understanding how water absorption affects the overall properties of hydrogels in practical applications (Fig. S20–21). In addition to its softness, IPNCH@CAPAM demonstrated excellent self-recovery behaviour. Under cyclic shear strain tests, the hydrogel quickly restored its original modulus after large strain deformations. When subjected to a strain of 300 % followed by 0.5 %, the hydrogel recovered to its initial modulus within 30 s (Fig. 4B, Fig. S22). This rapid gel-sol transition highlights its potential for dynamic applications requiring reversible deformation.

In compression tests, IPNCH@CAPAM withstood strains of up to 80 %, maintaining a compressive stress of 100.12 kPa under 80 % strain (Fig. 4C). Its fatigue resistance in compression was further confirmed through cyclic tests, where the hydrogel showed minimal stress relaxation and maintained its original strength throughout 100 cycles of loading-unloading at 20 % strain (Fig. S23A). This robust performance suggests that IPNCH@CAPAM is suitable for stable, long-term bioelectronic applications, minimizing signal baseline drift during extended use. In tensile tests, IPNCH@CAPAM exhibited impressive elasticity, achieving an elongation at break of 530 % and a tensile strength of 8.02 kPa (Fig. 4E). For 1000 tensile tests of IPNCH@CAPAM system, the stress remained relatively stable during the whole cycle (Fig. 4F, Fig. S23B–D). To fully assess its fatigue resistance and obtain critical parameters in the presence of microcracks, fatigue crack extension tests are inevitable [[74], [75], [76]]. A 3000-cycle fatigue test at 30 % strain further confirmed its long-term stability (Fig. S24). This combination of flexibility and strength makes the hydrogel well-suited for flexible electronic devices requiring high deformation tolerance, such as implantable bioelectronic devices.

The catechol-based redox-active system of IPNCH@CAPAM could enable covalent bonding and non-covalent interactions with various surfaces (Fig. 4G) [77]. Therefore, the adhesive properties of IPNCH@CAPAM were evaluated through 90° peel-off test on pig skin and lap shear tests for different substrates (Fig. 4H, Fig. S26). The hydrogel showed excellent adhesion properties, ensuring stable adhesion and conformal contact with various polar and nonpolar substrates. Even after 15 consecutive peel-off cycles on pig skin, IPNCH@CAPAM maintained more than 50 % of its initial adhesive strength (Fig. 4I), highlighting its robust, repeatable, and durable adhesion capabilities. This characteristic is essential for bioelectronic devices, effectively minimizing motion artifacts and ensuring stable signal acquisition during prolonged use.

PSCH@SBMA, prepared using a zwitterionic modification strategy, also showed favorable mechanical properties but with a slightly higher modulus compared to IPNCH@CAPAM. The elastic modulus of PSCH@SBMA was below 500 Pa, which is suitable for soft tissue applications but slightly stiffer than neural tissues. Its modulus, combined with its flexibility, makes it appropriate for both implantable and wearable bioelectronics (Fig. 4A). Similar to IPNCH@CAPAM, PSCH@SBMA exhibited excellent self-recovery properties during cyclic shear strain testing, responding rapidly to changes in strain (Fig. S22A).

In compression tests, PSCH@SBMA demonstrated a compressive stress of 1161.75 kPa under 82 % strain, showing higher compressive strength than IPNCH@CAPAM (Fig. 4C). This moderate compressive strength, coupled with its ability to maintain structural integrity after repeated compression suggests its suitability for applications that experience moderate mechanical stresses (Fig. 4D, Fig. S25). Tensile testing of PSCH@SBMA revealed an impressive elongation at a break of 789 %, with a tensile strength of 5.85 kPa (Fig. 4E). The favorable balance of extensibility and mechanical strength in PSCH@SBMA highlights its potential for use in wearable bioelectronics, where high flexibility and close conformability to the skin are essential.

In terms of adhesion, PSCH@SBMA also exhibited moderate bio-adhesive properties. The zwitterionic moieties facilitated strong interactions with biological surfaces through hydrogen bonding, electrostatic attraction, and dipole-dipole interactions (Fig. 4G–H) [78,79]. These adhesive properties make it suitable for applications where close and stable contact with biological tissues is required for reliable signal detection.

PCH@EG, the pure conducting polymer hydrogel without matrix support, displayed the highest elastic modulus among the three hydrogels at approximately 15 kPa, closely matching the modulus of human skin (∼20 kPa). This makes PCH@EG highly suitable for epidermal bioelectronics, where mechanical compatibility with the skin is essential (Fig. 4A, Fig. S22B). Despite its higher modulus, PCH@EG demonstrated strong compressive properties. It withstood strains up to 81 %, with a compressive stress of 1689.11 kPa, far exceeding that of IPNCH@CAPAM and PSCH@SBMA (Fig. 4C). Moreover, PCH@EG exhibited a stress reduction of 16.9 % after 1000 compression cycles (Fig. 4D, Fig. S25). This indicates that EG enhances the flexibility and conductivity of PEDOT. The early stress drop may be attributed to weak initial interactions between PEDOT and EG, leading to molecular rearrangement. However, as EG improves flexibility, the system demonstrates better stability in later cycles. Energy dissipation in the PCH@EG system also gradually decreases, with a 12.7 % reduction, eventually stabilizing. This suggests that the internal molecular chains reach equilibrium after initial rearrangement, reducing energy dissipation.

Overall, each of the three hydrogels exhibited distinct and stable mechanical properties tailored to different bioelectronic applications. The swelling of hydrogels also significantly affected their mechanical properties (Fig. S27–S29). In our experiments, we observed a substantial reduction in the shear modulus of the hydrogel after swelling, indicating a decrease of several orders of magnitude due to the softening of the hydrogel's network structure. This reduction in mechanical strength is critical, as it could impact the hydrogel's ability to maintain stress and strain under operational conditions. Considering that the typical deformation of the skin in daily life is less than 30 %, the elasticity of the hydrogels is deemed sufficient to meet the application requirements of wearable or implantable bioelectronic devices [80,81]. These comprehensive mechanical characterizations provide a solid foundation for their application in recording electrical and biopotential signals.

### 3.4 Electrical performance of the conducting polymer hydrogels

The electrical performance of the conducting polymer hydrogels is critical for enabling effective communication between tissues and bioelectronic devices. To assess this, the IPNCH@CAPAM, PSCH@SBMA, and PCH@EG hydrogels were analysed for their conductivity, impedance, and electrochemical stability, with a commercial gel electrode (CGE) serving as a reference.

**Fig. 5:**
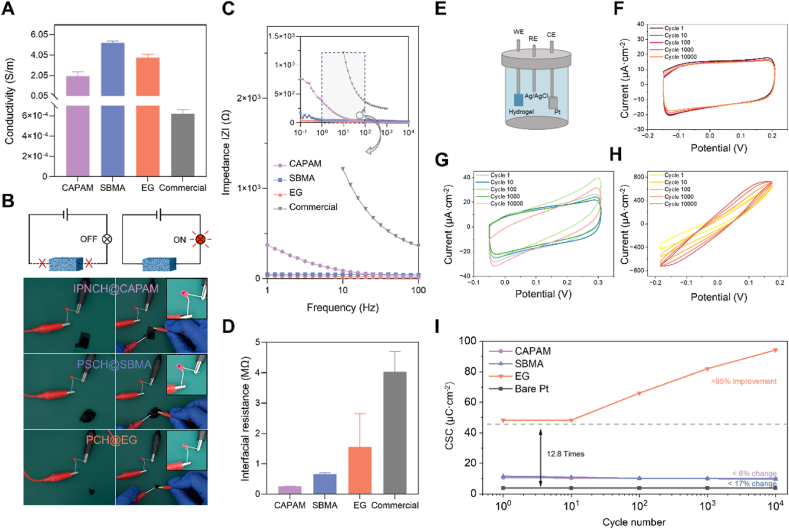
Electrical properties of conducting polymer hydrogels and comparison with commercial gel electrode. A) Electrical conductivity measurements of the three selected hydrogels (CAPAM, SBMA, EG) compared to a commercial gel electrode (CGE). B) Schematic of a device in which a hydrogel acts as a part conductor in a closed circuit to light an LED, and optical images of the corresponding three hydrogel patches. C) Electrical impedance spectroscopy of the hydrogels fully swollen in PBS over a physiologically relevant frequency range of 1–100 Hz, and the inset is Impedance spectroscopy over a larger frequency range (0.1–10^4^ Hz). D) Quantification of interfacial resistance of hydrogels and commercial gel electrode on the skin surface. E) Schematic of the three-electrode setup used in the CV measurements, consisting of Ag/AgCl as the reference electrode (RE), platinum (Pt) as the counter electrode (CE), the hydrogel under investigation as the working electrode (WE), and 0.1M Na_2_SO_4_ as the electrolyte. F) CV curves over 10,000 cycles of IPNCH@CAPAM.,. G) CV curves over 10,000 cycles of PSCH@SBMA. H) CV curves over 10,000 cycles of PCH@EG. I) Quantification of charge storage capacity (CSC) across 10,000 cycles, illustrating the capacity retention of the hydrogels over prolonged cycling.

IPNCH@CAPAM demonstrated excellent electrical properties, with a conductivity of 1.99 S/m, which was superior to the CGE (Fig. 5A). This high conductivity is attributed to the interpenetrating network of conducting polymers that ensures uniform pathways for electron transport. To visually demonstrate the electrical performance, Fig. 5B shows an LED circuit powered by a 3 V battery connected to IPNCH@CAPAM, which successfully illuminated the LED, indicating its effective conductivity.

In terms of impedance, the three proposed hydrogels showed a significant reduction compared to the commercial one, indicating improved conductivity and lower resistive losses at bio-relevant frequencies (Fig. 5C, Fig. S30). This is critical for minimizing resistive losses and ensuring stable electrical signal transmission across bioelectronic interfaces. The interfacial resistance of IPNCH@CAPAM on human skin was also lower than that of the CGE, confirming its superior ability to form a low-resistance interface with biological tissues (Fig. 5D). The electrochemical properties of hydrogels are also crucial for their bioelectronic stimulation capability for further applications [[82], [83], [84], [85]]. Therefore, the electrochemical performance of IPNCH@CAPAM was evaluated over 10,000 cycles using cyclic voltammetry (CV), where it exhibited minimal reduction in charge storage capacity (CSC), maintaining nearly 95 % of its original CSC (Fig. 5E–F, 5I). This highlights the hydrogel's remarkable electrochemical stability, making it a highly promising candidate for long-term bioelectronic applications, particularly for devices requiring sustained electrical stimulation.

Next, PSCH@SBMA was evaluated, demonstrating the highest electrical conductivity among the three hydrogels at 5.25 S/m (Fig. 5A). Similar to IPNCH@CAPAM, PSCH@SBMA also successfully lit an LED in a closed-circuit test (Fig. 5B), showcasing its effective electron transport capabilities. In terms of impedance, PSCH@SBMA exhibited significant reductions across the physiological frequency range, comparable to IPNCH@CAPAM (Fig. 5C). This reduction in impedance is crucial for improving signal transmission efficiency in bioelectronics applications. When tested for interfacial resistance, PSCH@SBMA also outperformed the CGE, indicating its ability to form a stable, low-resistance interface with biological tissues (Fig. 5D).

The electrochemical performance of PSCH@SBMA, as evaluated by CV, also demonstrated excellent stability. Over 10,000 cycles, it displayed a high CSC (11.49 μC cm^−2^) with only a 16.6 % decrease, highlighting its electrochemical durability for long-term use (Fig. 5G and I). Further insights were provided by BET analysis, which revealed a significant increase in the specific surface area of the hydrogel—from 5.735 m^2^/g to 15.044 m^2^/g—after 50 days of water immersion (Table S3). This substantial growth suggests progressive structural swelling and reorganization within the hydrogel network over time. The swelling behaviour further contributes to enhanced ionic mobility by forming more continuous conductive pathways, which is consistent with the observed increase in CSC after prolonged soaking (Fig. S32). This stability ensures that PSCH@SBMA is well-suited for bioelectronic devices that require consistent electrical stimulation, such as neural interfaces and cardiac pacemakers.

Finally, the PCH@EG hydrogel was analysed. Its conductivity reached 3.79 S/m, which, while lower than PSCH@SBMA, was still a significant improvement over the CGE (Fig. 5A), and could be applied to systems such as skin electrodes or sensors requiring fast electrical signal response. The hydrogel effectively powered the LED circuit in Fig. 5B, further confirming its conductive properties. In terms of impedance, PCH@EG showed improvements over the CGE, though not as pronounced as the other two hydrogels (Fig. 5C). Its interfacial resistance was also lower than that of the CGE, ensuring effective electrical contact with biological tissues (Fig. 5D).

Moreover, PCH@EG exhibited a significantly high CSC of 48.23 μC cm^−2^, which surpasses that of a bare Pt electrode (3.76 μC cm^−2^) of identical size by a factor of 12.8 (Fig. 5H and I). Notably, the CSC of PCH@EG increases with the number of CV cycles, achieving a remarkable improvement of over 95 % after 10,000 cycles. The enhancement of electrochemical performance can be attributed to the structure of the pure PEDOT:PSS hydrogel, which favors the full exposure of the conductive polymer in the electrolyte, allowing the PEDOT chains to rearrange more freely under the influence of an electric field during CV cycling. This molecular rearrangement leads to greater order in the PEDOT segments and improved continuity of the conductive channels, resulting in a significant boost to CSC even after extensive cycling. This behavior suggests that the PCH@EG hydrogel maintains excellent electrochemical stability and sustained charge storage capacity under prolonged electrochemical activation, qualifying it as a promising material for applications in flexible bioelectronic devices, including electrophysiological signal recording and skin-based electrical stimulation.

The three hydrogels were also immersed in different body fluids such as simulated body fluid, simulated sweat, and simulated cerebrospinal fluid, and their electrochemical stability was tested and verified under 10,000 cycles and even 100,000 cycles of circulation (Fig. S33–S36). These results from the electrical performance tests underscored that the three conducting polymer hydrogels exhibited excellent electrical conductivity, low interface resistance, and remarkable stability (Fig. S31). These findings indicate that the three hydrogels, with their robust electrical performance, could enable more reliable and efficient bioelectronic devices, particularly for applications in tissue-electronics interfaces, where long-term stability and low interfacial resistance are crucial.

From the perspective of the mechanical and electrical properties of the hydrogels constructed by the three strategies, these three strategies form a hierarchical framework property optimization: Firstly, IPNCH functions as the foundation, validating that mechanical compliance can be drastically improved by decoupling conductive and structural networks; then, PSCH introduces ionic additives to refine conductive pathways, demonstrating that controlled microphase segregation enables simultaneous enhancement of stretchability and conductivity; finally, PCH simplifies the system by eliminating non-conductive matrices, proving that solvent-mediated crystallization alone can achieve skin-compatible properties. Crucially, insights from each strategy (e.g., polarity effects in PCH informed ionic dopant selection in PSCH) enabled cross-pollination, advancing the unified goal of mechanical-electrical harmony.

### 3.5 Epidermal electrophysiological signal recording

The exceptional mechanical and electrical properties of the conducting polymer hydrogels facilitated their application as bioelectrodes for detecting epidermal electrophysiological signals, including electromyography (EMG), electrocardiography (ECG), and electroencephalography (EEG) (Table S5). On the basis of ensuring biocompatibility, we applied three kinds of hydrogels to record electrophysiological signals (Fig. S38–S39). Freshly prepared hydrogels that are not completely swollen to ensure signal stability, mechanical strength, and optimal contact were utilized between the bioelectrode and tissue. This approach is crucial, as fully swollen hydrogels exhibit reduced adhesion and mechanical properties, which can compromise their effectiveness in clinical applications.

**Fig. 6:**
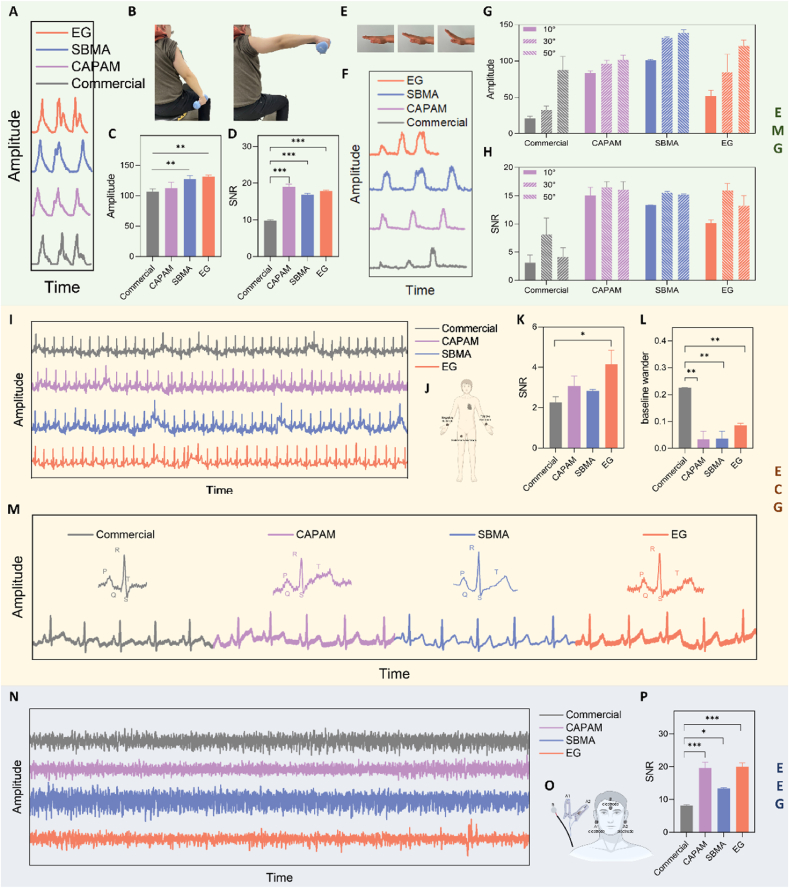
Epidermal electrophysiological signals recorded using hydrogel bioelectrodes. A) Electromyogram of lateral dumbbell lifting process recorded by hydrogel electrodes. B) The EMG signal was recorded during side lifting of the dumbbell. C) Amplitude of EMG signals recorded by hydrogels and commercial gel electrode during side-lifting dumbbells. D) SNR_EMG_ recorded by hydrogel and commercial gel electrodes during side-lifting dumbbells. E) EMG generated by the motion of raising the palm at different angles was recorded. F) EMG produced by the motion of raising the palm at increasing angles. G) Amplitude of EMG signals recorded by hydrogels and commercial gel electrode during raising the palm at increasing angles. H) SNR_EMG_ recorded by hydrogel and commercial gel electrode during raising the palm at increasing angles. I) Epidermal ECG signals recorded by hydrogels and commercial gel electrodes. J) Schematic illustration of recording epidermal ECG signals in resting state. K) SNR_ECG_ recorded by hydrogels and commercial gel electrode. L) Baseline wander of epidermal ECG recorded by hydrogel and commercial gel electrodes. M) Enlarged ECG characteristic signal diagram recorded by hydrogel and commercial gel electrode. N) Epidermal EEG signals recorded by hydrogels and commercial gel electrodes. O) Schematic illustration of recording epidermal EEG signals in resting state. P) SNR_EEG_ recorded by hydrogels and commercial gel electrode.

Initially, hydrogel electrode pads were attached to the flexor muscle (right arm) of a volunteer. The volunteer performed lateral lifts of a 2 kg dumbbell, followed by lowering the arm with an interval of 3 s for 2 s (Fig. 6A and B). All hydrogels recorded significant EMG signals during bicep contractions, achieving higher signal amplitude and signal-to-noise ratio (SNR_EMG_) compared to the commercial gel electrode (Fig. 6C and D). Furthermore, performing gestures with smaller motion amplitudes successfully revealed characteristic action potential signals corresponding to the motion in the electromyogram (Fig. 6E). Hydrogels were attached to the volunteer's flexors (outer forearms) and elbows to form electrodes. As the palm was gradually lifted upward, the degree of flexor fiber contraction increased gradually. As shown in Fig. 6F, akin to the commercial gel electrode, the hydrogel electrodes captured EMG signals of increasing amplitude at higher palm lift angles (10°, 30°, 50°). Muscle contraction induced noticeable changes in the potential intensity, highlighting the remarkable sensitivity of the hydrogel electrodes in discerning the varying forces resulting from different palm lift heights. In addition, the average amplitude and SNR_EMG_ collected by the three hydrogels exceeded those of CGE at any hand lift angle (Fig. 6G and H, and Fig. S40). At the highest palm lift angle, IPNCH@CAPAM yielded an SNR_EMG_ of 16.4 dB, more than double the SNR_EMG_ recorded by CGE (8.1 dB). The results were attributed to the soft properties of the hydrogel itself resulting in conformal contact between the hydrogel electrodes and the tissues, and the excellent electrical properties further ensured that the electrodes obtained stable and high-quality signals. Therefore, the stable and effective recording of epidermal EMG signals preliminarily proved the potential of the conducting polymer hydrogels constructed by three modification strategies for the application of bioelectronics.

Hydrogel electrodes were further utilized to detect epidermal ECG signals (Fig. 6I, Fig. S41–S43). ECG is employed as an effective tool for monitoring the heart's electrical activity, providing critical insights into cardiac abnormalities such as arrhythmias, impaired coronary blood flow, and electrolyte imbalances [5,86]. Three hydrogel electrode pads were attached to the left/right arms and left ankle of a volunteer to record the ECG, as shown in Fig. 6J. The hydrogel electrodes successfully captured stable ECG signals akin to the commercial gel electrode, displaying typical ECG waveforms, including the P wave (atrial depolarization), QRS complex (ventricular depolarization), and T wave (ventricular repolarization) (Fig. 6M). The comparison results of electrocardiogram signals indicated that the hydrogel electrodes exhibited a higher SNR_ECG_, and PCH@EG demonstrated significant advantages (Fig. 6K). Furthermore, the sliding window average filter in MATLAB was employed to correct the baseline drift of the collected ECG signals, and the baseline wander was calculated (Fig. 6L). It was evident that the baseline wander of the three hydrogel electrodes was substantially smaller than that of the CGE, with IPNCH@CAPAM exhibiting a baseline wander of only about 15 % compared to the CGE. The stable recording of ECG and the clear recognition of their waveforms demonstrated the potential significance of hydrogel electrodes in health monitoring and disease diagnosis, including conditions such as myocarditis and arrhythmia. Looking ahead, programmable 3D printing and microfabrication techniques offer promising routes to pattern hydrogel materials into conformal multi-electrode arrays or multilayer, physiologically scaled structures [[87], [88], [89], [90], [91], [92]]. These advanced fabrication strategies would enable high-resolution spatial mapping, paving the way for more precise and sophisticated bioelectronic applications.

The monitoring of epidermal EEG signals further exemplified the utility of hydrogel-based bioelectrodes. Hydrogels were employed as bioelectrodes on the forehead and two earlobes of the volunteer to capture EEG signals at rest (Fig. 6O). During the subject's resting state, the EEG signals obtained from the hydrogels and commercial gel electrode presented similar patterns. Notably, the signals from the hydrogel electrodes demonstrated optimal stability without any artifacts (Fig. 6N, Fig. S44). Moreover, the signal-to-noise ratio of the EEG signals (SNR_EEG_) also demonstrated that the hydrogel electrodes yielded higher-quality EEG signals (Fig. 6P). Characteristic alpha activity at approximately 10 Hz was identified through Fast Fourier transformation of the three electrical derivations during the resting state reordered in 30 s, displaying specific resting activity within the frequency range of 8–13 Hz (Fig. S45). Interestingly, IPNCH@CAPAM and PCH@EG hydrogel electrodes exhibited an even higher proportion of the alpha band, indicating genuine signals rather than artifacts. The results underscored that the hydrogel electrode pads developed in this paper could reliably capture high-quality epidermal electrophysiological signals. The hydrogel-integrated bioelectrodes showed superior performances compared to the commercial gel electrode because of their softness, excellent conductivity, and robust fit, rendering them ideal materials for application in epidermal bioelectronics.

### 3.6 Implantable electrophysiological signal recording

**Fig. 7:**
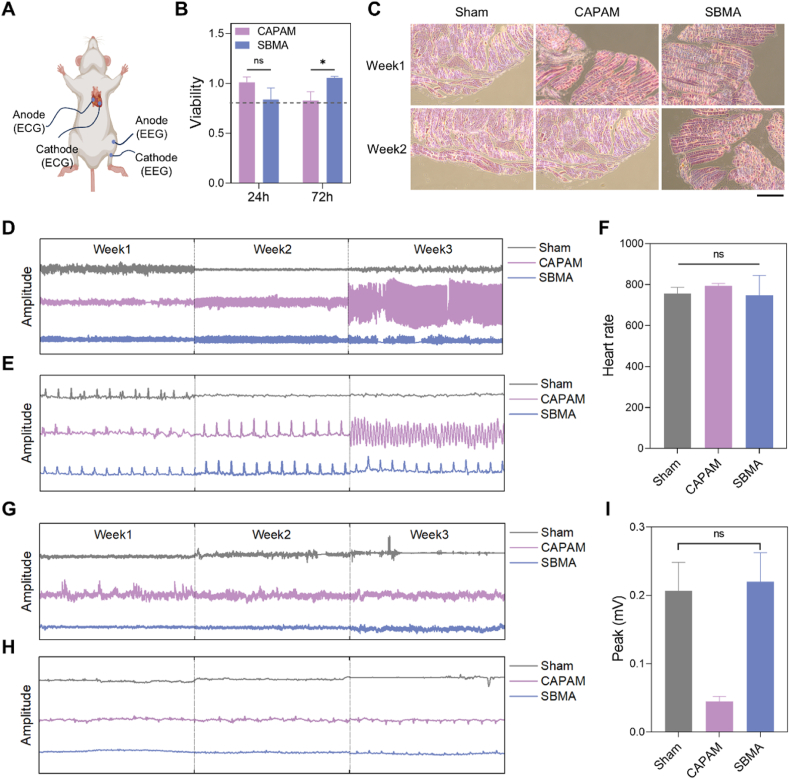
Implantable electrophysiological signals recorded using hydrogel bioelectrodes. A) Schematic illustration of hydrogels as bioelectrodes to connect HD-X02 implant to biological tissues for implantable recording of ECG and EMG signals. B) Cell viability of BHK21 cells cultured in hydrogel extraction medium for 24 h and 72 h. Data are mean ± SD (n = 4). ∗p < 0.05 (one-way ANOVA with Tukey's test). The dashed line indicates the 80 % biocompatibility threshold. C) Representative H&E staining images of tissues of mouse tissues throughout 2 weeks in subcutaneous hydrogel implantation group and non-hydrogel implantation sham group. There was no significant difference between the experimental groups and the sham operation group, as the cells were intact and arranged neatly, and no obvious inflammatory cell aggregation was observed. Scale bar: 50 μm. D-E) Implantable ECG signals recorded by hydrogel bioelectrodes and non-hydrogel control group for 3 consecutive weeks (continuous signals within 30 s and amplified signals within 1 s, respectively). F) Heart rate detected by hydrogel bioelectrodes and non-hydrogel control group. G-H) Implantable EMG signals recorded by hydrogel bioelectrodes and non-hydrogel control group for 3 consecutive weeks (continuous signals within 30 s and amplified signals within 1 s, respectively). I) EMG peaks detected by hydrogel bioelectrodes and non-hydrogel control group.

Besides wearable devices attached to the skin, the exceptional softness and excellent biocompatibility of hydrogels position them as promising candidates for implantable bioelectronics. Biocompatibility is a pivotal factor in implantable bioelectronics, ensuring the accurate capture of physiological data and mitigating foreign body responses during long-term applications (Fig. 7A) [[93], [94], [95]]. Due to its weak modulus and toughness, PCH@EG was not deemed suitable for the application of implantable bioelectronic devices. Consequently, IPNCH@CAPAM and PSCH@SBMA underwent testing for *in vitro* cytotoxicity and *in vivo* biocompatibility before potential application. *In vitro* cytotoxicity assessment involved measuring the viability of seeded Baby Hamster Syrian Kidney (BHK21) cells and other human-derived cells incubated in leaching solutions. As shown in Fig. 7B and Fig. S38, the cell viabilities remained above 80 % after 24 and 72 h of culture, meeting the biocompatibility standard. Among them, PSCH@SBMA performed better due to the optimization of residual monomer elimination. The results demonstrated that the hydrogels had negligible cytotoxicity to the BHK21 cells. To further investigate biocompatibility, the hydrogels were implanted in mice dorsal subcutaneous for 2 weeks, and histological analysis was conducted to test the immune response of *in vivo* tissues toward the hydrogels (Fig. S46–S49). Fig. 7C showed the representative histological cross-sectional views of the mice subcutaneous tissues that were stained with hematoxylin-eosin. No inflammatory reaction was observed in the tissues around the hydrogels and there was no obvious difference in tissue morphology between the tested tissue and normal tissue. In addition, to ensure stable connections between the hydrogels and biological tissues *in vivo*, we investigated the friction coefficients of the hydrogels through repeated friction tests conducted in a normal saline environment. The data indicated that IPNCH@CAPAM and PSCH@SBMA could maintain stable contact with the tissue, as evidenced by the friction coefficient measurements (Fig. S37). IPNCH@CAPAM showed strong mechanical properties and a stable coefficient of friction particularly, making it ideal for implantable electrode materials that require high friction and wear resistance. It could maintain structural integrity and performance stability in the case of changes in external mechanical stress and internal environmental conditions. All the results collectively affirmed that the IPNCH@CAPAM and PSCH@SBMA displayed negligible cytotoxicity, excellent biocompatibility, and adequate frictional resistance, positioning them as promising candidates for use in implantable bioelectronics.

To evaluate the *in vivo* effectiveness of the hydrogel bioelectrodes, they were initially integrated with the ultra-small animal specific HD-X02 implant, which incorporated PhysioTel™ technology developed by Data Sciences International (DSI), for implantable ECG acquisition and monitoring (Fig. S50). It has been reported that the implantable recorded ECG shows sharper P wave and larger QRS complex amplitude over those obtained by epidermal ECG [96]. Implantable recorded ECG exhibits more obvious virtues in detecting cardiac allograft rejection, diagnosing sinus rhythm, and electrocardiographic imaging [[96], [97], [98]]. The bio-potential leads of the HD-X02 implant, designed for electrical signal acquisition, were embedded into the hydrogel precursor solution and co-formed with the hydrogel film in a mold to thin film bio-electrodes measuring 1 cm × 1 cm × 1 mm. Hydrogel electrodes coated ECG biopotential leads were secured in a lead 2 configuration to the costal periosteum with nonabsorbable braided polyester sutures to prevent shedding. A control group utilized leads directly as electrodes. The implant was activated once a week for 30 min to record ECG signals over three weeks. The heart rate of mice detected by hydrogel electrodes and biopotential leads was approximately 750 bpm, with no significant difference between the data obtained by hydrogel electrodes and control group (Fig. 7F). Fig. 7D showed the ECG signals for 1 min during each test. From the second week onwards, the non-hydrogel electrode control group showed a notable reduction in the amplitude of the detected signals. Conversely, in the hydrogel groups, the instability/noise interference of monitoring signals gradually appeared till the third week. According to the amplified ECG signals in 1 s, PSCH@SBMA achieved continuous monitoring of ECG characteristic signals for three consecutive weeks (Fig. 7E). IPNCH@CAPAM successfully monitored ECG characteristic signals for the first two weeks, while the non-hydrogel group failed to obtain stable signals since the second week. The results suggested that the hydrogel thin film bioelectrodes promoted stable contact between bioelectronics and the tissues, unlike original biopotential leads, which struggled to establish firm connections and might experience frictional slippage within the organism.

EMG with lower signal amplitude was further used as another benchmark to characterize the performance of hydrogel bioelectrodes in implantable electrophysiological signal monitoring. Similarly, the non-hydrogel electrode control group began to show intermittent signal by week 3, whereas the IPNCH@CAPAM and PSCH@SBMA hydrogel film bioelectrodes maintained stable signal recording for the entire three-week duration (Fig. 7G and H). Comparing the EMG peak values, no significant difference was observed between PSCH@SBMA and the control group, indicating comparable signal strength. However, IPNCH@CAPAM exhibited a slightly lower peak value. This variance might be attributed to the relatively lower conductivity of IPNCH@CAPAM and potential signal loss during transmission (Fig. 7I). These results demonstrated the effectiveness of hydrogel films, with their synergistic mechanical and electrical properties, in establishing robust connections between bioelectronic devices and tissues as implantable bioelectrodes. This ensured the consistent acquisition of reliable and high-quality electrophysiological signals over extended periods.

## 4 Conclusions

In this work, a modular microstructure‐engineering framework that addresses the longstanding trade‐off between mechanical compliance and electrical performance in conducting polymer hydrogels was presented. Central to all strategies is the incorporation of PEDOT:PSS as the primary conductive component, forming continuous electronic pathways within the hydrogel matrix. By sequentially implementing three complementary strategies—interpenetrating network conductive hydrogels (IPNCHs) strategy, ion-induced phase separation conductive hydrogels (PSCHs) strategy, and polar solvent–assisted pure conductive hydrogels (PCHs) strategy— chain entanglement, domain architecture, and crystallinity could be rationally tuned to span a wide mechanical‐electrical spectrum. These strategies are interconnected within a unified design philosophy, together establishing a cohesive materials engineering framework for bioelectronic applications.

Each strategy selectively enhances key hydrogel properties, providing tailored solutions for different mechanical-electrical performance demands. Specifically, IPNCH@CAPAM achieves an ultra‐low modulus of 0.28 kPa with 530 % elongation and 1.99 S/m conductivity; PSCH@SBMA balances 0.44 kPa modulus with 789 % stretchability and 5.25 S/m conductivity; PCH@EG reaches skin‐matched stiffness (15 kPa) and 3.79 S/m conductivity under a simple solvent‐annealing route. Together, they cover applications from soft neural interfaces to high‐fidelity epidermal sensors. In demonstration, PCH@EG electrodes record EMG/ECG/EEG with SNR up to 20 dB, while IPNCH@CAPAM and PSCH@SBMA sustain stable implantable ECG/EMG readings for over three weeks.

By integrating multidimensional property screening alongside systematic *in vitro* and *in vivo* validations, the study not only bridges isolated “performance islands” within the field of conducting hydrogels, but also establishes a blueprint for next‐generation bioelectronic materials. Looking ahead, further combining these strategies in hierarchical hybrids, incorporating self‐healing motifs, 3D-printed microelectrode arrays, and scaling up production will further expand their utility in personalized, wearable, and implantable devices.

Ethics approval and consent to participate

Ethics approval and consent to participate The animal experiments were performed in accordance with the relevant rules and regulations, and were reviewed and approved by the Institutional Animal Care and Use Committee (IACUC) of Tsinghua University (approval number: 24-LY2, approval date: 2024-1-18).

## CRediT authorship contribution statement

**Ting Wang:** Writing – review & editing, Writing – original draft, Validation, Methodology, Investigation, Formal analysis, Data curation. **Jiajun Liu:** Investigation, Data curation. **Yuli Zhao:** Investigation, Data curation. **Yuan Lu:** Writing – review & editing, Supervision, Investigation, Funding acquisition, Conceptualization.

## Declaration of competing interest

The authors declare that they have no known competing financial interests or personal relationships that could have appeared to influence the work reported in this paper.
