# Supplementary material for: Synergistic mastery: Advancing mechanical and electrical harmony in conducting polymer hydrogel bioelectronics
Source: Bioact Mater. 2025 Jun 11;52:300–17. doi: 10.1016/j.bioactmat.2025.06.015 (PMC12197989; doi:10.1016/j.bioactmat.2025.06.015)
Supplement: Multimedia component 1 [file mmc1.docx]

Supporting Information

Synergistic Mastery: Advancing Mechanical and Electrical Harmony in Conducting Polymer Hydrogel Bioelectronics

Legend and contents of supplementary information:

Supporting Methods

Figure S1. Impedance comparison and screening of IPNCH, PSCH and PCH in different formulations.

Figure S2. Comparison of conductivity, modulus and impedance of IPNCH@CAPAM within different PEDOT:PSS content.

Figure S3. Optical images of IPNCH@CAPAM within different PEDOT:PSS content.

Figure S4. Comparison of conductivity, modulus and impedance of PSCH@SBMA within different SBMA content.

Figure S5. Comparison of conductivity, modulus and impedance of PCH@EG within different EG content.

Figure S6. The properties of varying raw material ratios IPNCH@CAPAM, PSCH@SBMA, PCH@EG were compared and screened.

Figure S7. Characterization of gelling time for IPNCH@CAPAM and PCH@EG.

Figure S8. The X-Ray diffraction (XRD) pattern and the X-ray photoelectron spectroscopy (XPS) spectra of IPNCH@CAPAM.

Figure S9. The characterization of PSCH@SBMA with different SBMA content.

Figure S10. Nuclear magnetic resonance (NMR) spectroscopy of PCH@EG.

Figure S11. Characterization of PCH@EG as mixed solution group, before annealing, after annealing, and after soaking.

Figure S12. The removal ratios of polar solvents in PCH after annealing and soaking processes.

Figure S13. SEM images of IPNCH@CAPAM.

Figure S14. SEM images of PSCH@SBMA within different SBMA content.

Figure S15. SEM images of PSCH@SBMA.

Figure S16. SEM images of PCH@EG within different EG content.

Figure S17. SEM images of PCH@EG.

Figure S18. Morphology and phase diagrams of PSCH@SBMA within different SBMA content.

Figure S19. Morphology and phase diagrams of PCH@EG within different EG content.

Figure S20. Cross-linking efficiency of IPNCH@CAPAM, PSCH@SBMA, and PCH@EG.

Figure S21. Swelling properties of IPNCH@CAPAM, PSCH@SBMA, and PCH@EG.

Figure S22. Step strain scanning of conducting polymer hydrogels.

Figure S23. Repeated tensile/compression test for fatigue resistance of IPNCH@CAPAM.

Figure S24. Fatigue crack test of IPNCH@CAPAM.

Figure S25. Repeated compression test for fatigue resistance of PSCH@SBMA and PCH@EG.

Figure S26. Adhesion properties of conducting polymer hydrogels IPNCH@CAPAM.

Figure S27. The swelling rate measurements over time taken for IPNCH@CAPAM and PSCH@SBMA, and the corresponding modulus and conductivity.

Figure S28. Performance of 25 vol.% PCH@EG before and after immersion in DI water or artificial lysosomal fluid for three weeks.

Figure S29. Thermal and pH stability of hydrogels.

Figure S30. Impedance of commercial gels in the frequency range 10-1000Hz.

Figure S31. Effect of pH on the electrical properties of hydrogels.

Figure S32. Characterization of the time stability of the electrical properties of hydrogels by cyclic voltammetry.

Figure S33. Electrochemical properties of the selected hydrogels swollen in artificial perspiration fluid (aPF), artificial lymphatic fluid (aLF), and artificial cerebrospinal fluid (aCSF).

Figure S34. Characterization of the long-term stability of the electrochemistry properties of hydrogels by cyclic voltammetry.

Figure S35. The charge injection capacity of the selected hydrogels swollen in aPF, aLF, and aCSF.

Figure S36. Cyclic voltammetry curves of commercial gel and metal electrode for 2000 cycles.

Figure S37. Friction coefficient of IPNCH@CAPAM, PSCH@SBMA, and PCH@EG.

Figure S38. Cell viability of cells cultured in hydrogel extraction medium for 24h and 72h.

Figure S39. Optical photos of the rabbit skin irritation experiments with PCH@EG and PCH@DMSO.

Figure S40. Statistical significance analysis of EMG data generated by palm lifting at different angles.

Figure S41. Preprocessed epidermal ECG signals recorded by hydrogel electrodes and commercial gel electrode after noise reduction treatment.

Figure S42. Power spectral density of epidermal ECG signals recorded by hydrogel electrodes and commercial gel electrode.

Figure S43. Baseline drift of epidermal ECG signals recorded by hydrogel electrodes and commercial gel electrode.

Figure S44. Epidermal EEG signals recorded by hydrogel electrodes and commercial gel electrode.

Figure S45. Power spectral density of epidermal EEG signals recorded by hydrogel electrodes and commercial gel electrode.

Figure S46. Optical images of inflammation in mice implanted subcutaneous hydrogel IPNCH@CAPAM.

Figure S47. Optical images of inflammation in mice implanted subcutaneous hydrogel PSCH@SBMA.

Figure S48. Mass change ratio of hydrogels after one and two weeks of subcutaneous implantation in mice.

Figure S49. The immunohistochemical results of CD68 after subcutaneous implantation of hydrogels.

Figure S50. The surgical procedure for the ultra-small animal specific HD-X02 implant in mice.

Table S1. Screening of hydrogel formulations to identify compositions with high conductivity, low impedance, and low modulus.

Table S2. Performance comparison of finally selected hydrogel.

Table S3. BET Specific Surface Area Analysis of conducting polymer hydrogels.

Table S4. The average surface roughness of PSCH@SBMA and PCH@EG.

Table S5. Summary of preprocessing steps tailored to signal frequency characteristics and noise sources.

Table S6. Performance comparison between conducting hydrogels for electrophysiological signal recording.

Supplementary data processing program

Supporting Notes

**Supporting Methods**

1. **Preparation of SBMA@Water**

Since the self-polymerization of SBMA in water only proceeded at high monomer concentration, 50 wt.% of SBMA was mixed with deionized water in a vial and left to stand for at least 24 hours for dialysis treatment. After overnight dialysis, the solution inside the dialysis bag turned into a solid gel, and then the matrix part was freeze-dried for subsequent characterization tests.

1. **Measurement of friction coefficient**

The coefficient of friction (COF) of hydrogel was determined by a MCR 302 rotational rheometer (Anton Paar GmbH, Graz, Austria). First, various hydrogels were fixed on the bottom plate of the petri dish with 502 glue, and normal saline was added as a buffer. Friction test was carried out by using PP08 rotor (stainless steel 316L, roughness 0.1µm) in contact with hydrogel surface. A specified normal force is applied perpendicular to the hydrogel interface, and then a relative slip (strain 1%, frequency 1Hz) between the rotor and the hydrogel contact surface is induced by rotation of the rheometer. The friction force F is obtained by measuring the torque data. Then, divide the friction force by the normal force to determine the COF. The coefficient of friction (COF) was calculated using the following equation:

$$\begin{aligned} COF=\frac{F}{F_{N}}=\frac{4M}{3RF_{N}}\#\left( S1 \right) \end{aligned}$$

where $M$ is torque (N·m), $R$ is the rotor radius (m), and $F_{N}$ is the normal force (N).

1. **Material characterization**

Size distribution and zeta potential were measured using a Zetasizer Nano ZS (Malvern Panalytical Ltd., Malvern, UK). Thermogravimetric analysis coupled with mass spectrometry (TG–MS) was performed on a Q5000 IR thermogravimetric analyzer (TA Instruments, New Castle, DE, USA) under a nitrogen flow of 50 mL/min. Samples were heated from room temperature to 800 °C at a rate of 10 °C/min, and the evolved gases were monitored in real time using a quadrupole mass spectrometer with a mass-to-charge (m/z) range of 14–63 amu. Nuclear magnetic resonance (NMR) spectra were recorded on a JNM-ECZ400 NMR spectrometer (JEOL Ltd., Tokyo, Japan). Transmission electron microscopy (TEM) analyses were carried out using a JEM-2010 transmission electron microscope (JEOL Ltd., Tokyo, Japan). X-ray photoelectron spectroscopy (XPS) measurements were performed using a ESCALAB 250Xi X-ray photoelectron spectrometer (Thermo Fisher Scientific, Waltham, MA, USA).

1. **Quantitative residual amount of polar solvents**

Samples of 25 vol.% PCH@EG and 25 vol.% PCH@DMSO hydrogels (initial mass m = 500 mg) were subjected to a 24-hour extraction in extraction solvents at a ratio of 5 mL per sample. For EG (boiling point 197.3°C), deionized water was used as the extraction solvent, while acetonitrile was used for DMSO (boiling point 189°C). Quantitative extraction was performed at three different stages: before annealing, after three cycles of annealing at 130°C for 30 minutes, and after 24-hour treatment in deionized water.

The molar concentration 𝑛 of the polar solvents in the extraction solution was determined using liquid or gas chromatography. The mass fraction 𝜔 of residual solvents in the hydrogel was calculated using the equation:

$$\begin{aligned} \omega=\frac{n*M_{solvent}*V}{m}\#\left( S2 \right) \end{aligned}$$

where $M_{solvent}$ is the molar mass of the solvent, $V$ is the volume of the extraction solvent, and $m$ is the initial mass of the sample.

The removal ratio 𝑟 was calculated using the following equation:

$$\begin{aligned} r=\frac{\omega_{0}-\omega}{\omega_{0}}\#\left( S3 \right) \end{aligned}$$

where $\omega_{0}$ is the initial solvent mass fraction, $\omega$ is the residual mass fraction after treatment.

Liquid chromatography (LC) was performed on a ACQUITY UPLC system (Waters Corporation, Milford, MA, USA) for quantitative analysis of the EG content in the samples. The system was operated in negative electrospray ionization (ESI) mode. High-purity nitrogen served as the nebulizer, desolvation, and cone gas, while argon was used as the collision gas. Chromatographic separation was achieved by injecting a 10 μL sample onto a 150 × 2.1 mm HSS T3 C18 column (1.8 μm particle size; Waters Co.), maintained at 40 °C in the column oven. The LC system was operated in gradient mode at a flow rate of 300 μL/min using Solvent A (0.1% formic acid in water, 26.5 mmol/L) and Solvent B (methanol containing 0.1% formic acid). The gradient program was as follows: a linear increase from 2% to 100% Solvent B over 0–2.70 min, a hold at 100% B for 0.30 min, a decrease back to 2% B over 0.1 min, and an equilibration at 2% B for 1.10 min, resulting in a total run time of 4.5 min per injection.

Gas chromatography (GC) was performed on a GC-2014 gas chromatograph (Shimadzu Corporation, Kyoto, Japan) using a DB-FFAP column (Agilent, J&W Scientific; 30 m × 0.325 mm × 0.5 μm) for quantitative analysis of the DMSO content in the samples. The injection port was maintained at 250 °C with a split ratio of 20:1, using high-purity helium as the carrier gas at a flow rate of 1.5 mL/min. The temperature program was as follows: 40 °C (held for 2 min), ramped at 15 °C/min to 240 °C (held for 5 min). The flame ionization detector (FID) was set at 280 °C, with hydrogen and air flows of 40 mL/min and 400 mL/min, respectively. The injection volume was 1 μL.

1. **Wide-Angle X-Ray Scattering (WAXS)**

The X-ray scattering experiments were conducted in transmission geometry at room temperature using a PILATUS3 3×100K detector. An X-ray beam with a wavelength of 0.15 nm was employed, and the sample-to-detector distance was set to 0.08 m. The detector was configured with a pixel size of 0.2 mm in both dimensions, and the X-ray beam was directed perpendicularly onto the sample. Each measurement was performed with an exposure time of 600 s, and the resulting two-dimensional scattering data were processed using an azimuthal averaging method to yield the scattered intensity, I(q), as a function of the scattering vector, q (where q = 4π sinθ/λ, with θ being the half scattering angle). Calibration was carried out using a silver behenate standard, and the effective q-range for the measurements was determined to be approximately 0.3 nm⁻¹ < q < 4.2 nm⁻¹.

1. **charge injection capacity (CIC) measurements**

CIC measurements were performed by applying bidirectional injection pulses with a 1 ms duration across a potential range from −0.5 to +0.5 V for 100,000 cycles. The charge injection capacity (CIC) of the bioelectronic interface on the platinum electrode was calculated by the following formula:

$$\begin{aligned} CIC=\frac{Q_{inj\left( c \right)}}{A}+\frac{Q_{inj\left( a \right)}}{A}\#\left( S4 \right) \end{aligned}$$

where $Q_{inj\left( c \right)}$ and $Q_{inj\left( a \right)}$ represent the total cyclic capacitive charge injected/released after cathodic and anodic potential sweeps, respectively, and $A$ is the area of the hydrogel coating on the bare platinum electrode.

1. **Lap Shear Adhesion**

Adhesive joints were prepared by bonding substrates (10 × 100 × 1.5 mm³) with an overlap area of 10 × 10 mm². Specimens were tested on a WDW-3020 universal testing machine (Changchun Kexin Test Instrument Co., Ltd., Changchun, China) at a crosshead speed of 5 mm/min. The maximum load at failure was recorded, and shear strength $\tau$ was calculated as:

$$\begin{aligned} \tau=\frac{F}{A}\#\left( S5 \right) \end{aligned}$$

where $F$ is the failure load and $A$ is the overlap area.

1. **Rabbit Skin Irritation**

The animal experiments were performed in accordance with the relevant rules and regulations, and were reviewed and approved by the Institutional Animal Care and Use Committee (IACUC) of Tsinghua University (approval number: 24-LY2, approval date: 2024-1-18). For the skin irritation tests, the dorsal skin of New Zealand White rabbits (n = 4 per group) was prepared by symmetrically shaving areas on both sides of the spine using an electric clipper. After 24 hours, the shaved skin was examined to ensure the absence of redness, swelling, or damage. Hydrogel samples—including PCH@EG and PCH@DMSO in various processing states (pre-annealing, post-annealing, and after 24-hour PBS immersion)—were applied to the prepared skin sites. The samples were secured using a non-irritating plastic film and medical adhesive tape for a contact duration of 4 hours (with an additional group subjected to 24-hour exposure to simulate long-term contact). As a positive control, a 10% sodium dodecyl sulfate (SDS) solution was applied to a separate skin area. Following removal of the samples, the skin was observed and photographed at 0, 1, 24, 48, and 72 hours.

1. **Immunohistochemistry (IHC)**

For IHC analysis, paraffin-embedded muscle tissue sections were processed for CD68 immunostaining. Briefly, sections were deparaffinized in xylene and rehydrated through graded ethanol series. Antigen retrieval was performed in citrate buffer (pH 6.0) at 95 °C for 20 min. Endogenous peroxidase activity was quenched with 3% H₂O₂, and the sections were then incubated overnight at 4 °C with the primary anti-CD68 antibody (GP110; dilution 1:500). Signal detection was carried out using an HRP-conjugated secondary antibody followed by visualization with 3,3'-diaminobenzidine (DAB) chromogen. Finally, the slides were counterstained with hematoxylin and imaged using a DM4000 B upright microscope (Leica Microsystems GmbH, Wetzlar, Germany).


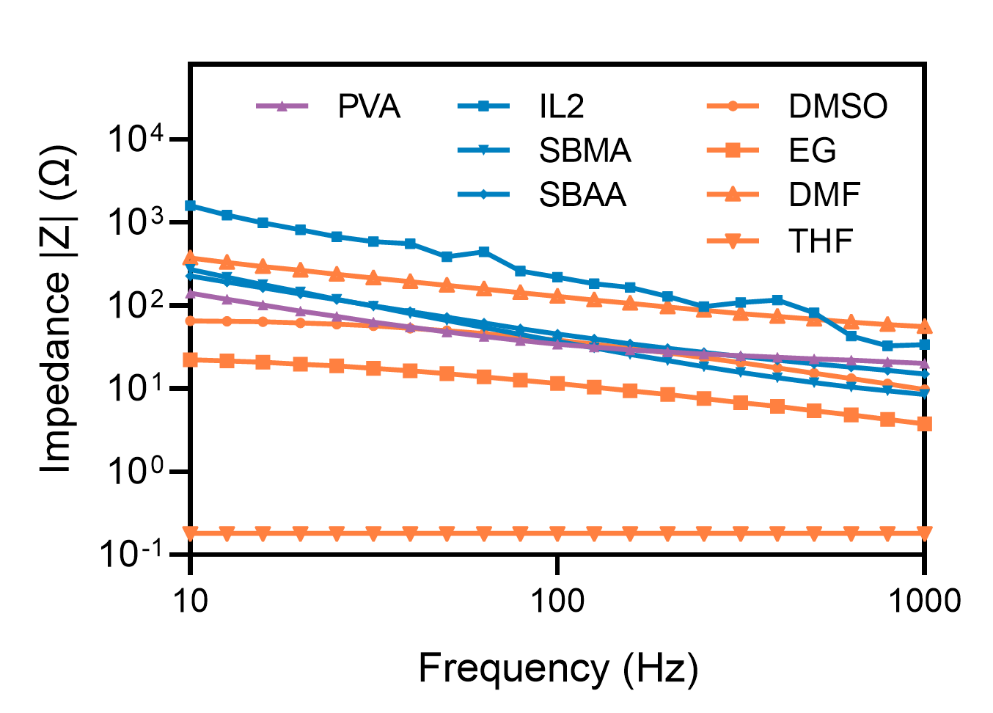


**Figure S1.** Impedance comparison and screening of IPNCH, PSCH and PCH in different formulations.


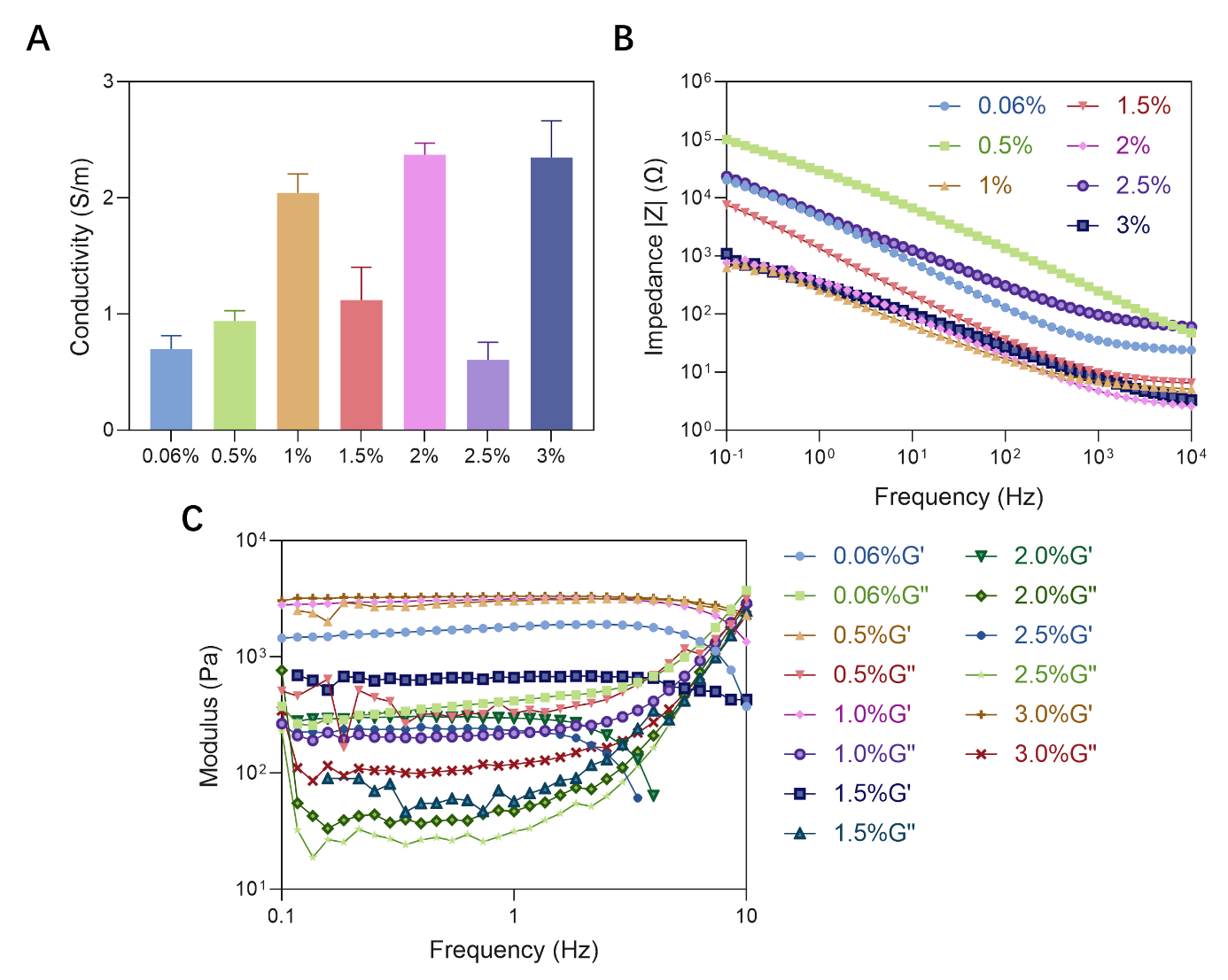


**Figure S2.** Comparison of conductivity, modulus and impedance of IPNCH@CAPAM within different PEDOT:PSS content. A. Conductivity of IPNCH@CAPAM within different PEDOT:PSS content. B. Impedance of IPNCH@CAPAM within different PEDOT:PSS content in the frequency range 0.1-10,000Hz. C. Modulus of IPNCH@CAPAM within different PEDOT:PSS content.


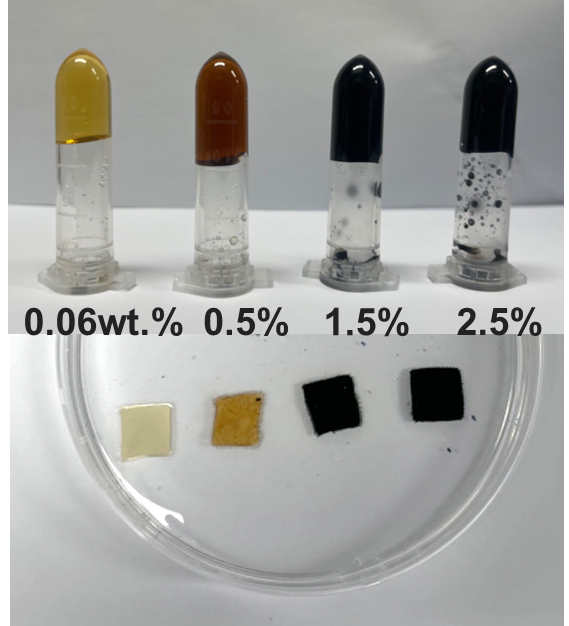


**Figure S3.** Optical images of IPNCH@CAPAM within different PEDOT:PSS content.


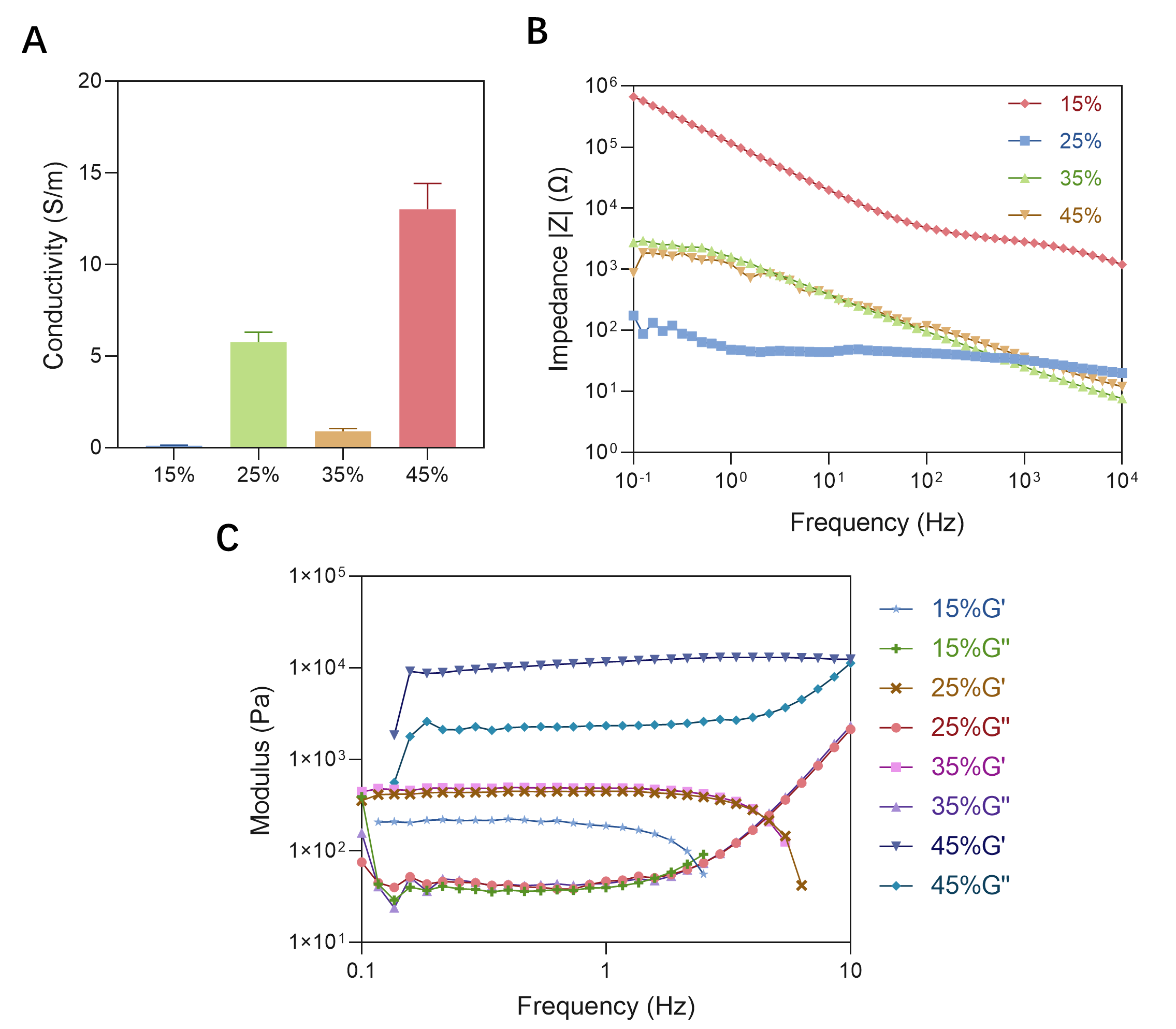


**Figure S4.** Comparison of conductivity, modulus and impedance of PSCH@SBMA within different SBMA content. A. Conductivity of PSCH@SBMA within different SBMA content. B. Impedance of PSCH@SBMA within different SBMA content in the frequency range 0.1-100,000Hz. C. Modulus of PSCH@SBMA within different SBMA content.


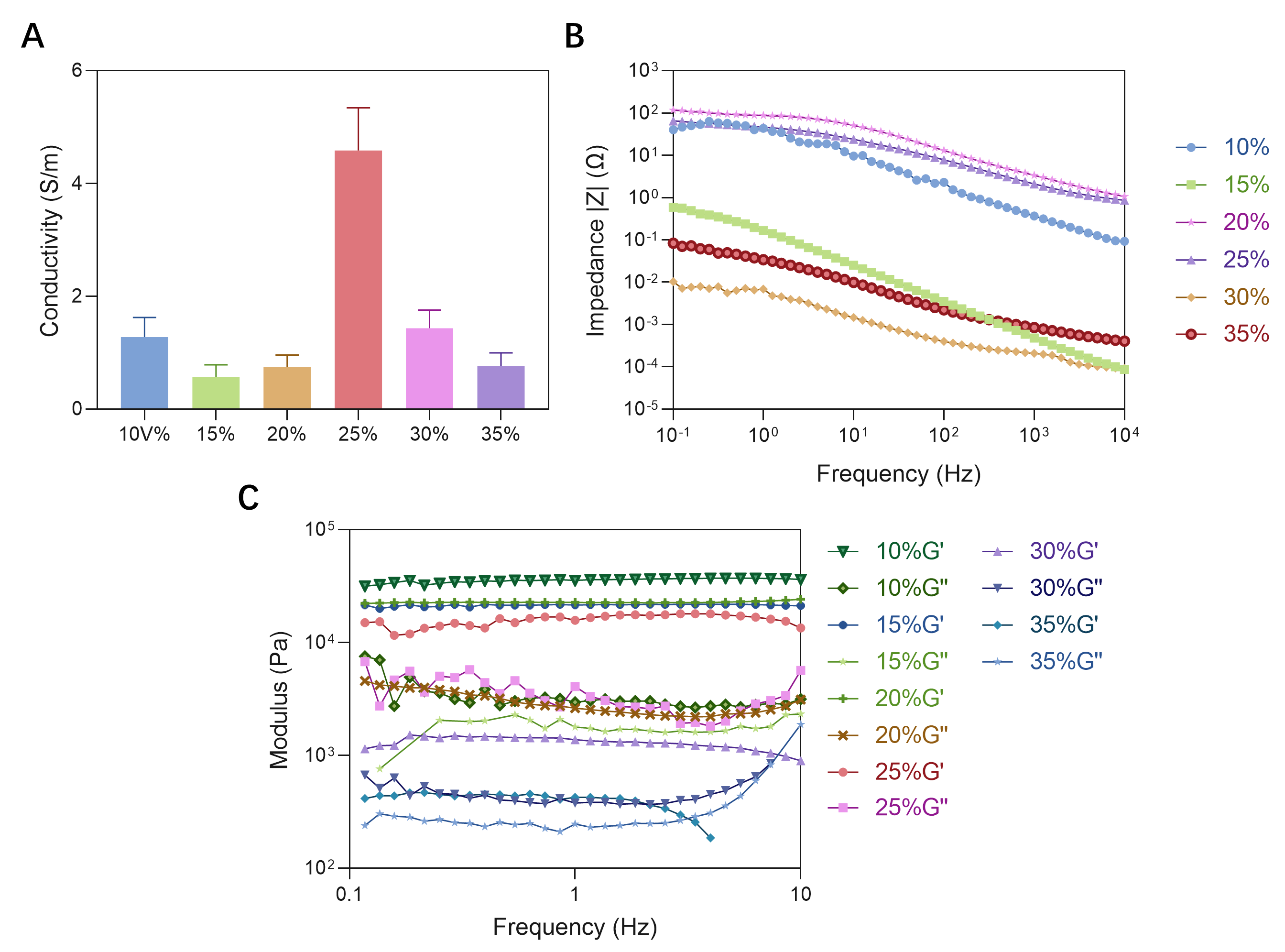


**Figure S5.** Comparison of conductivity, modulus and impedance of PCH@EG within different EG content. A. Conductivity of PCH@EG within different EG content. B. Impedance of PCH@EG within different EG content in the frequency range 0.1-10,000Hz. C. Modulus of PCH@EG within different EG content.


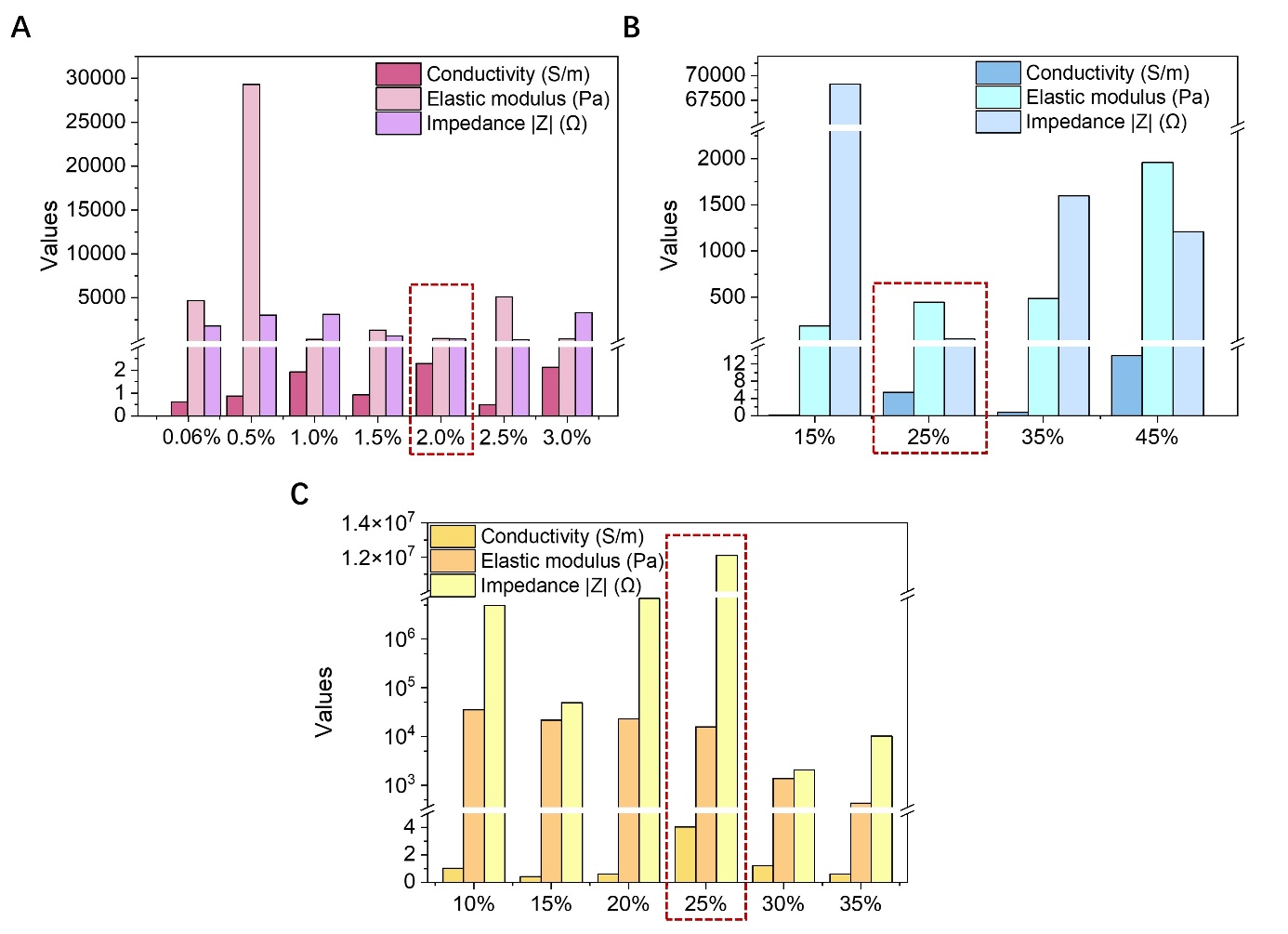


**Figure S6.** The properties of varying raw material ratios IPNCH@CAPAM, PSCH@SBMA, PCH@EG were compared and screened. The red box indicates the proportion of the formula selected by combining the characteristics of high conductivity, low impedance, and low modulus. A. Comparison of conductivity, modulus and impedance of IPNCH@CAPAM within different PEDOT:PSS content. **B.** Comparison of conductivity, modulus and impedance of PSCH@SBMA within different SBMA content. C. Comparison of conductivity, modulus and impedance of PCH@EG within different EG content.


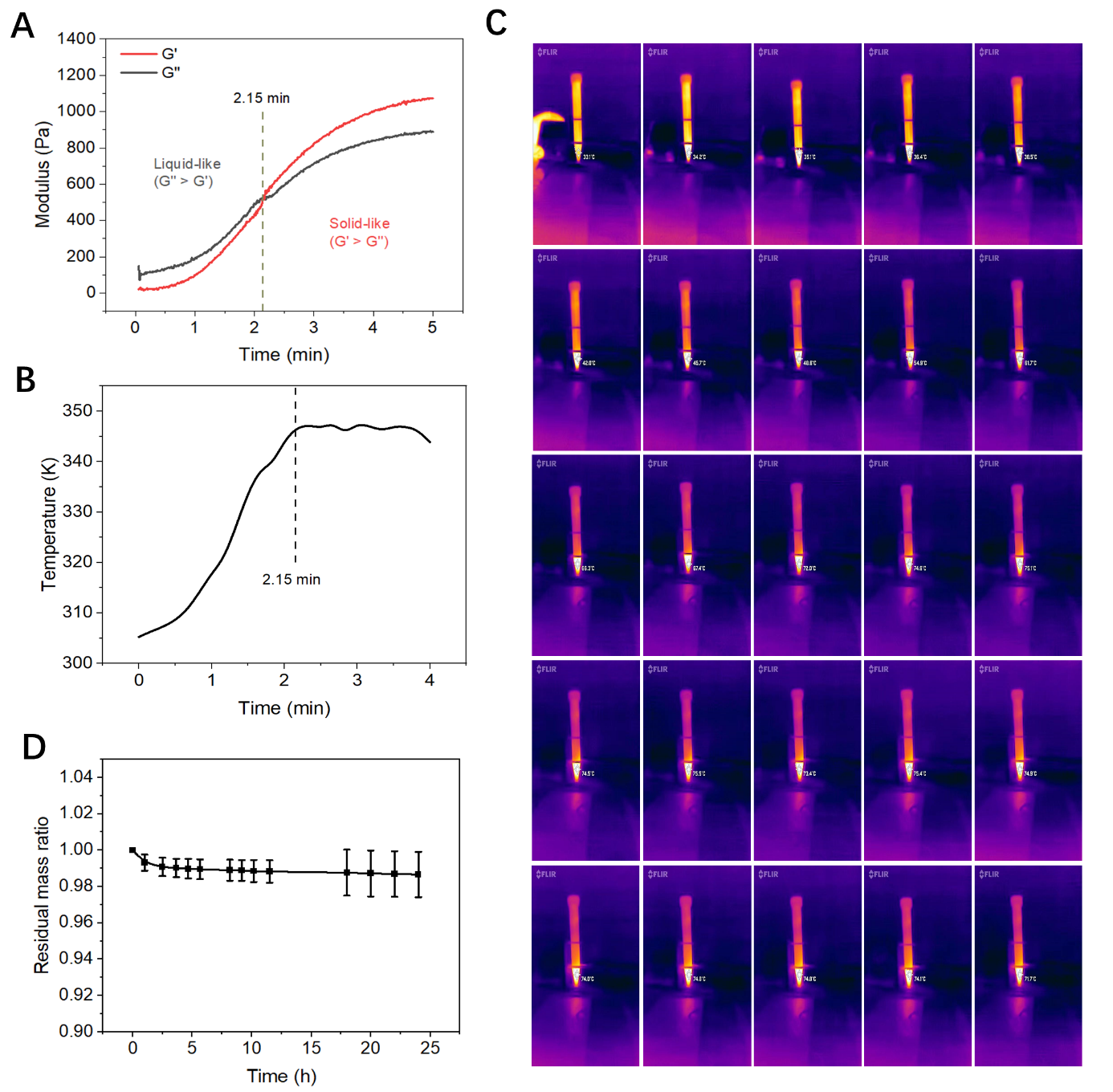


**Figure S7.** Characterization of gelling time for IPNCH@CAPAM and PCH@EG. A. Rheometer measurement of gelation time of IPNCH@CAPAM. B. Temperature change curve of the IPNCH@CAPAM system during gelling. C. Thermal imaging of IPNCH@CAPAM system during gelling. D. Residual mass ratio of PCH@EG in the gelling process as a function of time.

Notes:

For the IPNCH@CAPAM hydrogel, gelation occurs through the self-crosslinking of a PEDOT dispersion and acrylamide (AM) monomer, with BIS as the crosslinking agent and SPS as the oxidant in an alkaline dopamine reaction system. This self-crosslinking occurs at room temperature without the need for external heat or stimulation. We measured the gelling time using a rheometer, monitoring the real-time changes in the storage modulus (G') and loss modulus (G"). The point where G' surpasses G" signifies the onset of gelation, marking the formation of the crosslinked network. In this case, the gelling time was determined to be approximately 2.15 minutes (129 seconds). This result was confirmed by the tube inversion method. Additionally, thermal imaging was used to track temperature changes, and when no further increase in temperature was observed, it aligned with the gelling time, further supporting our findings.

For the PCH@EG hydrogel, the gelation process differs significantly from the other two systems, as it primarily involves solvent evaporation and multiple annealing steps rather than a conventional chemical crosslinking reaction. In this case, the mechanism of hydrogel formation is driven by physical changes — solvent removal and structural reorganization — rather than rapid chemical transitions. The procedure includes casting a solution, drying at 60°C, followed by several annealing cycles at 130°C to enhance the crystallinity and conductivity of the PEDOT.

Given the nature of this gelation process, traditional real-time measurements such as rheometry or the tube inversion method are not suitable. Instead, we monitored the sample’s weight during the drying phase, as the gelation is primarily driven by solvent evaporation. We observed that the mass of the sample stabilized after approximately 4 hours, indicating that the solvent had fully evaporated and the hydrogel structure had solidified. This mass stabilization provided an indirect measurement of the gelation time. Additionally, we visually inspected the sample throughout the process. The formation of a uniform film-like structure as the solvent evaporated further corroborated the completion of gelation.

It is essential to note that while the annealing steps improve the material’s mechanical properties and conductivity, they do not contribute directly to the gelation process. The critical phase of gelation occurs during the initial drying period at 60°C. During this phase, we regularly recorded the weight of the sample to monitor the drying process. Once the weight change became negligible, it signified that the solvent had been fully removed and the hydrogel structure had stabilized. This point marks the completion of gelation. Concurrently, we observed changes in the sample’s appearance, transitioning from a liquid (solution) state to a dry, solid (gel-like) form, which served as further confirmation of gel formation.

The experimental results demonstrate that the PCH@EG sample undergoes significant mass reduction during the drying process, particularly within the first 4 hours. Therefore, we determined the gelation time for PCH@EG to be approximately 4 hours, based on both weight stabilization and visual observations of the structural transformation.


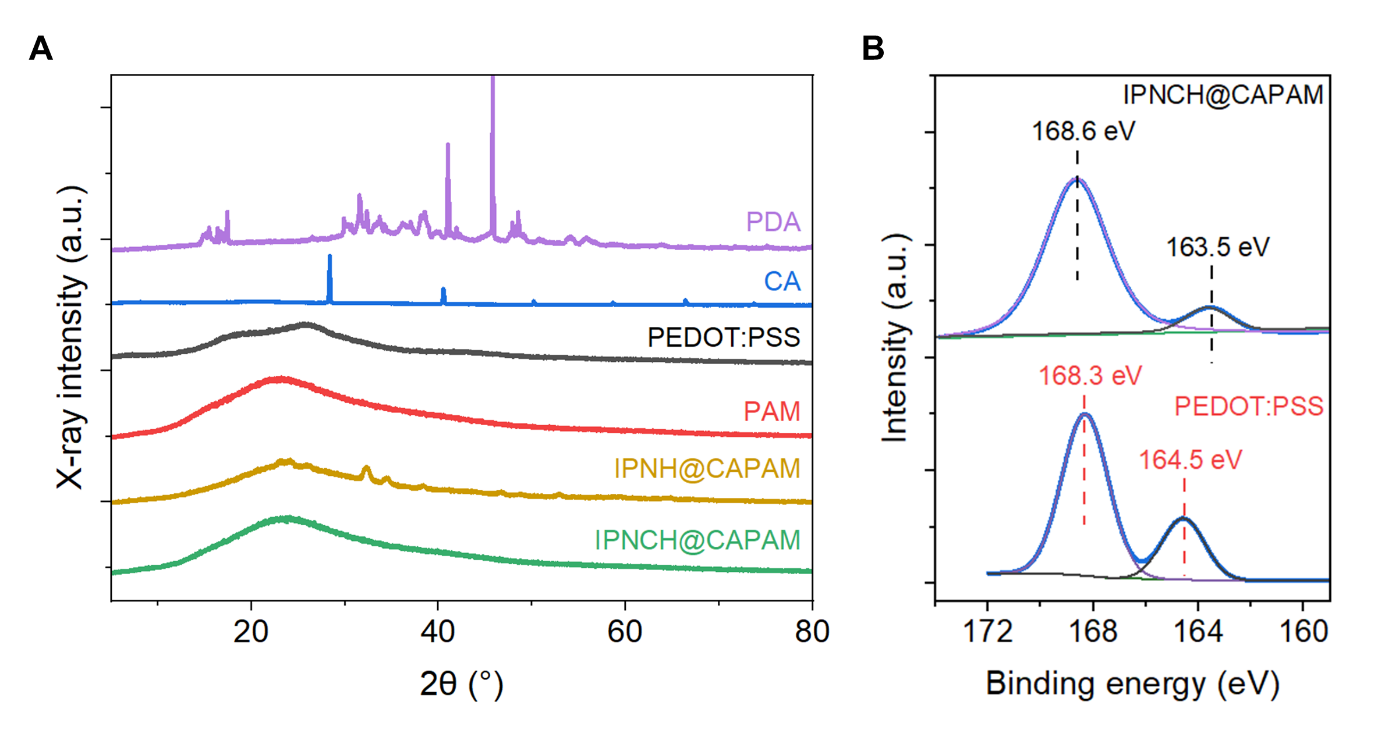


**Figure S8.** The X-Ray diffraction (XRD) pattern and the X-ray photoelectron spectroscopy (XPS) spectra of IPNCH@CAPAM. A. The XRD pattern of IPNCH@CAPAM system, where IPNH@CAPAM refers to the system without the addition of conducting polymer PEDOT:PSS. B. The peak-splitting analysis of the peaks of the S 2p spectrum in XPS spectra.

**Notes:**

The XRD patterns show that carrageenan (CA) and dopamine (PDA) exhibit sharp diffraction peaks, indicating their high crystallinity in pure form. However, in the IPNCH@CAPAM system, the crystallinity is significantly reduced due to interactions with other components. As PEDOT, a conductive polymer, typically exists in an amorphous state, this is reflected in the broad peaks observed in the XRD spectra. Additionally, PAM, being amorphous, shows wide peaks without sharp features, indicating the disordered nature of its crosslinked structure.

In the IPNCH@CAPAM system, the addition of PEDOT results in a broad envelope peak near 2θ ≈ 24°, highlighting increased interactions between PEDOT and the hydrogel matrix. This suggests that PEDOT remains amorphous in the system, forming a flexible conductive network by interacting with the hydrogel matrix. This amorphous structure enhances the system’s electrical and mechanical properties, creating a synergistic effect between these characteristics.

To further verify the distribution of PEDOT:PSS in IPNCH@CAPAM system and its cross-linking with other components, we compared the XPS test results of pure PEDOT:PSS system with those of IPNCH@CAPAM system. According to the peak-splitting analysis of the peaks of the S 2p spectrum, we observe two main binding energy regions:

To further explore the distribution of PEDOT:PSS within the IPNCH@CAPAM system and its interactions with other components, we compared the XPS spectra of pure PEDOT:PSS with those of the IPNCH@CAPAM system. In the S 2p spectrum, we identified two main binding energy regions:

168.6eV (IPNCH@CAPAM) vs. 168.3eV (PEDOT:PSS) : These peaks correspond to the sulfonic acid (-SO₃⁻) group in PSS. The retention of this peak in the IPNCH@CAPAM system indicates that PSS does not undergo significant chemical crosslinking but remains stable as a conductive component.

163.5eV (IPNCH@CAPAM) vs. 164.5eV (PEDOT:PSS) : These peaks, associated with the covalent C-S bond in PEDOT, show a slight shift in the IPNCH@CAPAM system. This shift suggests that PEDOT interacts physically with CA and PAM (e.g., through hydrogen bonding or π-π interactions), enhancing the electrical conductivity and mechanical properties of the system.


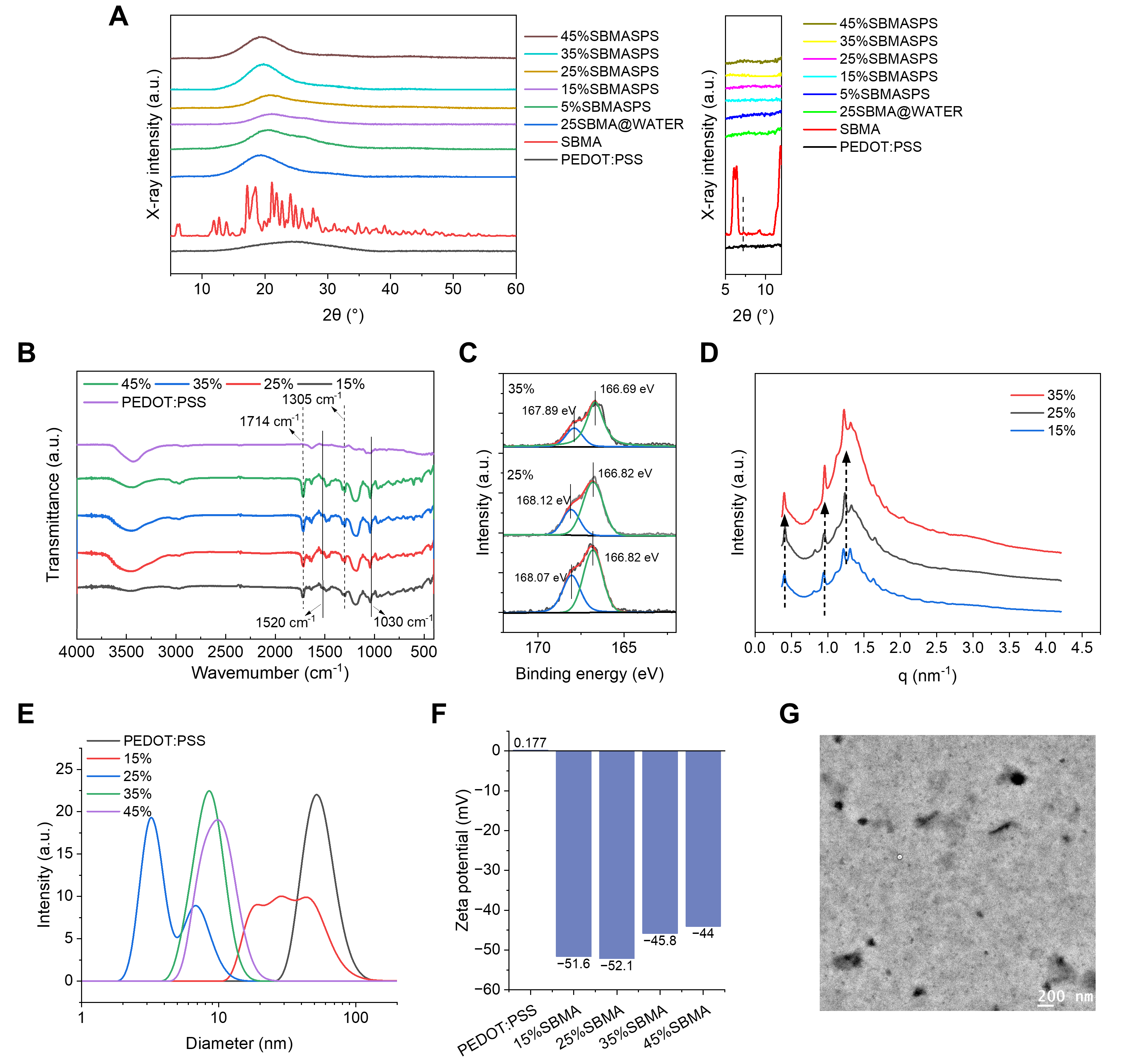


**Figure S9.** The characterization of PSCH@SBMA with different SBMA content. A. X-Ray diffraction (XRD) pattern of PSCH@SBMA. B. FT-IR spectroscopy of PSCH@SBMA. C. XPS spectroscopy in the S 2p region of PSCH@SBMA. D. WAXS profiles of PSCH@SBMA. E. Particle size distribution of PEDOT:PSS and SBMA mixed solutions with different SBMA content via dynamic light scattering (DLS) analysis. F. Zeta potential of PEDOT:PSS and SBMA mixed solutions with different SBMA content. G. TEM image of PEDOT:PSS and 25%SBMA mixed solution.

**Notes:**

The FTIR spectra reveal that the intensity of the 1714 cm⁻¹ peak—attributed to the C=O stretching vibration of the pSB network—increases with SBMA content, and subtle shifts in the PEDOT C=C (~1520 cm⁻¹) and PSS S=O (~1030 cm⁻¹) bands are observed (Fig. S9B). These spectral changes indicate that the electrostatic shielding effect of the pSB network progressively weakens the strong electrostatic interactions between PEDOT and PSS, thus promoting the rearrangement of these domains.

Notably, XPS data showed that at 25 wt% SBMA, the binding energy difference between the S 2p peaks of PEDOT (stable at 166.82 eV) and PSS/pSB (increasing slightly from 168.07 eV at 15% to 168.12 eV at 25%) reached a slightly larger ΔE of 1.30 eV (Fig. S9C). This suggests that at 25% SBMA, the electrostatic shielding provided by pSB is most effective. The nearly identical PEDOT peak at 166.82 eV for both 15% and 25% indicates that the chemical state of PEDOT is stable. However, the slight increase in the S⁶⁺ binding energy (from 168.07 to 168.12 eV) and the corresponding increase in ΔE at 25% SBMA indicate a more pronounced differentiation between the PEDOT and PSS/pSB domains. This implies that the phase separation is optimized at 25%, leading to a more extended, conductive PEDOT network embedded in a pSB-rich matrix. In contrast, at 35% SBMA, both peaks shift downward and ΔE decreases to 1.20 eV, suggesting that excessive SBMA alters the chemical environment—likely due to overabundance of sulfonate groups—which may diminish the shielding effect and hinder the ideal domain rearrangement.

Furthermore, the WAXS data provided clear evidence of changes in the crystalline structure. In the WAXS spectra (Fig. S9D), the diffraction peaks at q values corresponding to approximately 0.4, 0.9, and 1.2 nm⁻¹ become notably stronger and sharper when the SBMA content increases. This enhancement indicates that the PEDOT-rich domains undergo significant recrystallization and chain rearrangement, forming a well-ordered and interconnected conductive network. DLS data demonstrated a significant reduction in the hydrodynamic size at 25% SBMA, suggesting that the large aggregates typically observed in pure PEDOT:PSS were disrupted by the electrostatic effects of the pSB network (Fig. S9E). This is supported by Zeta potential measurements, which showed an increased absolute value, reflecting improved dispersion stability and reduced aggregation (Fig. S9F). TEM imaging further confirmed the open, interconnected network in the 25% SBMA sample (Fig. S9G).


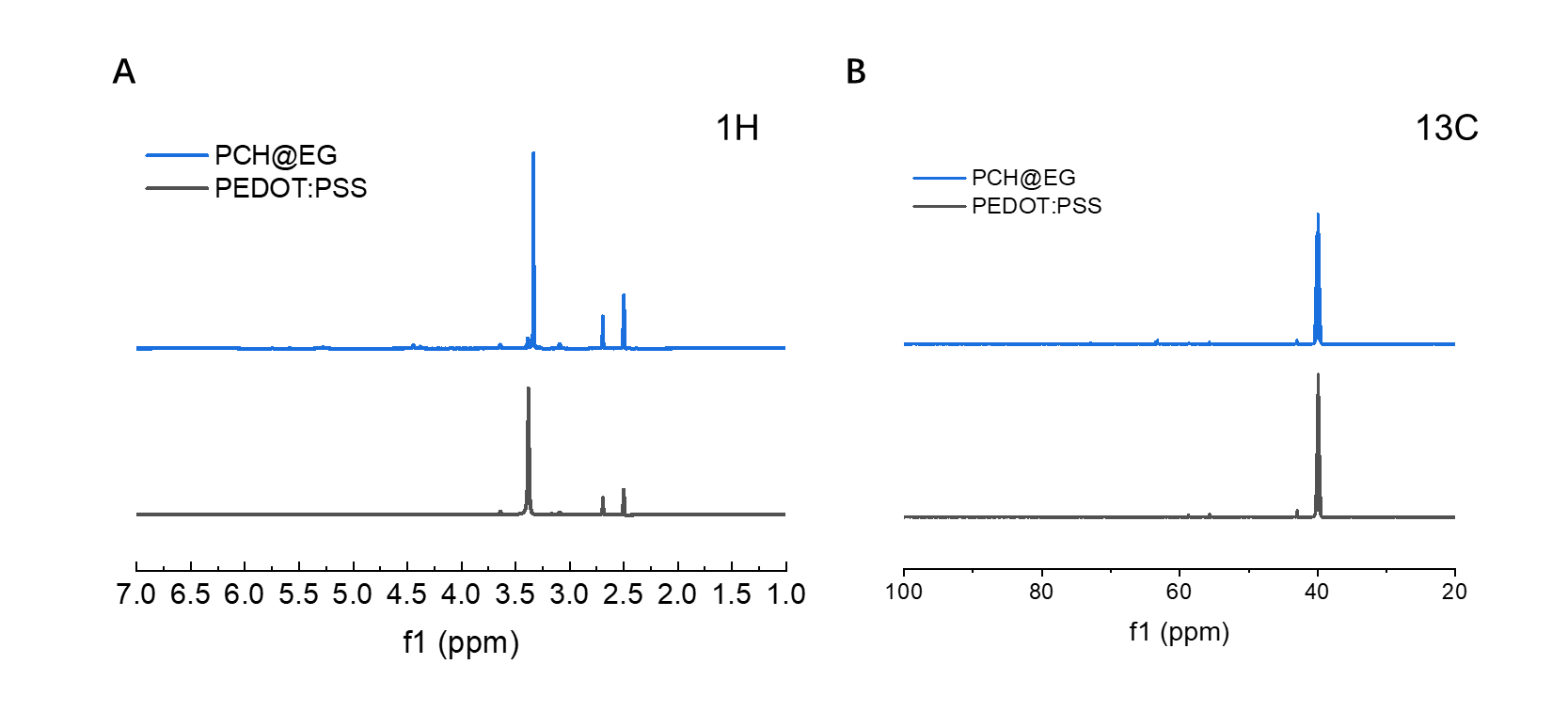


**Figure S10.** Nuclear magnetic resonance (NMR) spectroscopy of PCH@EG. A. ^1^H NMR of PCH@EG. B. ^13^C NMR of PCH@EG.


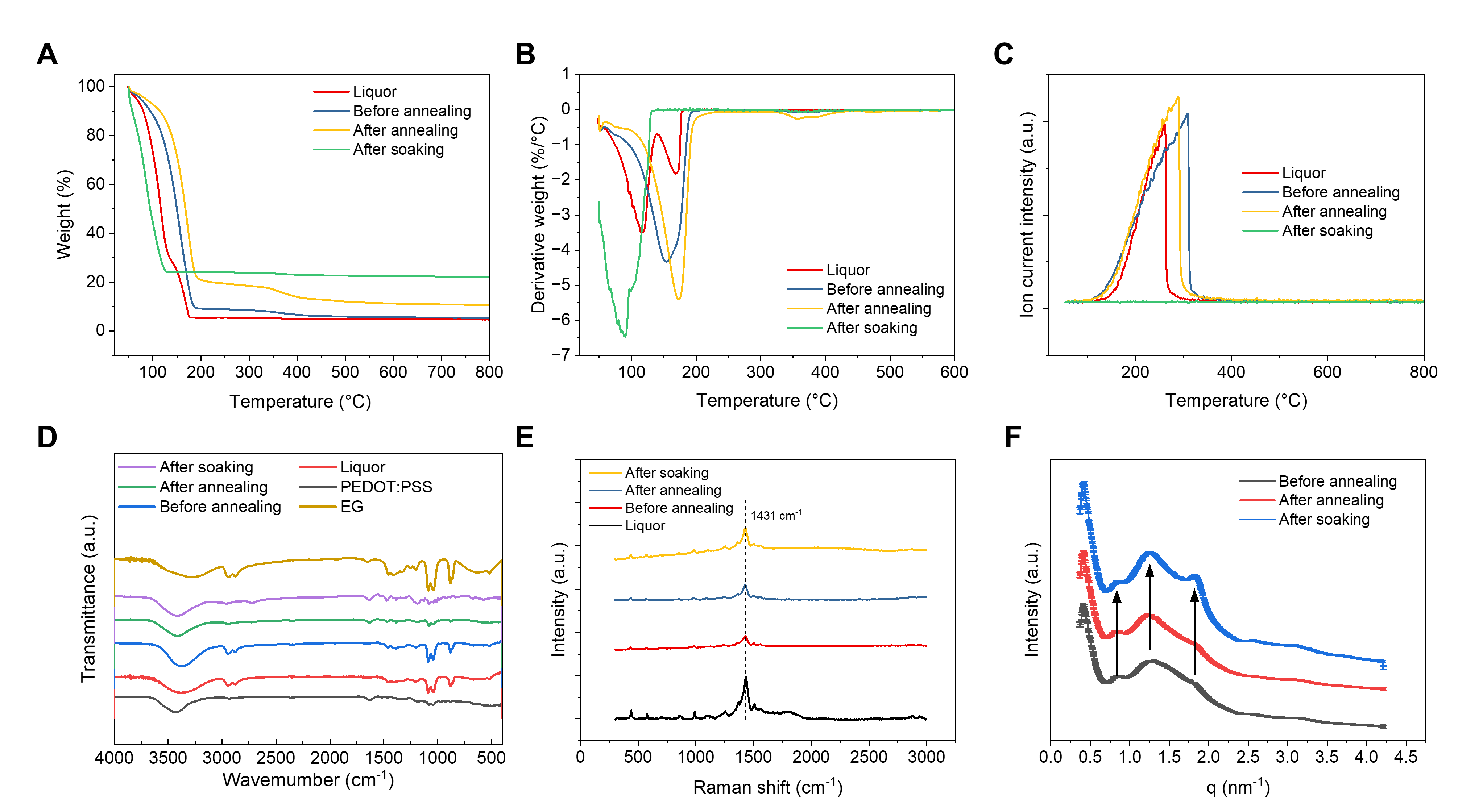


**Figure S11.** Characterization of PCH@EG as mixed solution group, before annealing, after annealing, and after soaking. A. TGA curves ranging from 50 to 800°C. B. DTA curves ranging from 50 to 800°C. C. The signal at m/z = 31 in TG-MS analysis. D. FT-IR spectroscopy of PCH@EG. E. RAMAN spectroscopy of PCH@EG. F. WAXS profiles of PCH@EG.


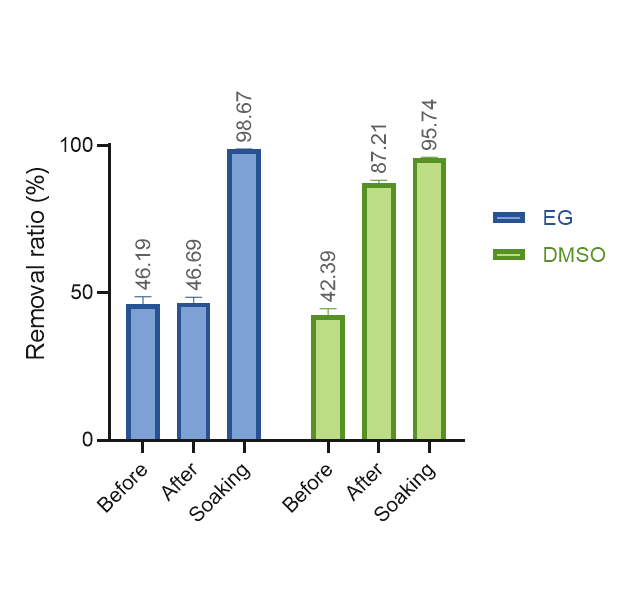


**Figure S12.** The removal ratios of polar solvents in PCH after annealing and soaking processes. The residual solvents in the extraction solution were quantitatively determined using liquid chromatography (for EG) and gas chromatography (for DMSO).


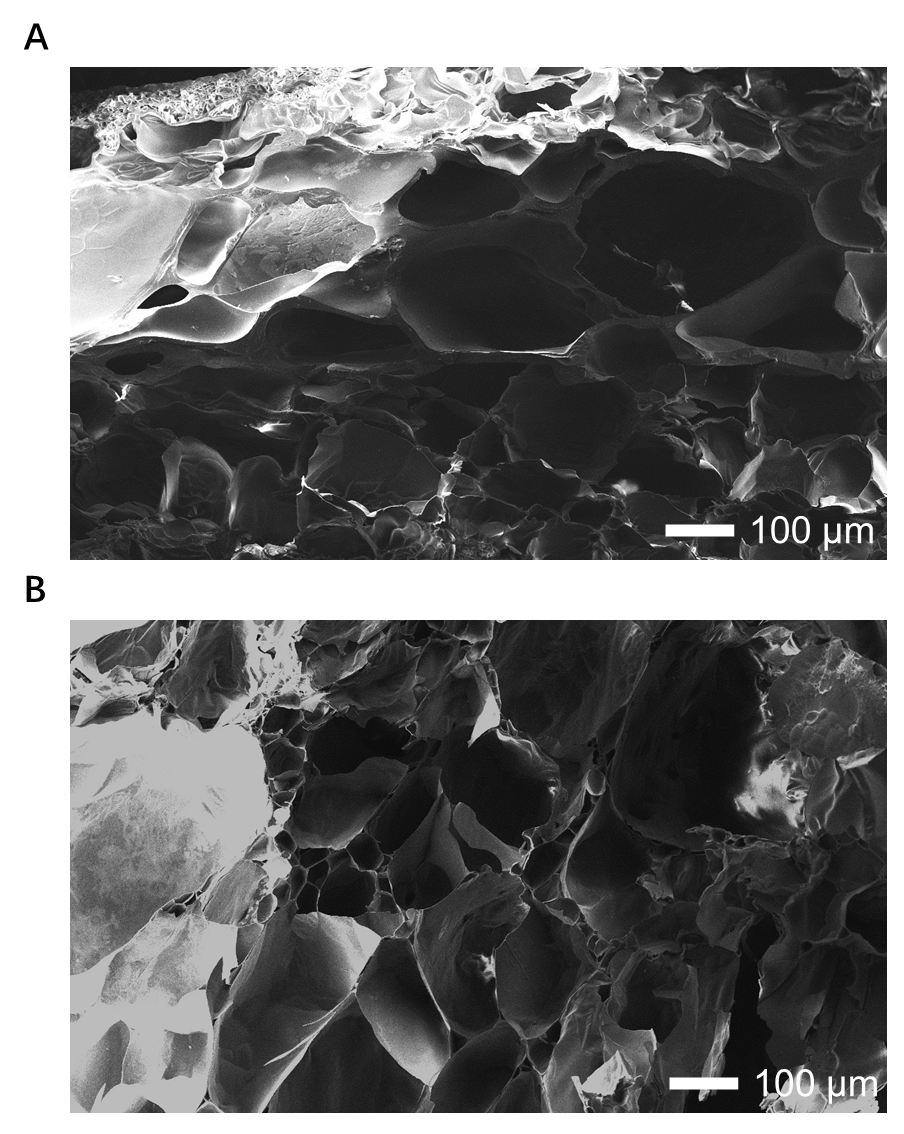


**Figure S13.** SEM images of IPNCH@CAPAM. A. SEM images of IPNCH@CAPAM. B. SEM images of IPNCH@CAPAM after soaking water for 50 days in room temperature.


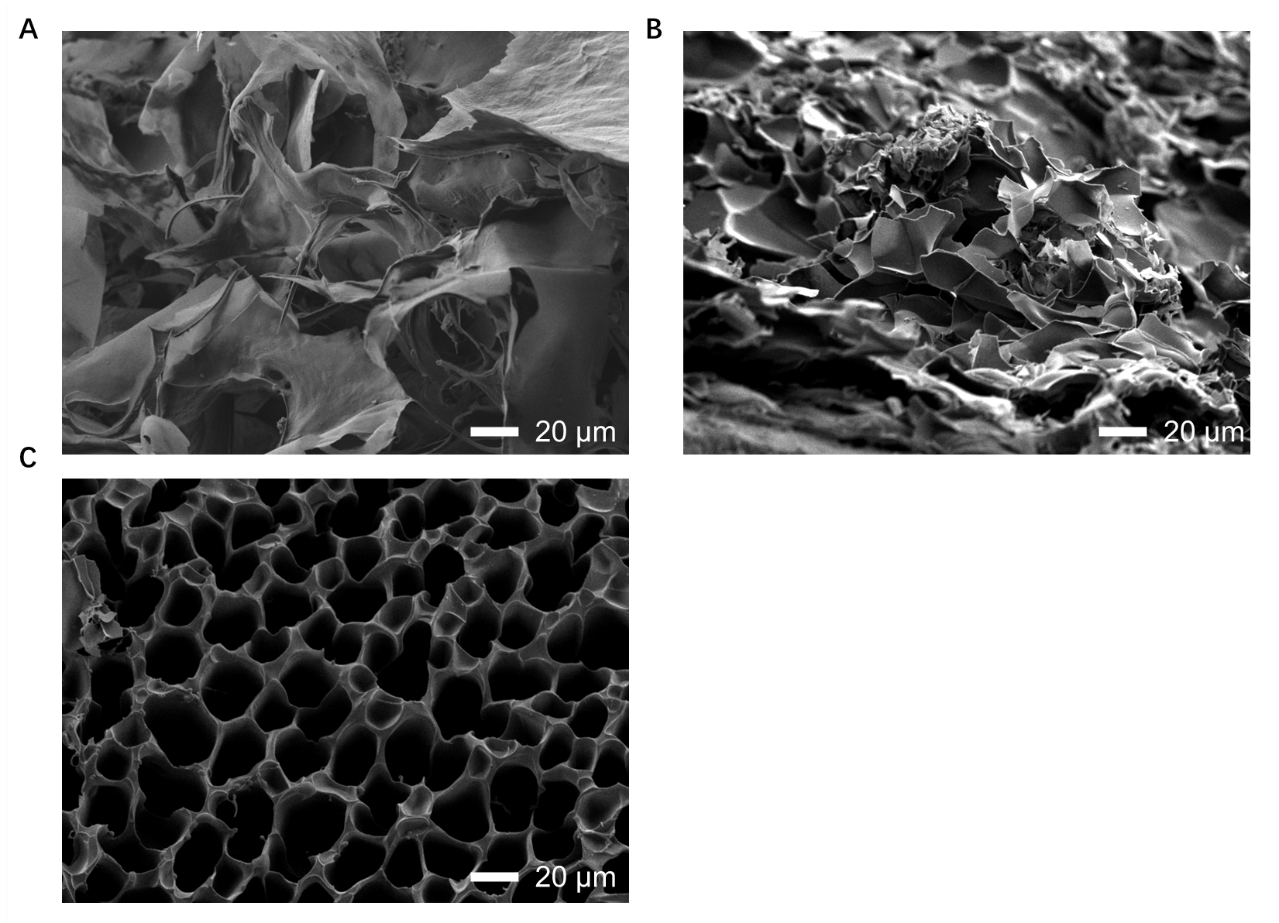


**Figure S14.** SEM images of PSCH@SBMA within different SBMA content. A. SEM image of PSCH@SBMA hydrogel of 0 wt.% SBMA. B. SEM image of PSCH@SBMA hydrogel of 25 wt.% SBMA. C. SEM image of PSCH@SBMA hydrogel of 35 wt.% SBMA.


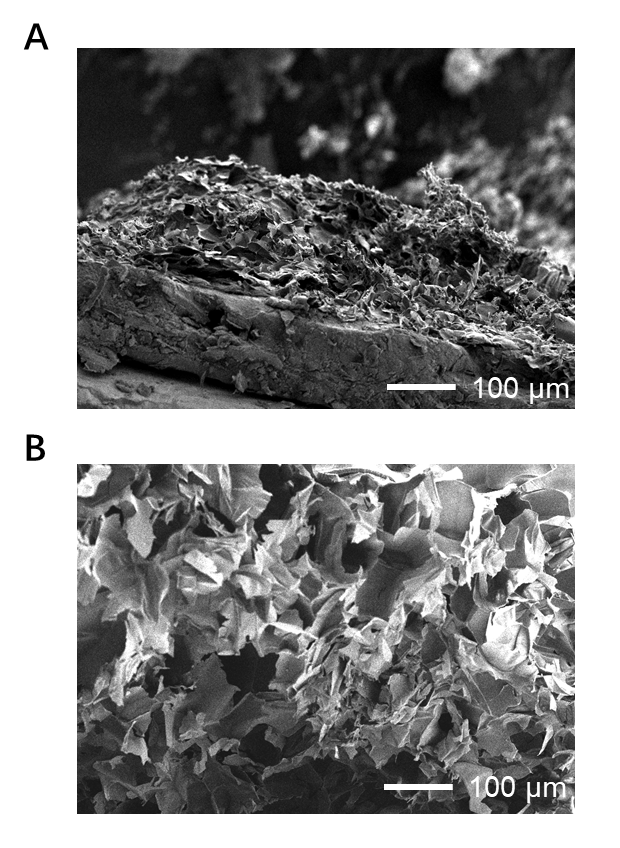


**Figure S15.** SEM images of PSCH@SBMA. A. SEM images of PSCH@SBMA. B. SEM images of PSCH@SBMA after soaking water for 50 days in room temperature.


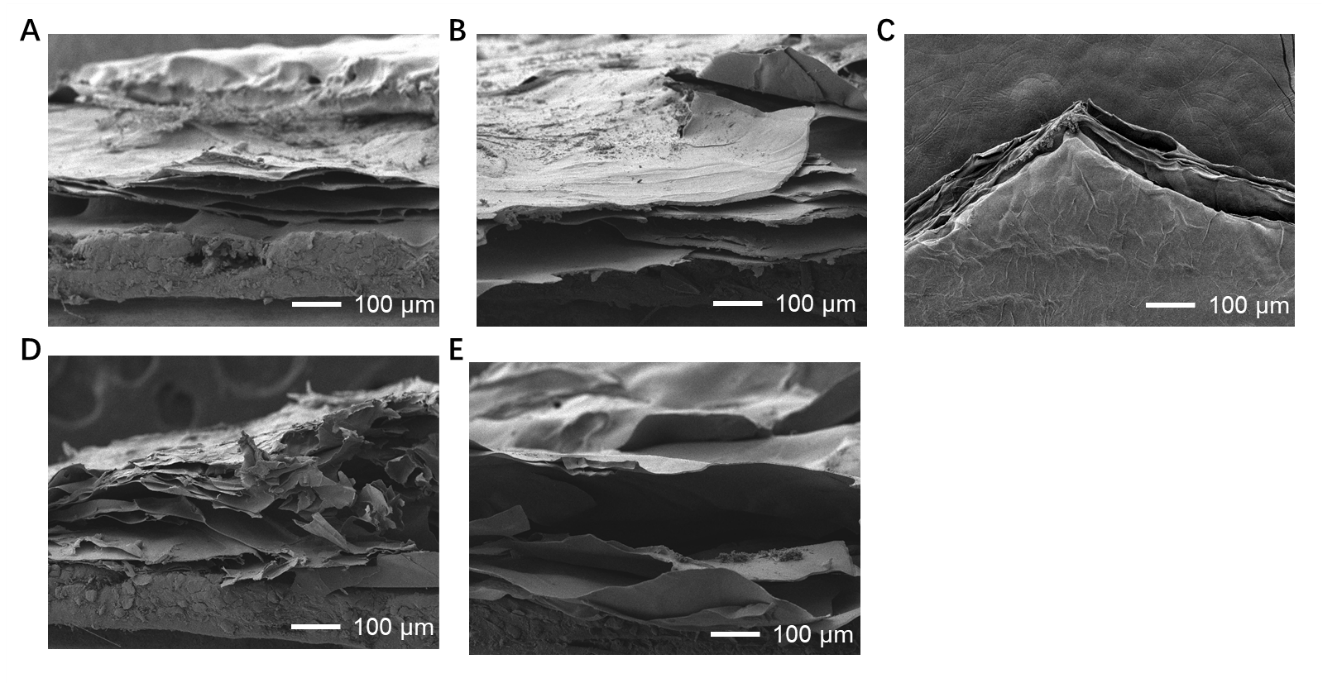


**Figure S16.** SEM images of PCH@EG within different EG content. A. SEM image of PCH@EG hydrogel of 10 vol.% EG. B. SEM image of PCH@EG hydrogel of 15 vol.% EG. C. SEM image of PCH@EG hydrogel of 25 vol.% EG. D. SEM image of PCH@EG hydrogel of 30 vol.% EG. E. C. SEM image of PCH@EG hydrogel of 35 vol.% EG.


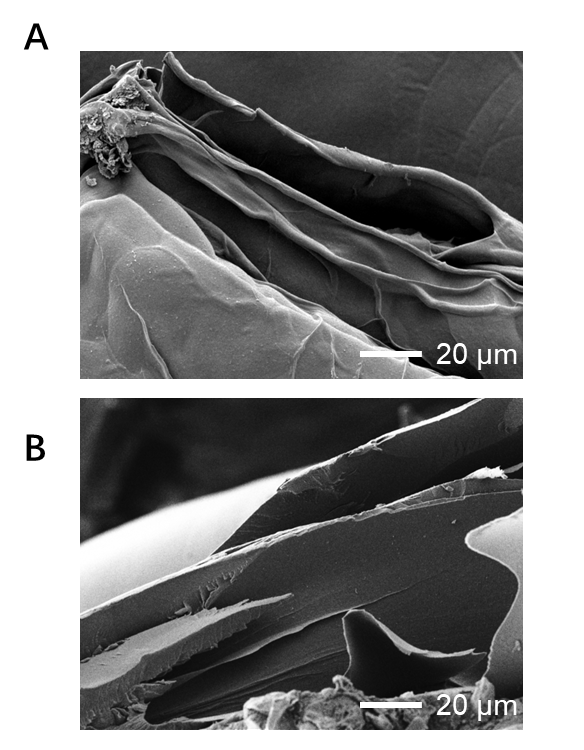


**Figure S17.** SEM images of PCH@EG. A. SEM images of PCH@EG. B. SEM images of PCH@EG after soaking water for 50 days in room temperature.


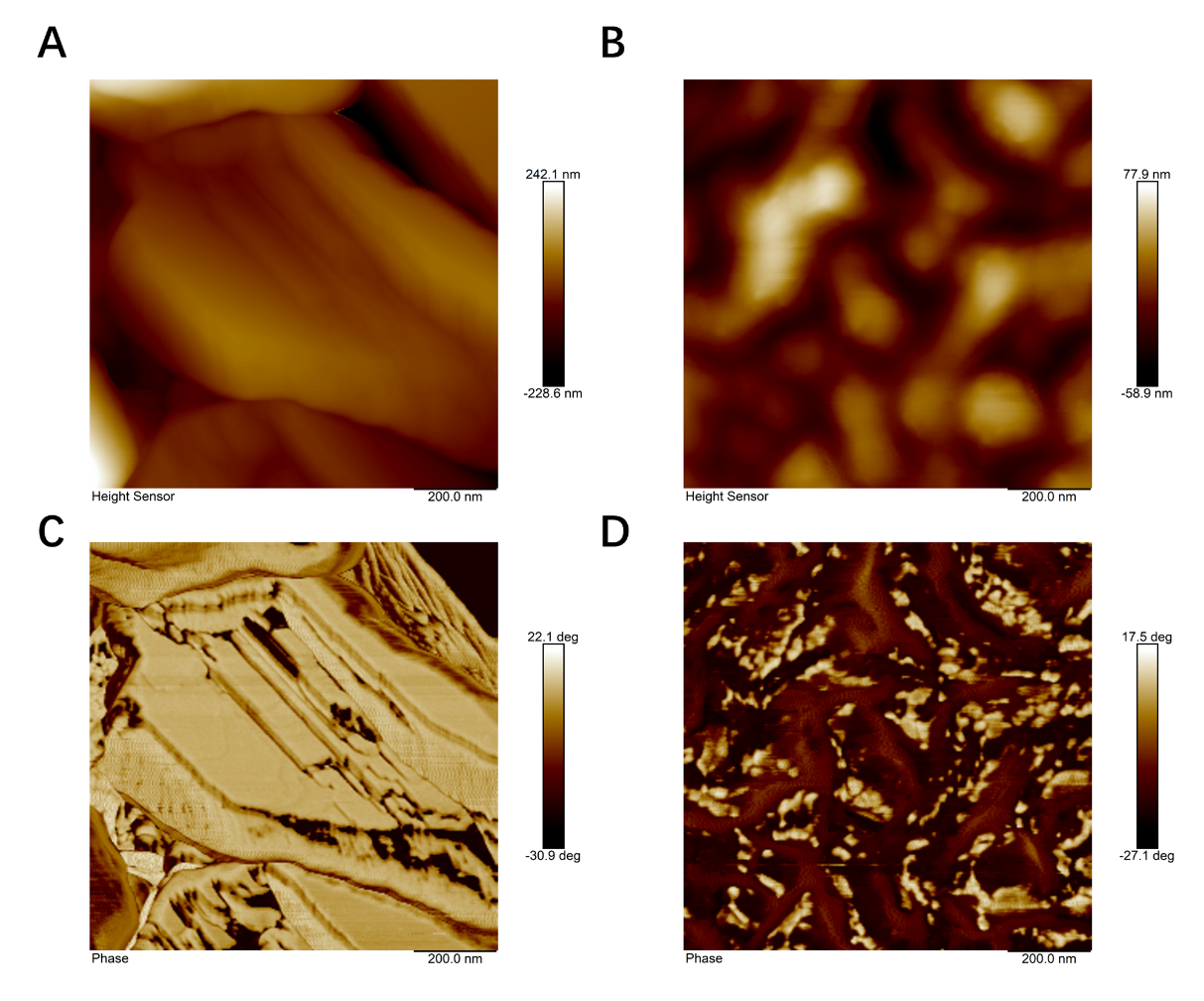


**Figure S18.** Morphology and phase diagrams of PSCH@SBMA within different SBMA content. A. AFM morphology diagram of PSCH@SBMA of 25 wt.% SBMA, and the average surface roughness Ra=31.7 nm. B. AFM morphology diagram of PSCH@SBMA of 35 wt.% SBMA, and the average surface roughness Ra=14.2 nm. C. AFM phase diagram of PSCH@SBMA of 25 wt.% SBMA. D. AFM phase diagram of PSCH@SBMA of 35 wt.% SBMA.


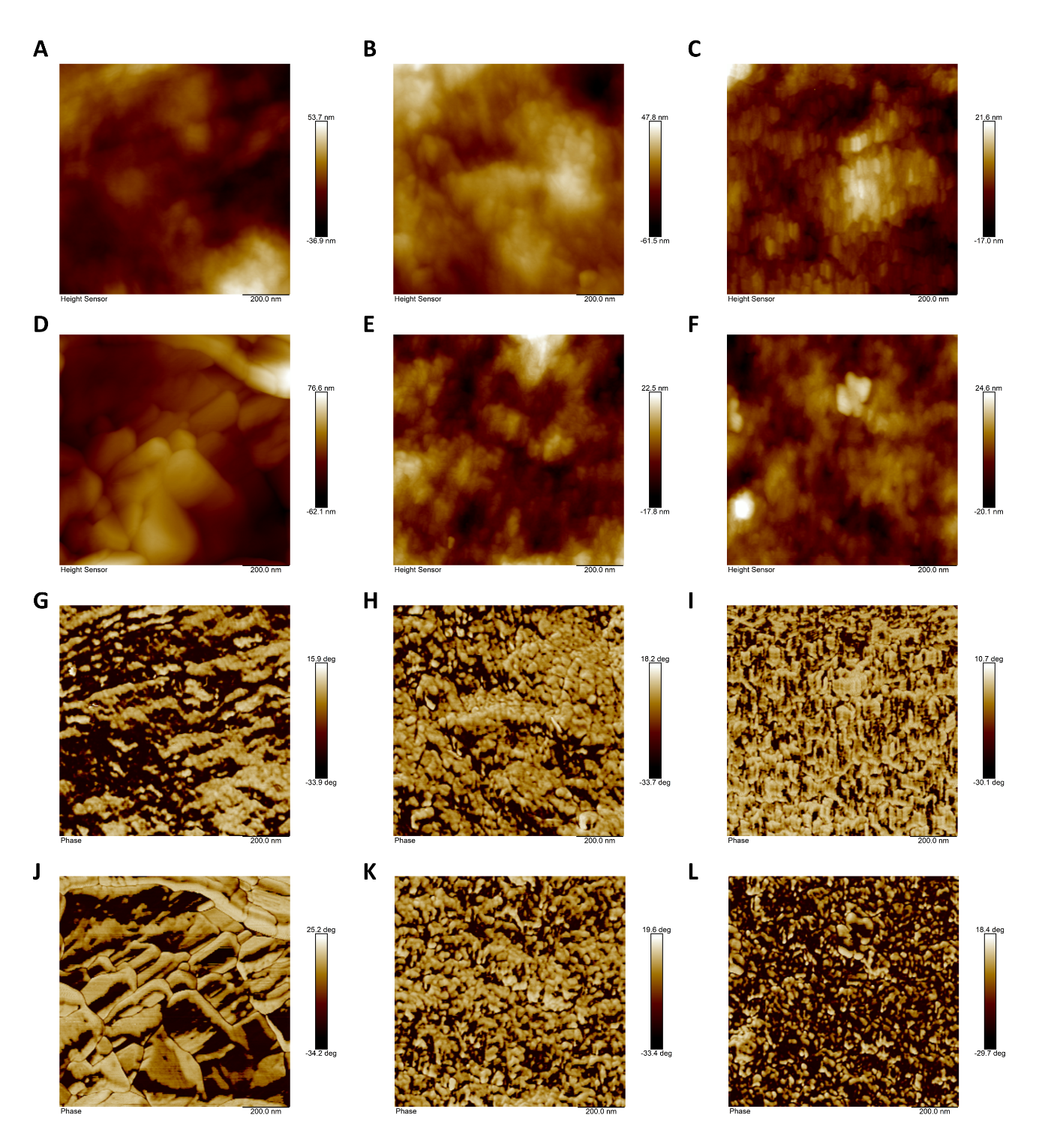


**Figure S19.** Morphology and phase diagrams of PCH@EG within different EG content. A. AFM morphology diagram of PCH@EG of 10 vol.% EG, and the average surface roughness Ra=5.12 nm. B. AFM morphology diagram of PCH@EG of 15 vol.% EG, and the average surface roughness Ra=7.14 nm. C AFM morphology diagram of PCH@EG of 20 vol.% EG, and the average surface roughness Ra=3.45 nm. D. AFM morphology diagram of PCH@EG of 25 vol.% EG, and the average surface roughness Ra=12.0 nm. E. AFM morphology diagram of PCH@EG of 30 vol.% EG, and the average surface roughness Ra=3.62 nm. F. AFM morphology diagram of PCH@EG of 35 vol.% EG, and the average surface roughness Ra=4.00 nm. G. AFM phase diagram of PCH@EG of 10 vol.% EG. H. AFM phase diagram of PCH@EG of 15 vol.% EG. I. AFM phase diagram of PCH@EG of 20 vol.% EG. J. AFM phase diagram of PCH@EG of 25 vol.% EG. K. AFM phase diagram of PCH@EG of 30 vol.% EG. L. AFM phase diagram of PCH@EG of 35 vol.% EG.


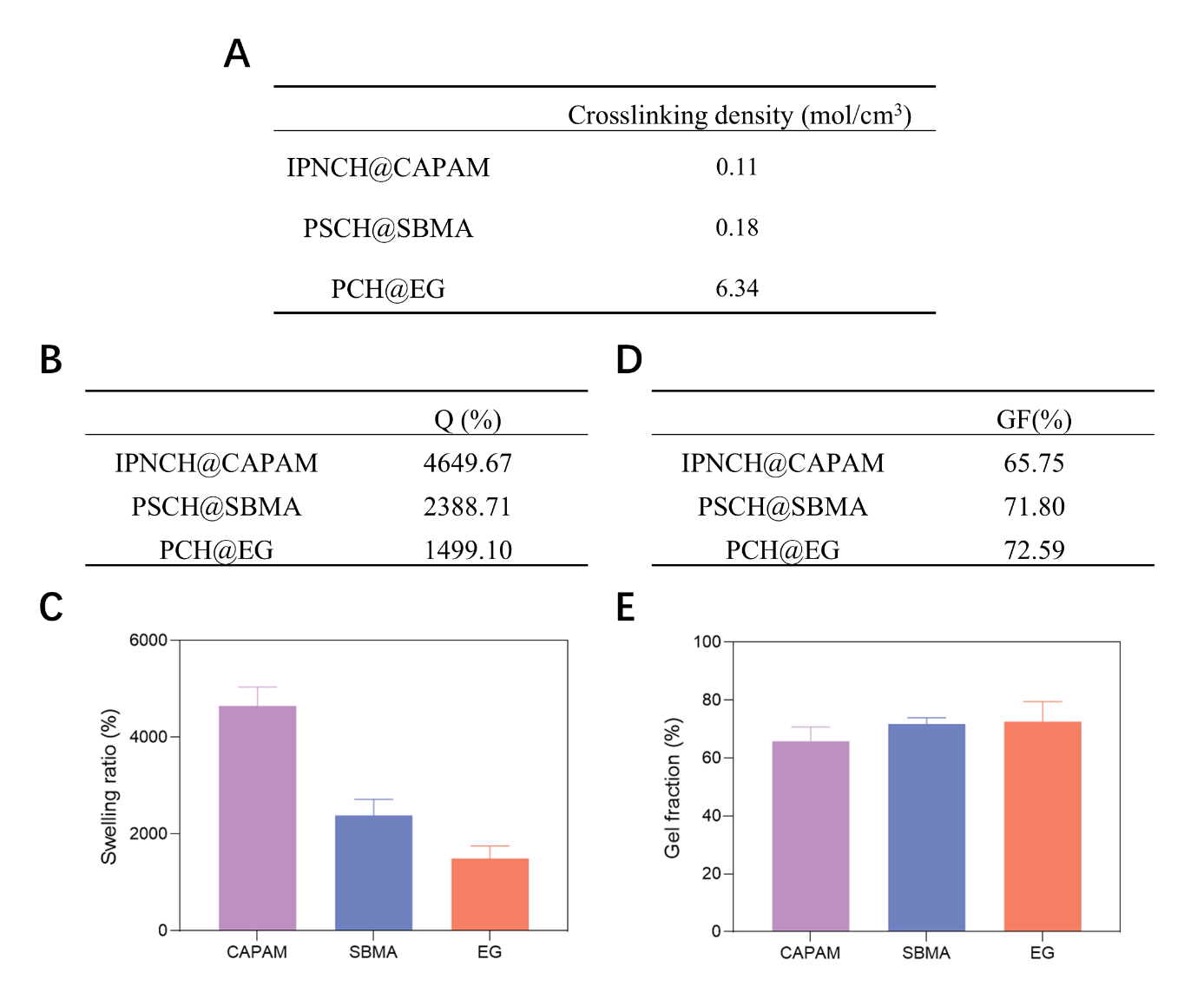


**Figure S20.** Cross-linking efficiency of IPNCH@CAPAM, PSCH@SBMA, and PCH@EG. A. Cross-linking density quantized by shear modulus. B-C. Swelling ratio of the three hydrogels. D-E. Gel fraction of the three hydrogels.


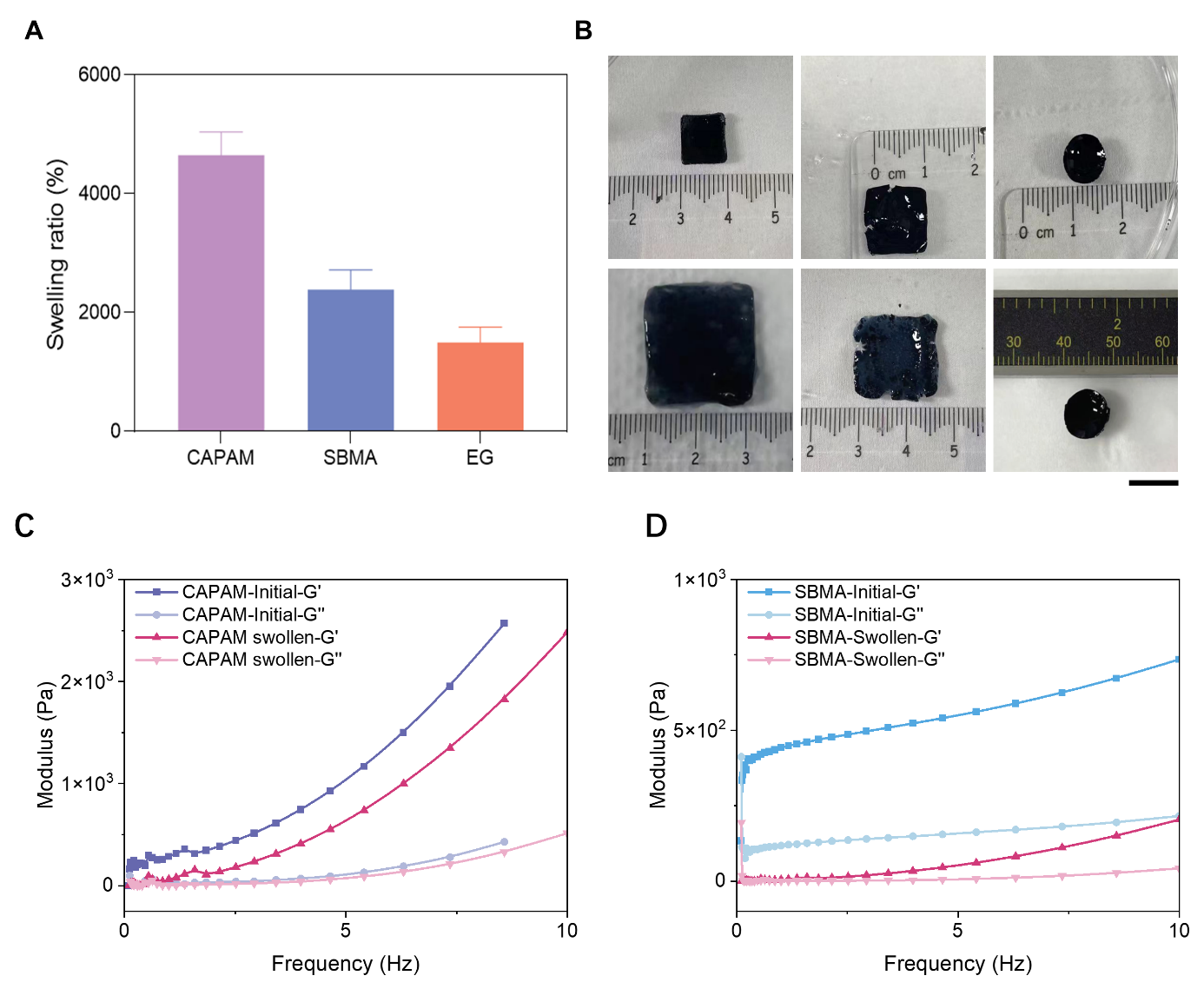


**Figure S21.** Swelling properties of IPNCH@CAPAM, PSCH@SBMA, and PCH@EG. A. Swelling ratio of the three hydrogels. B. Optical photos of the three hydrogels before and after swelling. Scale bar: 1 cm. C-D. Effect of swelling on modulus of IPNCH@CAPAM and PSCH@SBMA.

**Notes:**

The variation in swelling ratios significantly impacts the mechanical properties and adhesion of these hydrogels. Generally, a higher swelling ratio indicates a greater capacity for water absorption, which can compromise mechanical strength and adhesion due to the reliance of the network structure on water.

To investigate how swelling affects the mechanical properties of the hydrogels, we fully swelled the samples in normal saline and assessed changes in the modulus before and after swelling. The results demonstrate a significant reduction in the shear modulus of the hydrogel following swelling, indicating a decrease of several orders of magnitude. This decrease is primarily attributed to the softening of the hydrogel's network structure. Upon complete swelling, the hydrogel absorbs a substantial amount of water, causing its network to expand and form a looser three-dimensional structure. In this state, the hydrogel exhibits softer, more fluid properties, leading to diminished mechanical strength and tensile capabilities. Consequently, the hydrogel struggles to maintain the same levels of stress and strain as observed in its unswollen state.

In addition to altering mechanical properties, water content also influences adhesion. After full swelling, the hydrogel's surface becomes saturated with water, creating a sliding layer that reduces direct contact with tissues or other substrate materials. This results in a decrease in adhesion, further impacting the hydrogel's overall performance in practical applications.


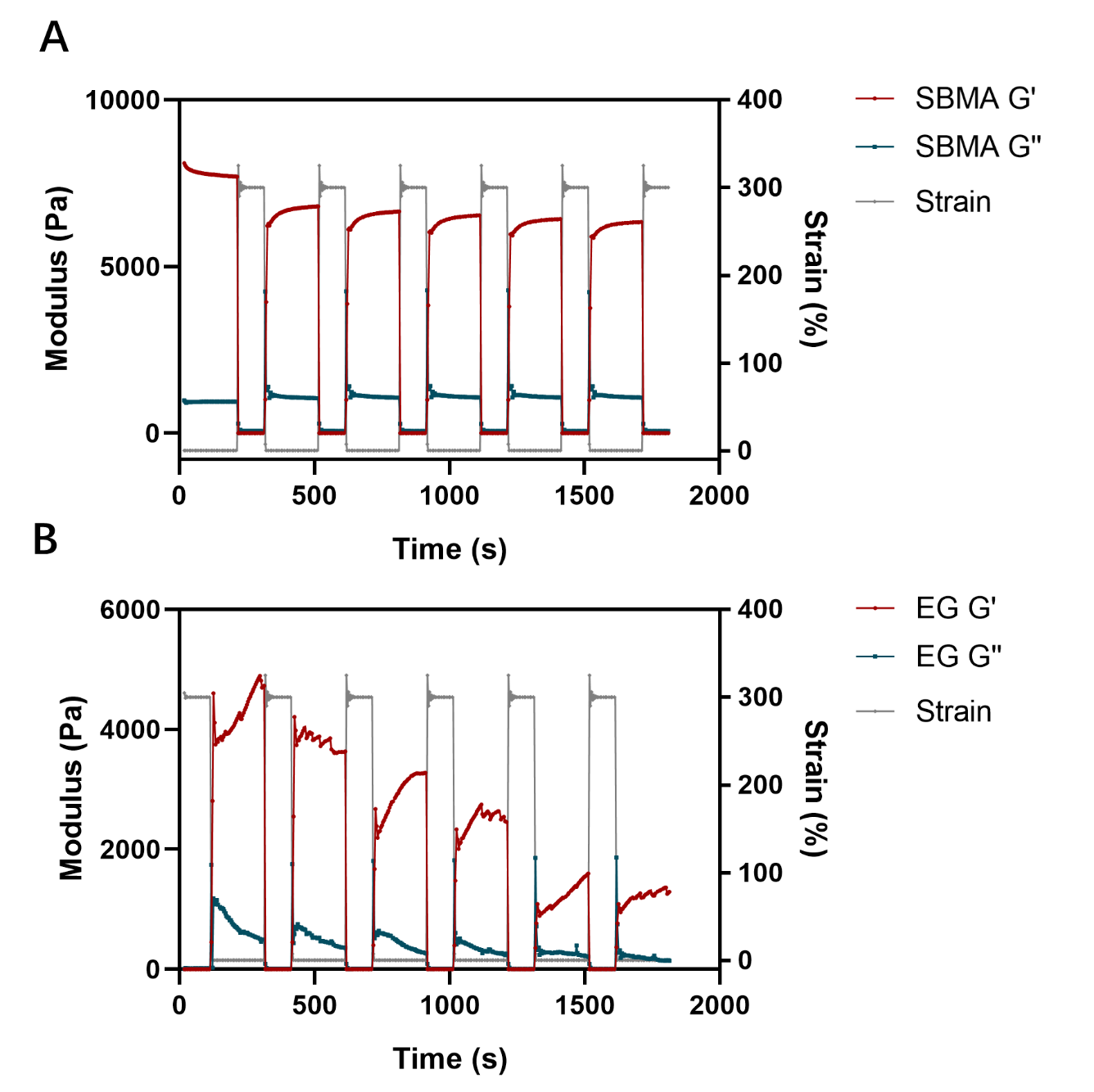


**Figure S22.** Step strain scanning of conducting polymer hydrogels. A. Step strain scanning of PSCH@SBMA. B. Step strain scanning of PCH@EG.


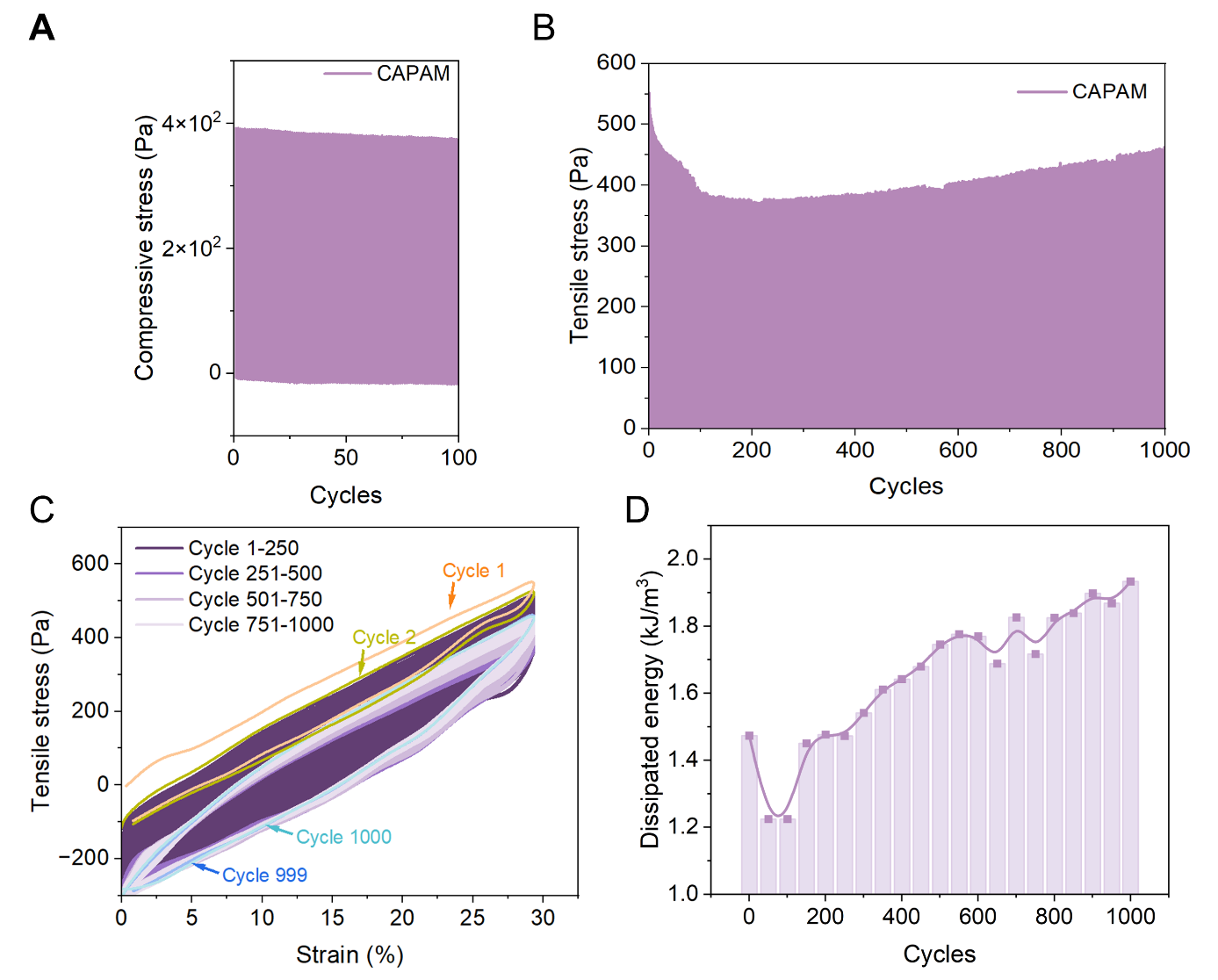


**Figure S23.** Repeated tensile/compression test for fatigue resistance of IPNCH@CAPAM. A. Stress curves for 100 successive compression load-unload cycles at 20% strain for IPNCH@CAPAM. B. Stress curves for 1000 successive tension load-unload cycles at 30% strain for IPNCH@CAPAM. C. Stress-strain curves for 1000 successive tension load-unload cycles at 30% strain for IPNCH@CAPAM. D. Change of dissipated energy during the 1000 successive tension load-unload cycles.

Notes:

For the 1000-cycle tensile test on the IPNCH@CAPAM system, Figure S20B shows that the stress remained relatively stable throughout the cycles. The initial maximum stress was 572.8 Pa, which decreased to 478.0 Pa after 1000 cycles, reflecting a 16.6% decrease. This indicates some stress attenuation but overall good mechanical stability. The stress-strain curve (Figure S23C) reveals a rapid decrease in stress during the initial cycles, stabilizing after about 150 cycles. This early decline is likely due to the breaking of weaker physical or chemical crosslinks in the hydrogel’s molecular network under initial strain. The combination of the rigid PEDOT structure and the soft PAM network contributes to this initial stress drop. Furthermore, the partial dehydration of carrageenan (CA) during the early cycles exacerbates the stress reduction. However, as the system reaches a stable state after initial molecular rearrangement, the stress stabilizes. The slight increase in stress after 150 cycles may be due to water evaporation, which increases the molecular chain density and enhances tensile resistance. The energy dissipation curve (Figure S23D) also shows an initial decrease in energy dissipation, followed by a gradual rise, reflecting the molecular chain’s adaptation to the stress over time.


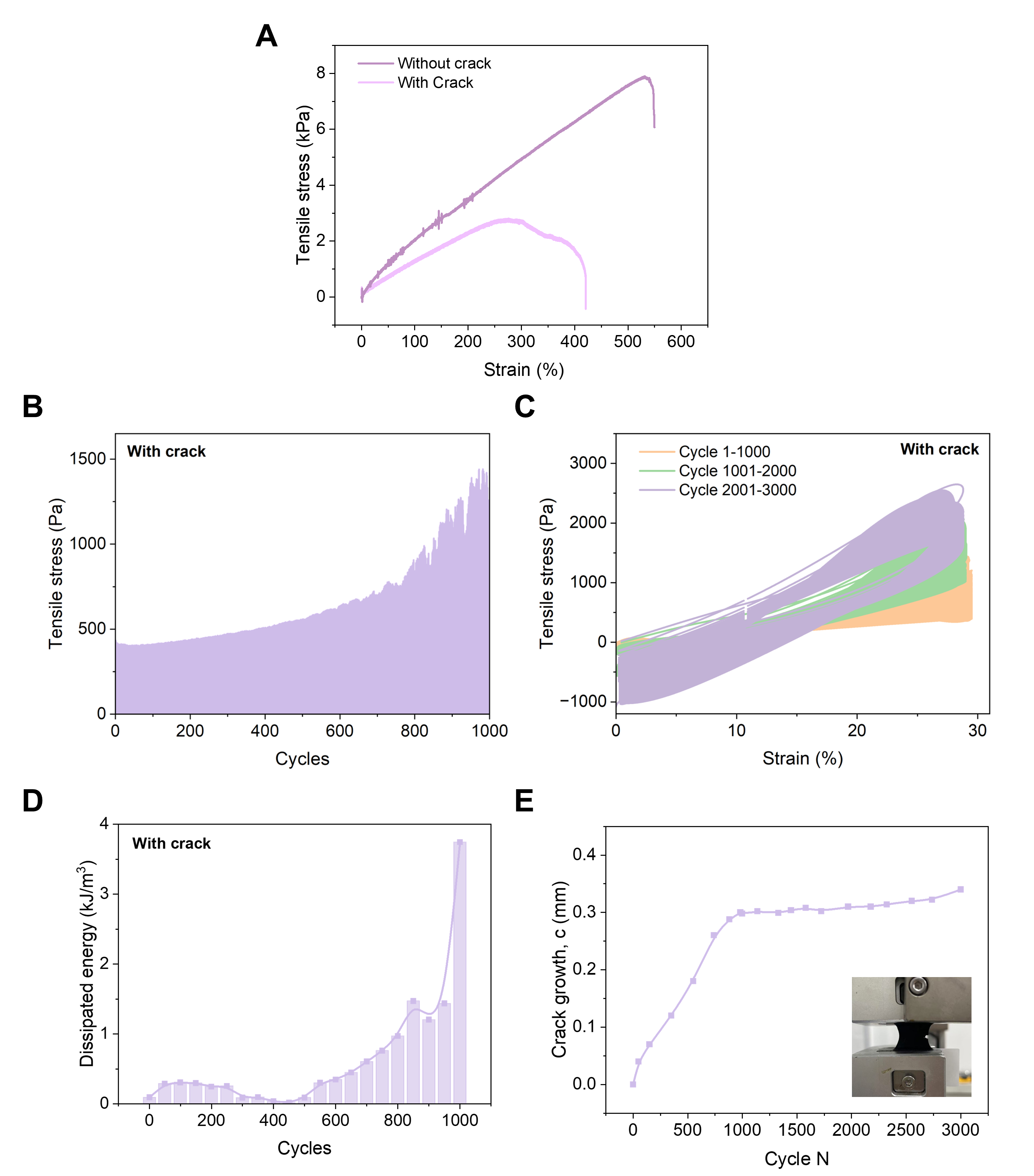


**Figure S24.** Fatigue crack test of IPNCH@CAPAM. A. Stress-strain curves of single tensile test with and without cracks. B. Variation of stress with the number of cycles during 30% strain cyclic tensile test with cracks. C. Stress-strain curves of 3,000 cycles with cracks. D. Variation of dissipated energy with cycles during 30% strain cyclic tensile test with cracks. E. Variation of strain growth with the number of cycles during 30% strain cyclic tensile test with cracks.

**Notes:**

Following the method of Rivlin and Thomas, rectangular specimens with a length-to-width ratio of L:3/5L were prepared, and a pre-existing notch of 1/5L was introduced at the mid-plane. The gauge length was set at 1/5L. First, uniaxial tensile tests were conducted to determine the fracture strain (Fig. S24A). Subsequently, using a WDW-3020 universal testing machine (Changchun Kexin Test Instrument Co., Ltd., Changchun, China), specimens were subjected to 3000 cycles of 30% tensile strain at a speed of 50 mm/min, consistent with the conditions used in Fig. 4F. Crack propagation was continuously monitored in real-time using a camera (Fig. S24B–E).

As shown in Fig. S24A, the stress-strain curves of samples with and without pre-existing cracks revealed that the presence of a crack reduced both the fracture strain and energy absorption capacity. Nonetheless, the fracture strain of the cracked samples still exceeded 270%, far surpassing the typical strain demands of biological tissues (e.g., skin, which rarely experiences strains above 30%). Based on the stress–strain curves, the strain energy per unit area before fracture, W(λ), was calculated as 4354.05 J/m³, and the critical energy release rate Gc was determined as 59.87 J/m² using the relation:

Gc = W(λ) × H

where H is the gauge length.

Fig. S24B illustrates the stress evolution over the first 1000 cycles at 30% strain, showing a gradual increase in stress with the number of cycles, particularly after 500 cycles — likely due to water loss from the hydrogel. A similar trend was observed in the stress–strain curves for 3000 cycles (Fig.S24 C). The energy dissipation during the first 1000 cycles was quantified (Fig. S24D) based on the area enclosed by the stress–strain curves. Dissipated energy decreased initially and then gradually increased, reflecting the evolving adaptation of polymer chains under cyclic stress. Fig. S24E presents the cumulative crack growth data, which increased progressively over 3000 cycles, with the initial crack length extending by 71%. This indicates a controlled yet continuous plastic deformation process in the material.

Comparing the first 1,000 cycles of specimens with and without pre-existing cracks under identical 30% strain revealed that the presence of a crack initially reduced dissipated energy due to decreased effective stiffness and more efficient load transfer (Fig. S23D). However, as fatigue cycles progressed, crack growth and local structural damage intensified, resulting in a marked increase in energy dissipation. For example, while uncracked specimens maintained relatively stable energy dissipation values around 1.5–1.9 J/m³, cracked specimens exhibited an increase to 3.74 J/m³ by the 1,000th cycle. This divergence underscores the accelerating deterioration induced by crack propagation.


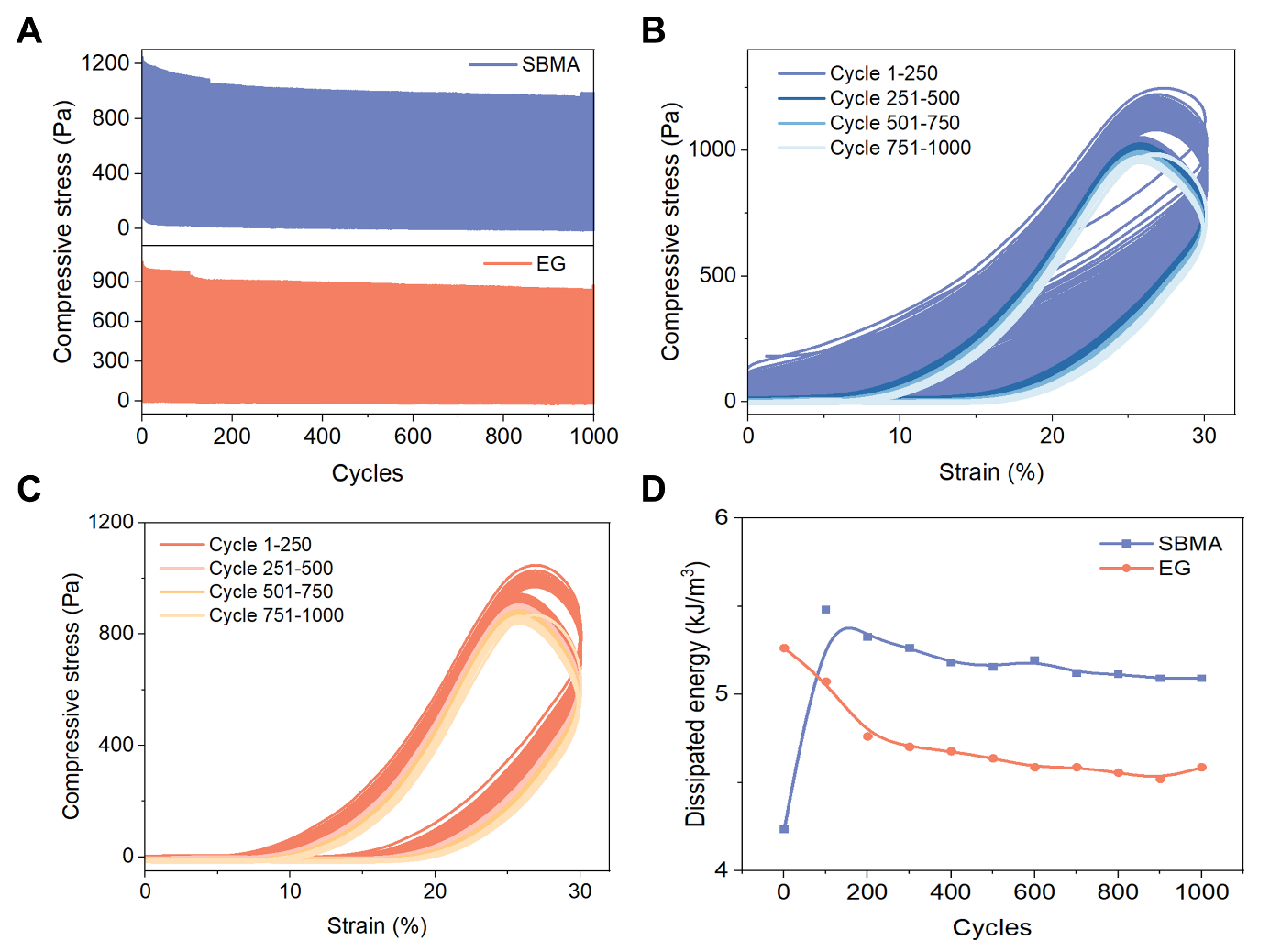


**Figure S25.** Repeated compression test for fatigue resistance of PSCH@SBMA and PCH@EG. A. Stress curves for 1,000 successive compression load-unload cycles at 30% strain for. B. Stress-strain curves for 1,000 successive compression load-unload cycles at 30% strain for PSCH@SBMA. C. Stress-strain curves for 1,000 successive compression load-unload cycles at 30% strain for PCH@EG. D. Change of dissipated energy during the 1000 successive compression load-unload cycles.

Notes:

For the PSCH@SBMA hydrogel, the stress reduction after 1000 compression cycles was 20.8%, slightly higher than that of the IPNCH@CAPAM system. The PSCH@SBMA system, combining PEDOT:PSS with ionic SBMA, demonstrates good ionic conductivity and mechanical stability. However, the weaker interactions between SBMA and PEDOT lead to faster structural relaxation and initial stress reduction. Over time, the flexibility of PSS contributes to a more stable stress response in later cycles. The energy dissipation curve also shows a decline with increasing cycles, with a 7.1% reduction by the end. The low initial energy dissipation may be due to weak physical interactions between PEDOT and SBMA, which stabilize as the internal structure rearranges during the cycles.

The PCH@EG hydrogel showed a stress reduction of 16.9% after 1000 compression cycles, demonstrating stability comparable to IPNCH@CAPAM. In this system, ethylene glycol (EG) enhances the flexibility and conductivity of PEDOT. The early stress drop may be attributed to weak initial interactions between PEDOT and EG, leading to molecular rearrangement. However, as EG improves flexibility, the system demonstrates better stability in later cycles. Energy dissipation in the PCH@EG system also gradually decreases, with a 12.7% reduction, eventually stabilizing. This suggests that the internal molecular chains reach equilibrium after initial rearrangement, reducing energy dissipation.


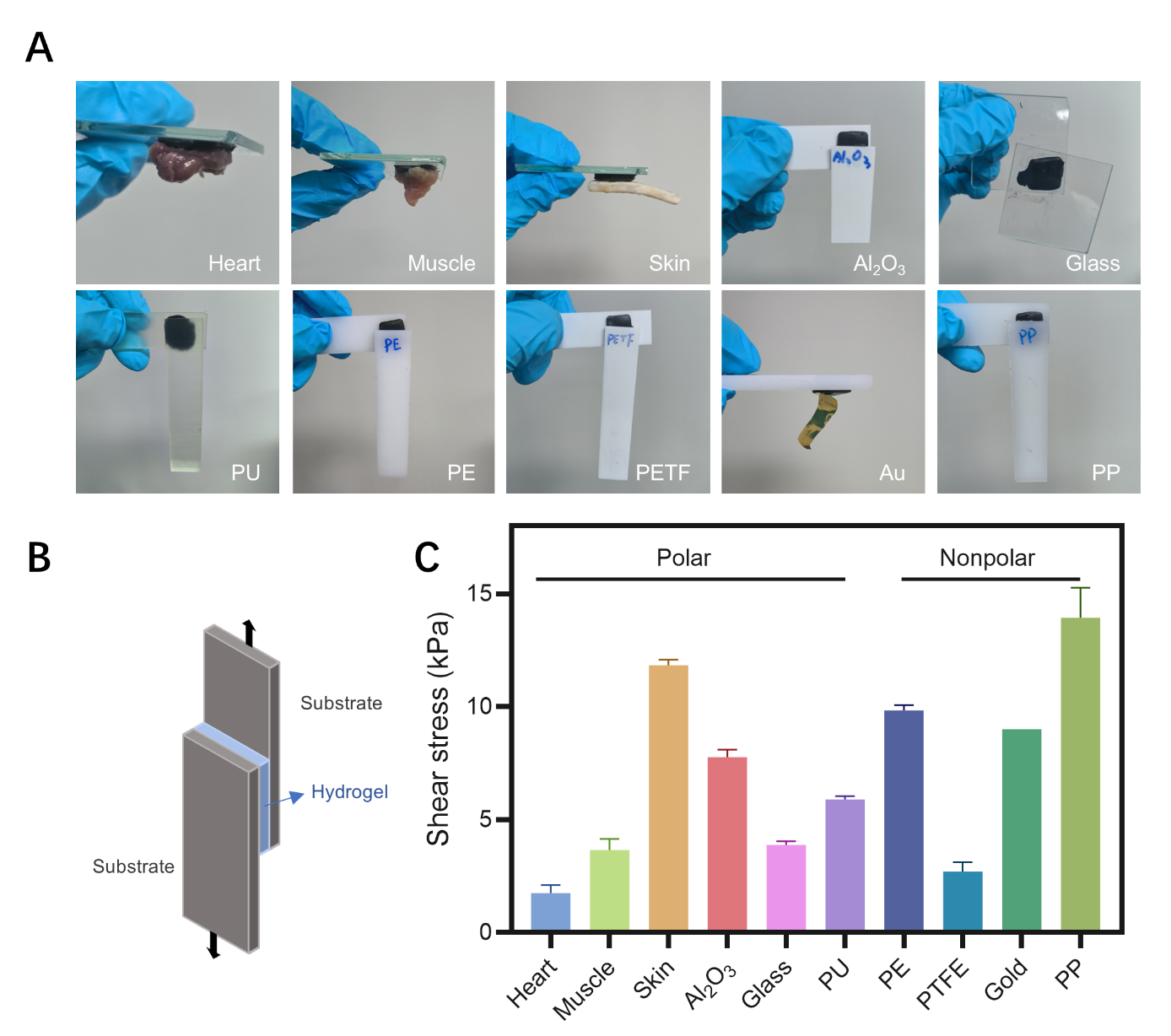


**Figure S26.** Adhesion properties of conducting polymer hydrogel IPNCH@CAPAM. A. Photographs of IPNCH@CAPAM adhered to 10 different polar and nonpolar substrates. B. Schematic illustration of lap shear test. C. The adhesion strength of IPNCH@CAPAM to 10 different substrates.


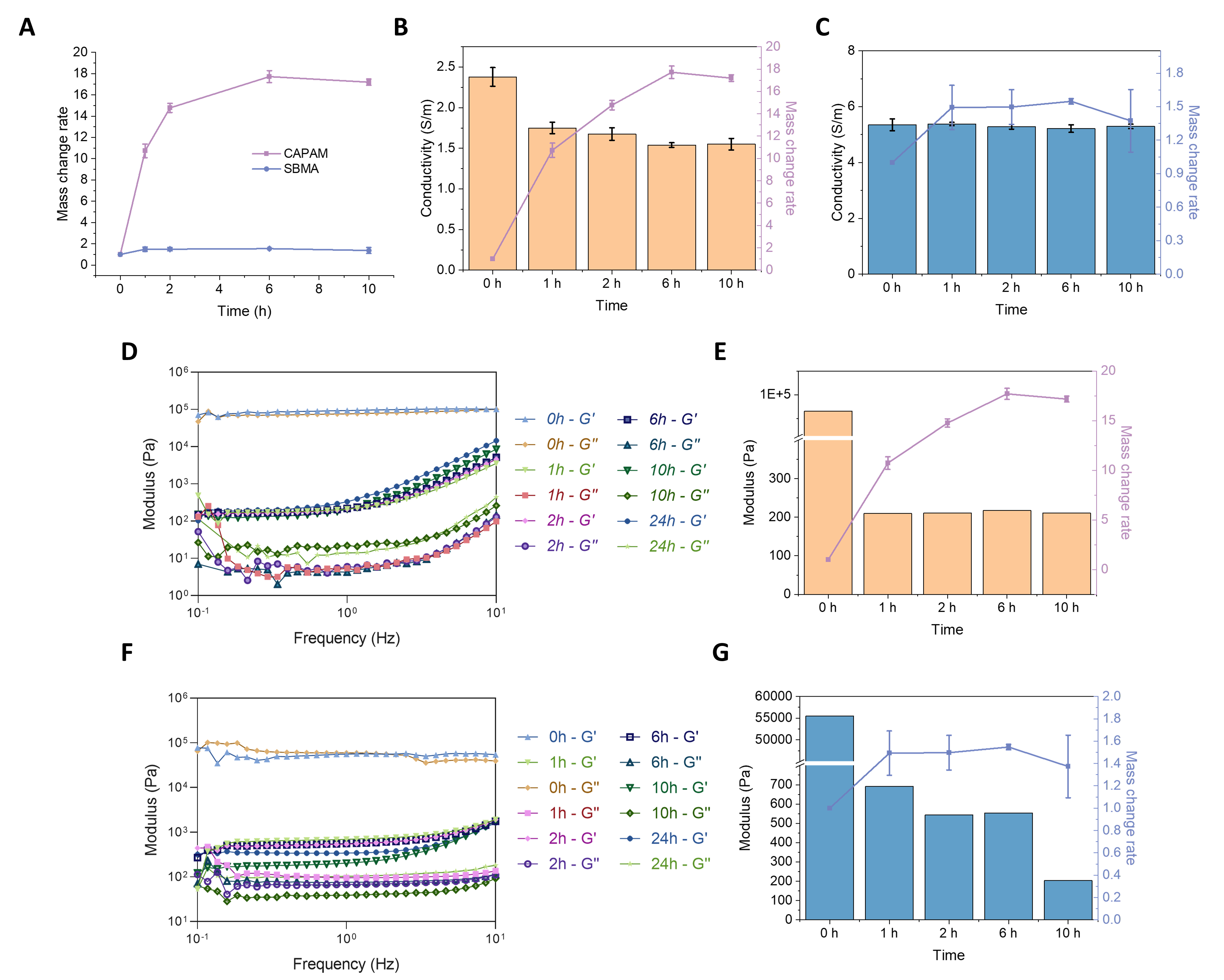


**Figure S27.** The swelling rate measurements over time taken for IPNCH@CAPAM and PSCH@SBMA, and the corresponding modulus and conductivity. A. The relationship between the mass change rate of IPNCH@CAPAM and PSCH@SBMA and the soaking time in PBS. B. Electrical conductivity (bar chart) and mass change rate (dot chart) of IPNCH@CAPAM. C. Electrical conductivity (bar chart) and mass change rate (dot chart) of PSCH@SBMA. D. Relationship between modulus and frequency of IPNCH@CAPAM soaked in PBS for different time. E. the elastic modulus (bar chart) and mass change rate (dot chart) of IPNCH@CAPAM. F. The relationship between the modulus and frequency of PSCH@SBMA soaked in PBS for different times. E. the elastic modulus (bar chart) and mass change rate (dot chart) of PSCH@SBMA.


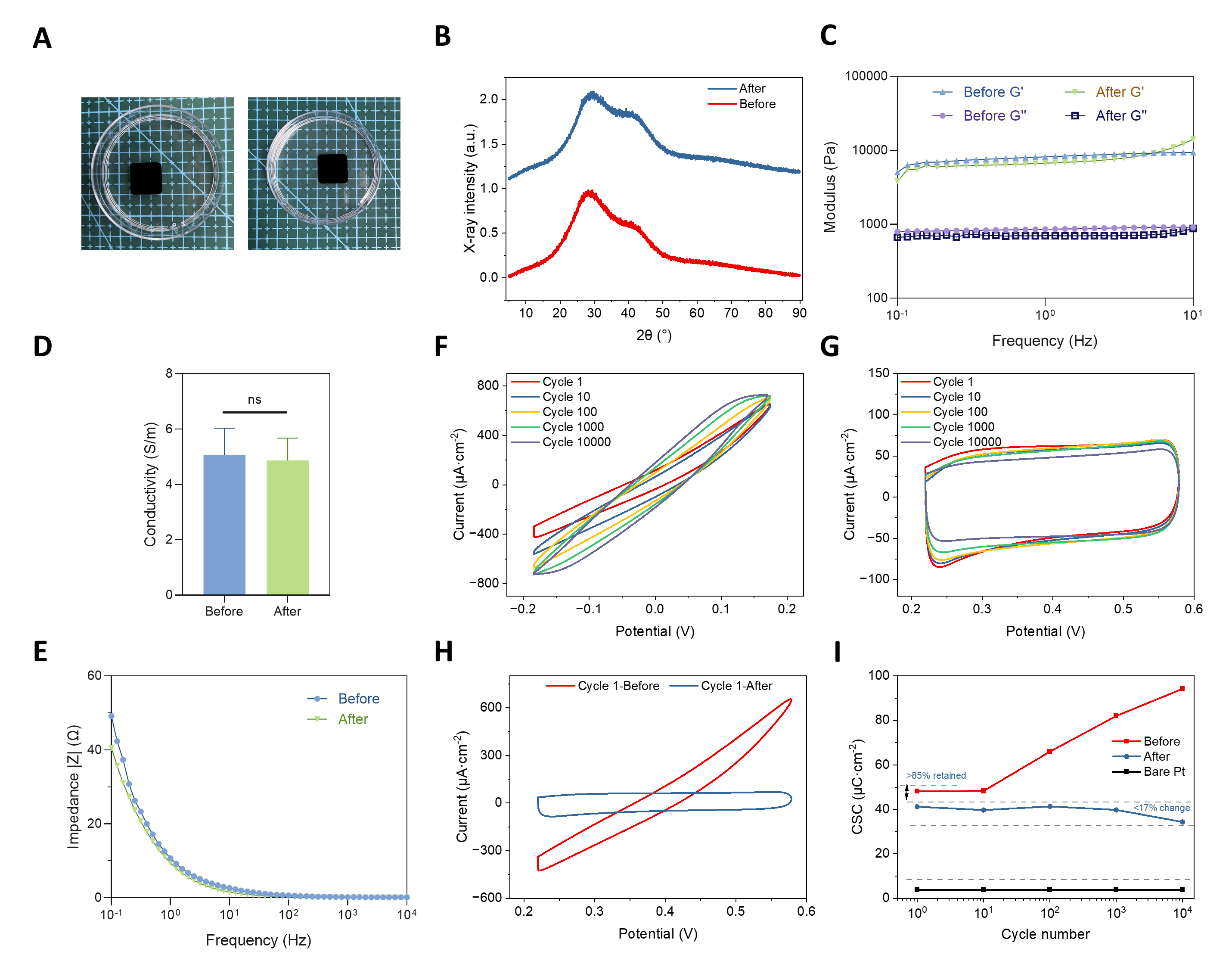


**Figure S28.** Performance of 25 vol.% PCH@EG before and after immersion in DI water or artificial lysosomal fluid for three weeks. A. Optical photos of PCH@EG before and after immersion in DI water. B. X-Ray diffraction (XRD) pattern of PCH@EG. C. Rheological analysis showing the elastic modulus (G') and viscous modulus (G'') of the PCH@EG as a function of frequency (0.1–10 Hz). D. Conductivity of PCH@EG. E. Impedance spectra of PCH@EG over a physiologically relevant frequency range of 0.1–10,000 Hz. F. 10,000 cycles CV curves of PCH@EG swollen in aLF in room temperature. G. 10,000 cycles CV curve after three weeks of immersion of PCH@EG hydrogel in aLF at room temperature. H. Comparison of CV curves of PCH@EG hydrogel before and after immersion in aLF. I. Comparison of CSC changes in PCH@EG hydrogel during 10,000 cycles before and after immersion in aLF.


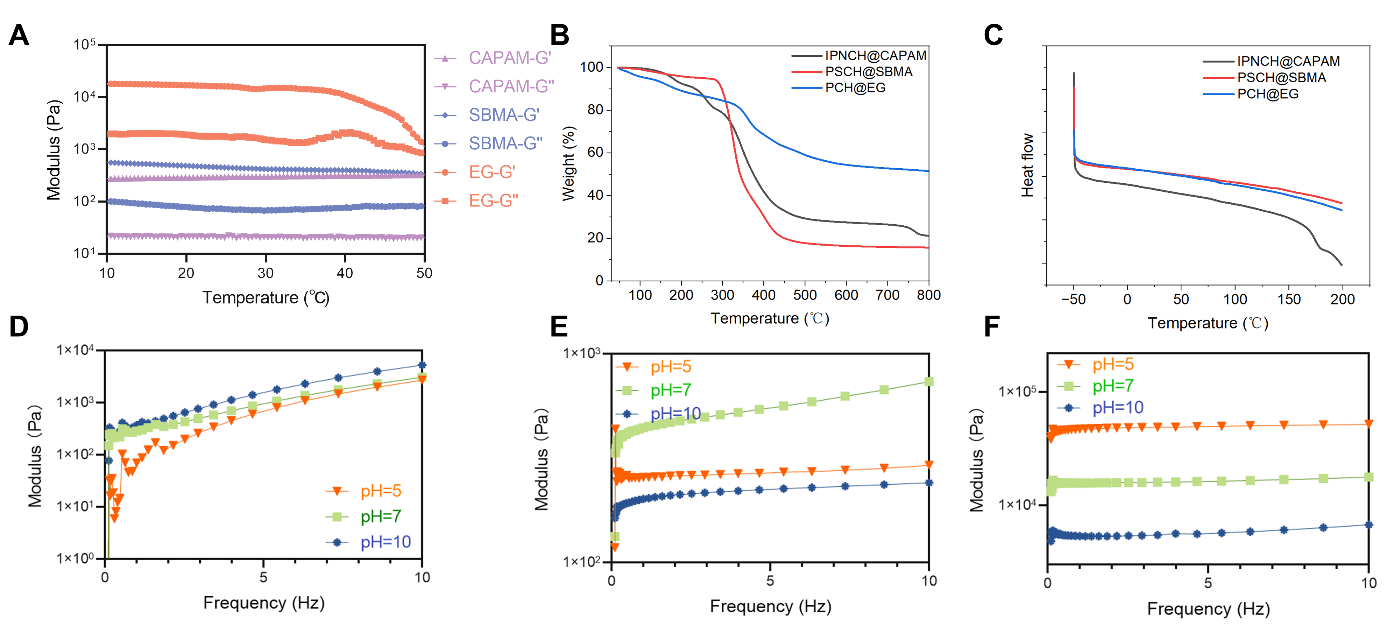


**Figure S29.** Thermal and pH stability of IPNCH@CAPAM, PSCH@SBMA, and PCH@EG. A. Effects of temperature on hydrogels’ modulus. B-C. TGA-DSC Analysis. D-F. Effects of pH on hydrogels’ modulus.

**Notes: Effect of Temperature on Hydrogel**

1) Modulus change

We examined the mechanical properties of the three hydrogels across a temperature range of 4°C to 50°C, using a rheometer to measure both the elastic and viscous moduli. The results show a slight increase in the modulus of IPNCH@CAPAM with rising temperature. This can be attributed to the tighter cross-linked network within the hydrogel, likely due to stronger intermolecular interactions, particularly hydrogen bonding in the carrageenan structure. As temperature increases, these interactions become more pronounced, leading to a more rigid network.

For PSCH@SBMA, the modulus decreased slightly as temperature rose, likely due to the weakening of ionic bonds and interactions at higher temperatures. This causes local relaxation in the cross-linked network, resulting in a reduction in mechanical strength.

In contrast, PCH@EG exhibited a significant decrease in modulus above 40°C, indicating weaker heat resistance compared to the other two hydrogels. This could be due to the separation of PEDOT and PSS segments during the annealing process, which, while improving conductivity, does not create permanent strong interactions. As a result, the network structure becomes more relaxed at elevated temperatures, leading to a reduction in modulus.

Overall, the impact of temperature on hydrogel modulus varies across different hydrogel systems. The IPNCH@CAPAM system exhibits good temperature stability, with a slight increase in modulus as the temperature rises. In contrast, the modulus of the PSCH@SBMA system decreases slightly, likely due to the weakening of ionic interactions at higher temperatures. The PCH@EG system shows a significant drop in modulus, suggesting that its internal network structure is compromised under elevated temperatures.

For physiological applications such as electrical signal recording, which generally occur at room temperature (25°C) or body temperature (37°C), the differences in hydrogel performance due to temperature fluctuations are minimal. However, for broader applications, selecting the appropriate hydrogel system based on the specific temperature conditions is essential to ensure long-term stability.

2) Thermal Stability (TGA-DSC Analysis)

Thermogravimetric analysis (TGA) highlights the mass loss behavior of the three hydrogel systems under different temperatures. For the IPNCH@CAPAM hydrogel, significant mass loss occurs below 200°C, primarily due to the evaporation of absorbed water and the breakdown of low-molecular-weight components. However, beyond this point, the system maintains approximately 20% of its mass even at 450°C, indicating a robust thermal stability at higher temperatures. This suggests that the IPNCH@CAPAM hydrogel retains structural integrity despite partial degradation under extreme heat. In contrast, the PSCH@SBMA hydrogel exhibits faster degradation around 300°C, likely due to the decomposition of ionic groups in the SBMA component. By 500°C, approximately 15% of the hydrogel mass remains, reflecting slightly lower thermal stability than IPNCH@CAPAM, but still demonstrating some level of residual material retention at elevated temperatures. The PCH@EG hydrogel demonstrates the highest thermal stability, with significant mass loss starting only around 400°C. Even at 800°C, substantial material remains, implying that the addition of ethylene glycol (EG) enhances the hydrogel’s stability under extreme conditions.

The differential scanning calorimetry (DSC) curves show the heat flow changes of the hydrogels as they undergo temperature shifts. All three hydrogels exhibit a notable decline in heat flow at approximately -50°C, indicating that their glass transition temperature (Tg) is below this point. This low Tg suggests that the hydrogels remain flexible and stable in colder environments. For IPNCH@CAPAM, the heat flow continues to decrease with rising temperatures, reflecting strong heat absorption and complex thermal behavior influenced by the interaction between different phases of the material. The PSCH@SBMA and PCH@EG hydrogels show stable heat flow curves throughout the temperature range, further supporting their good thermal stability across a broad spectrum of temperatures.

Based on the TGA and DSC analyses, we infer that all three hydrogel systems exhibit excellent thermal stability across a range of temperatures. Their flexibility, demonstrated by the low glass transition temperature, coupled with their thermal stability before decomposition, suggests that these hydrogels can perform effectively in environments ranging from -30°C to 100°C. This temperature window is particularly relevant for applications in biomedicine and flexible electronics, where maintaining mechanical and electrical properties under varying thermal conditions is essential.

Moreover, the high thermal stability of these hydrogels offers potential for use in extreme environments, such as high-temperature applications or conditions involving thermal shock. This durability ensures the long-term safety and lifespan of the materials, making them suitable for a range of practical applications.

**Figure S30.** Impedance of commercial gels in the frequency range 10-1000Hz.


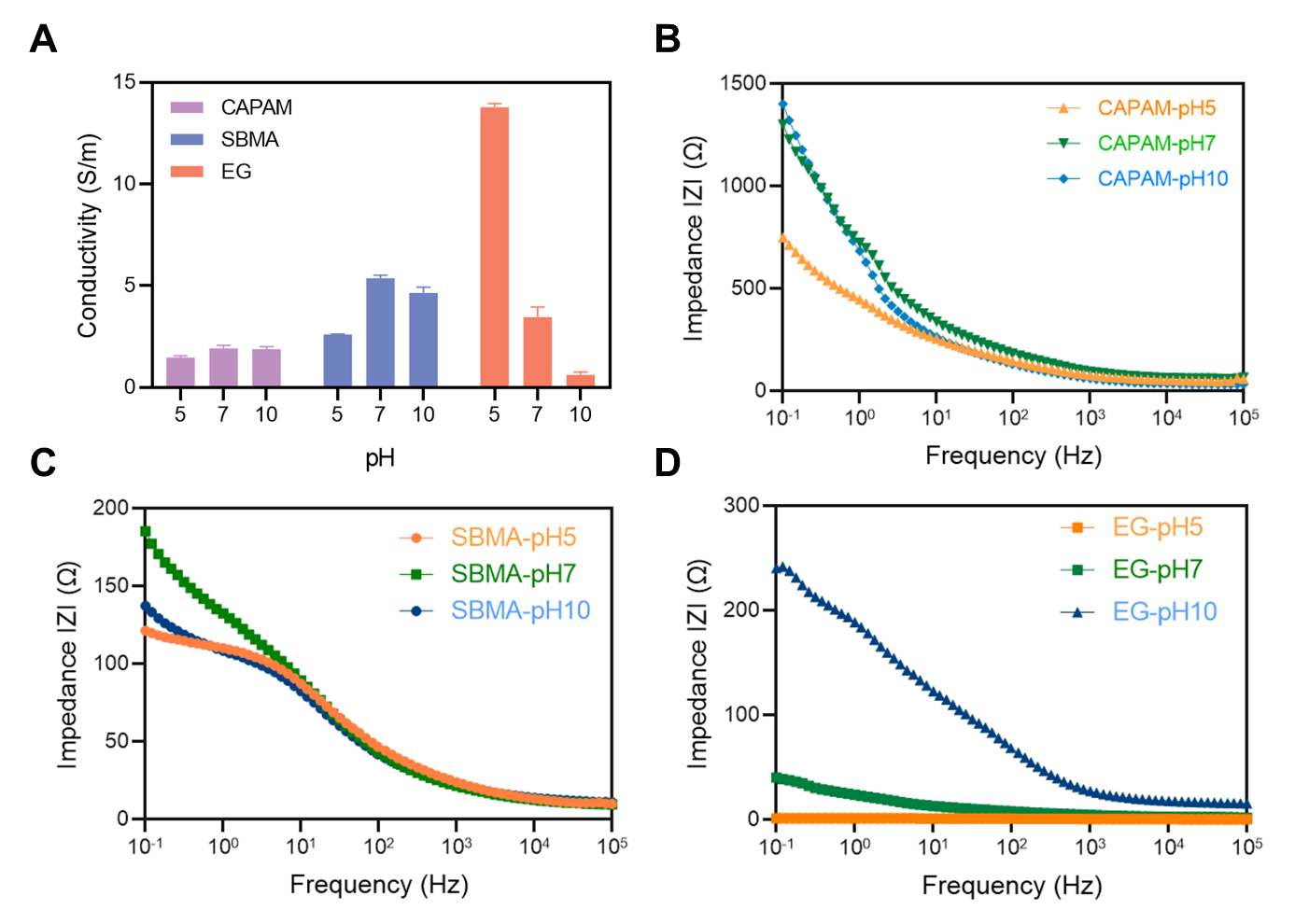


**Figure S31.** Effect of pH on the electrical properties of IPNCH@CAPAM, PSCH@SBMA, and PCH@EG. A. Changes of hydrogels’ conductivity with pH. B-D. Changes of hydrogels’ impedance with pH.

**Notes: Effect of pH on Mechanical and Electrical Properties of Hydrogels**

Considering the application of these hydrogels for epidermal or implantable electrophysiological signal recording, we evaluated their properties under different pH conditions relevant to physiological environments: pH 5 (acidic, similar to skin), pH 7 (neutral, similar to blood), and pH 10 (alkaline, stronger than pancreatic fluid). We tested the mechanical and electrical properties of the hydrogels at these pH levels.

1) Mechanical Properties: We used shear modulus as the indicator of mechanical performance. The IPNCH@CAPAM hydrogel showed minimal change in modulus across the pH range, reflecting its good pH adaptability due to the pH-insensitive carrageenan network structure. The PSCH@SBMA hydrogel displayed the highest modulus at pH 7, with decreases at both pH 5 and pH 10, particularly at pH 10. This indicates that PSCH@SBMA is most stable in neutral conditions, but experiences weakened ionic interactions under acidic or alkaline conditions. The PCH@EG hydrogel exhibited a significant decrease in modulus with increasing pH, likely due to the pH sensitivity of the conductive polymer. At low pH, the increased charge on sulfonic acid groups in PSS leads to greater electrostatic repulsion, affecting network rigidity. At high pH, the repulsion weakens, causing the network to relax and the modulus to decrease. Additionally, Changes in pH can significantly impact the ion distribution within the hydrogels, particularly influencing the dissociation of sulfonic acid groups in PSS under varying pH conditions. This, in turn, affects the swelling behavior of the hydrogels and the interactions at the crosslinking points within the material. As a result, fluctuations in pH can lead to substantial variations in the material's modulus due to the reconstruction of the hydrogel network.

2) Electrical Properties: Conductivity and impedance were measured after full immersion in different pH environments. IPNCH@CAPAM showed relatively stable conductivity across the pH range, with a slight increase from pH 5 to 7 and a minor decrease at pH 10, indicating that the conductive network is largely unaffected by pH changes. PSCH@SBMA exhibited its highest conductivity at neutral pH, consistent with its modulus behavior, suggesting optimal ionic conductivity at pH 7, while the solubility and charge transport path of ionic compounds are destroyed at strong acid or base conditions. In contrast, PCH@EG experienced a significant drop in conductivity at higher pH, likely due to phase separation or disentanglement of PEDOT chains at elevated pH levels, which disrupts the conductive network. The results of impedance change with pH are consistent with the law of conductivity change.

The results demonstrate that the IPNCH@CAPAM hydrogel maintains stable mechanical and electrical properties across a wide pH range, making it suitable for diverse environments. PSCH@SBMA performs best at neutral pH, with performance declining at extreme pH levels. The PCH@EG hydrogel, with its sensitivity to high pH, is not recommended for long-term use in alkaline conditions. Depending on the pH environment, the most appropriate hydrogel system can be selected to ensure stable mechanical and electrical performance.


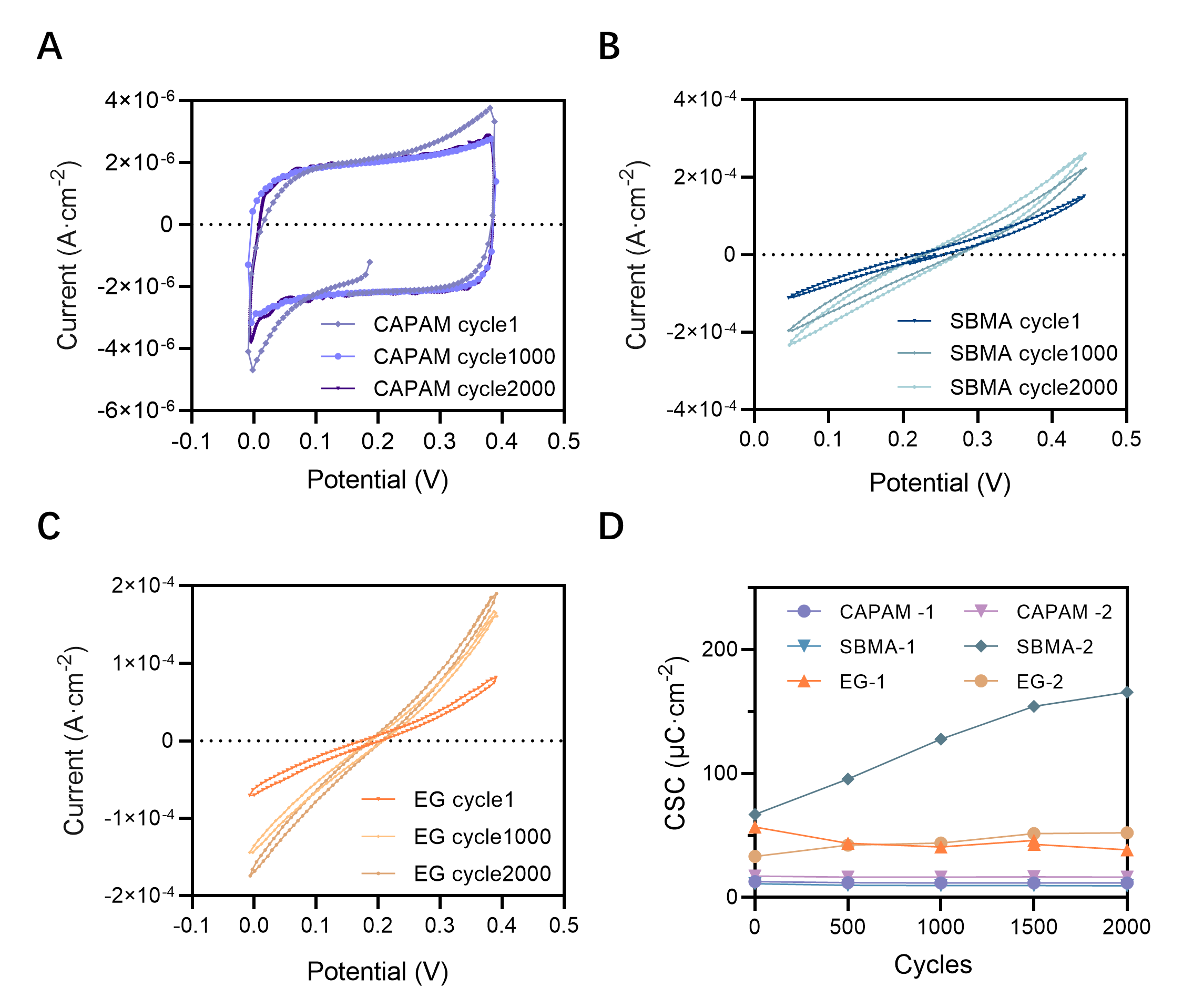


**Figure S32.** Characterization of the time stability of the electrical properties of hydrogels by cyclic voltammetry. A. Cyclic voltammetry curves of IPNCH@CAPAM after soaking water for 50 days in room temperature. B. Cyclic voltammetry curves of PSCH@SBMA after soaking water for 50 days in room temperature. C. Cyclic voltammetry curves of PCH@EG after soaking water for 50 days in room temperature. D. Quantification of charge storage capacity (CSC) across multiple cycles, where, -1 represents the hydrogels 50 days ago, -2 represents the hydrogels after soaking water for 50 days in room temperature.

**Notes: Changes in Electrical Properties Over Time**

We have supplemented our findings with a detailed analysis of the changes in charge storage capacity (CSC) of the hydrogels before and after the 50-day period, as well as a stability test with increasing cycle numbers.

The results indicate that the CSC of the IPNCH@CAPAM hydrogel shows a slight increase after 50 days, remaining stable even after 2000 cycles. This suggests that the hydrogel possesses good long-term electrical stability, with the interpenetrating network effectively maintaining the integrity of the conductive network throughout extended electrochemical cycling.

In contrast, the CSC of the PSCH@SBMA hydrogel increased significantly after 50 days, continuing to rise with an increasing number of cycles. This indicates substantial changes in the conductive network over time and with repeated use. During prolonged operation, the SBMA hydrogels likely undergo ion exchange with their external environment, which activates the ion-conducting network. Additionally, the hydrogel's swelling behavior facilitates greater ionic mobility, forming a continuous conductive pathway within the gel network. This swelling effect is further supported by a significant increase in specific surface area observed after 50 days (Table S1).

The CSC of the PCH@EG hydrogel remained relatively unchanged after 50 days; however, it displayed a decrease in CSC with increasing cycles prior to this period. Interestingly, after 50 days, the CSC began to increase with more cycles, suggesting that the hydrogel gradually established a more stable conductive pathway over time.

The cyclic voltammetry experiments demonstrate that the IPNCH@CAPAM system maintains excellent electrical stability during prolonged use. In contrast, the CSC of the PSCH@SBMA system gradually increases over time, suggesting that its conductive network is being optimized with continued application. Notably, the PCH@EG system exhibits improved electrical performance after 50 days of stability testing. These findings indicate that while the electrical properties of hydrogels can change over time, their long-term stability can be significantly enhanced through the optimization of the conductive network design.


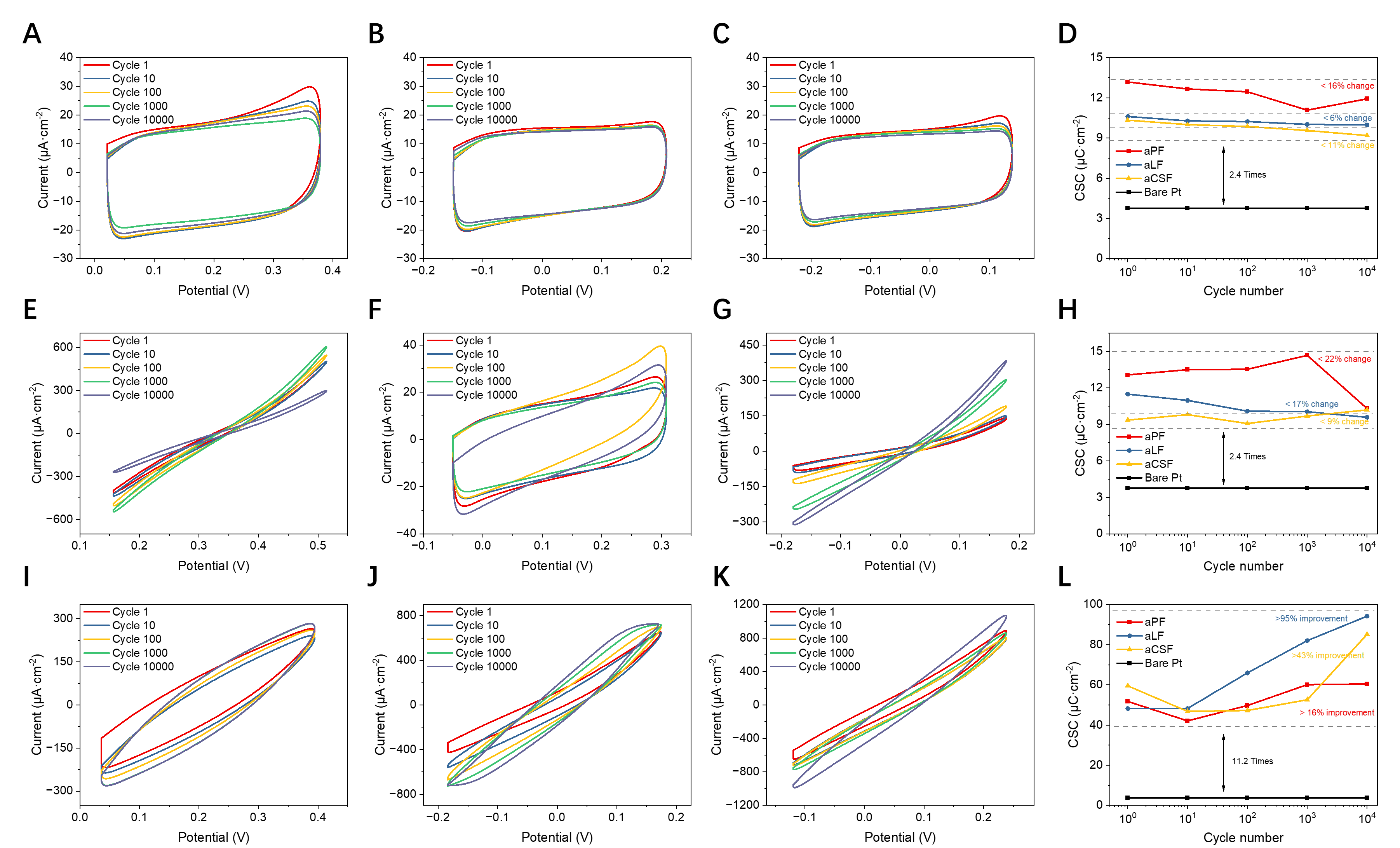


**Figure S33.** Electrochemical properties of the selected hydrogels swollen in artificial perspiration fluid (aPF), artificial lymphatic fluid (aLF), and artificial cerebrospinal fluid (aCSF). A-C. CV curves for the IPNCH@CAPAM swollen in aPF, aLF, and aCSF from 1 to 10,000 cycles. D. CSC against multiple cycling for the IPNCH@CAPAM hydrogel. E-G. CV curves for the PSCH@SBMA swollen in aPF, aLF, and aCSF from 1 to 10,000 cycles. H. CSC against multiple cycling for the PSCH@SBMA hydrogel. I-K. CV curves for the PCH@EG swollen in aPF, aLF, and aCSF from 1 to 10,000 cycles. L. CSC against multiple cycling for the PCH@EG hydrogel.

Notes:

The CV curves of IPNCH@CAPAM swollen in aPF, aLF, and aCSF exhibited stable electrochemical performance, with a slight decrease in CSC within 20% after 10,000 cycles, indicating that it can maintain the integrity of the conductive network after multiple cycles.

The CV curves of PSCH@SBMA hydrogels showed a degree of attenuation with increasing cycles, indicative of a downward trend in CSC. This is attributed to the electrostatic shielding effect of the zwitterionic SBMA in the PSCH@SBMA hydrogel, which leads to structural contraction or fracture of the PEDOT:PSS segments during prolonged electrochemical cycling, resulting in the deterioration of electrochemical performance with cycling.

PCH@EG hydrogels exhibited unique electrochemical behavior during the CV cycling process: CSC significantly increased with the number of cycles, particularly noticeable in aLF and aCSF. This anomalous phenomenon could be attributed to the full exposure of PEDOT:PSS in the pure conducting polymer hydrogel, allowing for more free reorganization of the PEDOT chains under the electric field during multiple cycles. This molecular reorganization effect increases the degree of order of the PEDOT segments, enhances the continuity of the conductive channels, and allows for a significant increase in CSC even after prolonged cycling.

Overall, the cycling stability of the three hydrogels under aPF swelling conditions was slightly lower than that under aLF and aCSF swelling: The aPF, due to its acidic pH, may affect the doping state and charge transfer capability of the conductive polymer. Additionally, the higher ion concentration in aPF may induce an ionic screening effect, thereby reducing the ionic permeability and charge transfer efficiency of the conductive polymer. In contrast, the aLF and aCSF in a neutral environment contain bicarbonate and glucose, which have a good buffering effect and are more conducive to maintaining the structural stability and electrochemical performance of PEDOT:PSS.


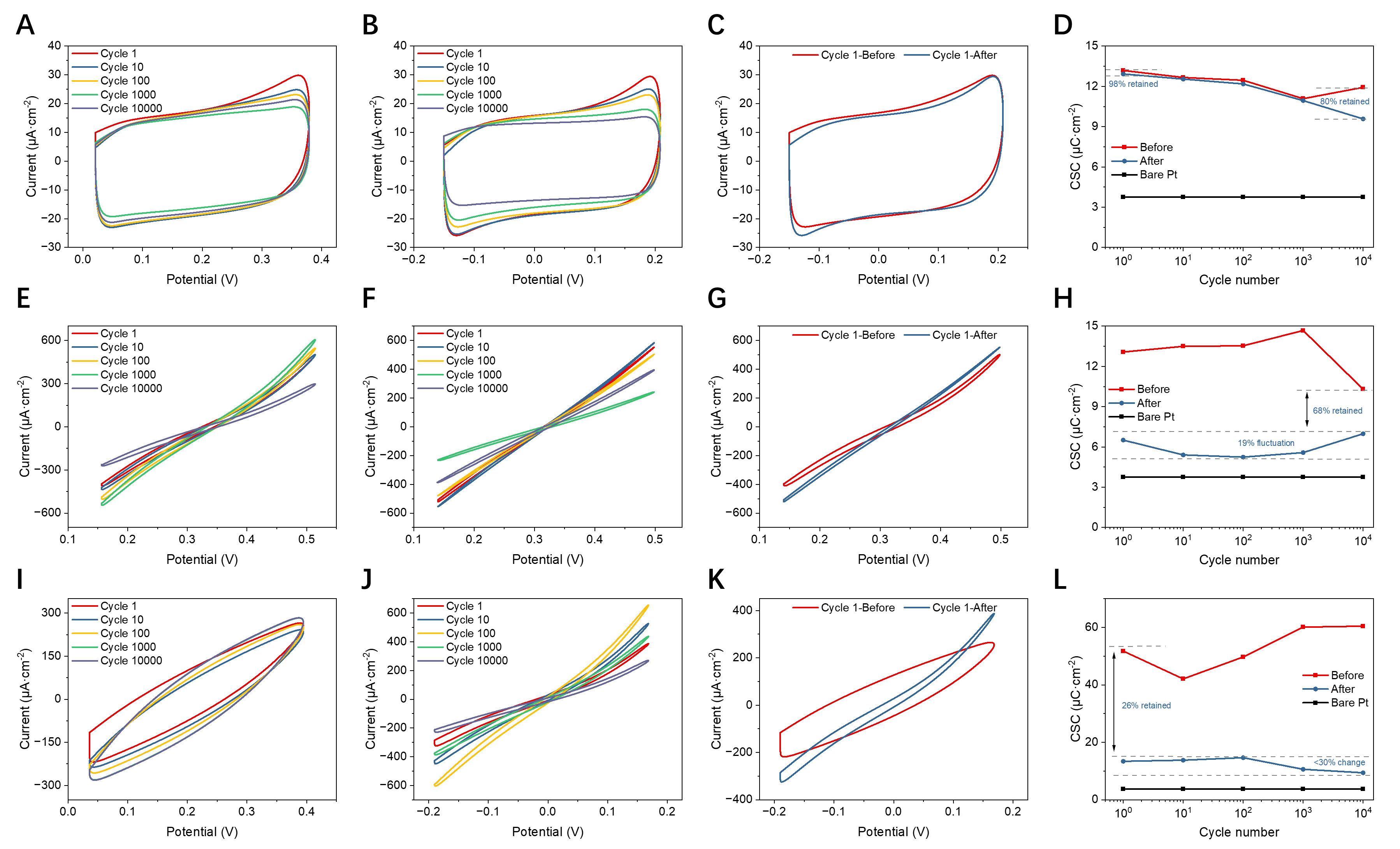


**Figure S34.** Characterization of the long-term stability of the electrochemistry properties of hydrogels by cyclic voltammetry. A. 10,000 cycles CV curves of IPNCH@CAPAM swollen in aPF in room temperature. B. 10,000 cycles CV curve after three weeks of immersion of IPNCH@CAPAM hydrogel in aPF at room temperature. C. Comparison of CV curves of IPNCH@CAPAM hydrogel before and after immersion in aPF. D. Comparison of CSC changes in IPNCH@CAPAM hydrogel during 10000 cycles before and after immersion in aPF. E. 10,000 cycles CV curves of PSCH@SBMA swollen in aPF in room temperature. F. 10,000 cycles CV curve after three weeks of immersion of PSCH@SBMA hydrogel in aPF at room temperature. G. Comparison of CV curves of PSCH@SBMA hydrogel before and after immersion in aPF. H. Comparison of CSC changes in PSCH@SBMA hydrogel during 10,000 cycles before and after immersion in aPF. I. 10,000 cycles CV curves of PCH@EG swollen in aPF in room temperature. J. 10,000 cycles CV curve after three weeks of immersion of PCH@EG hydrogel in aPF at room temperature. K. Comparison of CV curves of PCH@EG hydrogel before and after immersion in aPF. L. Comparison of CSC changes in PCH@EG hydrogel during 10,000 cycles before and after immersion in aPF.


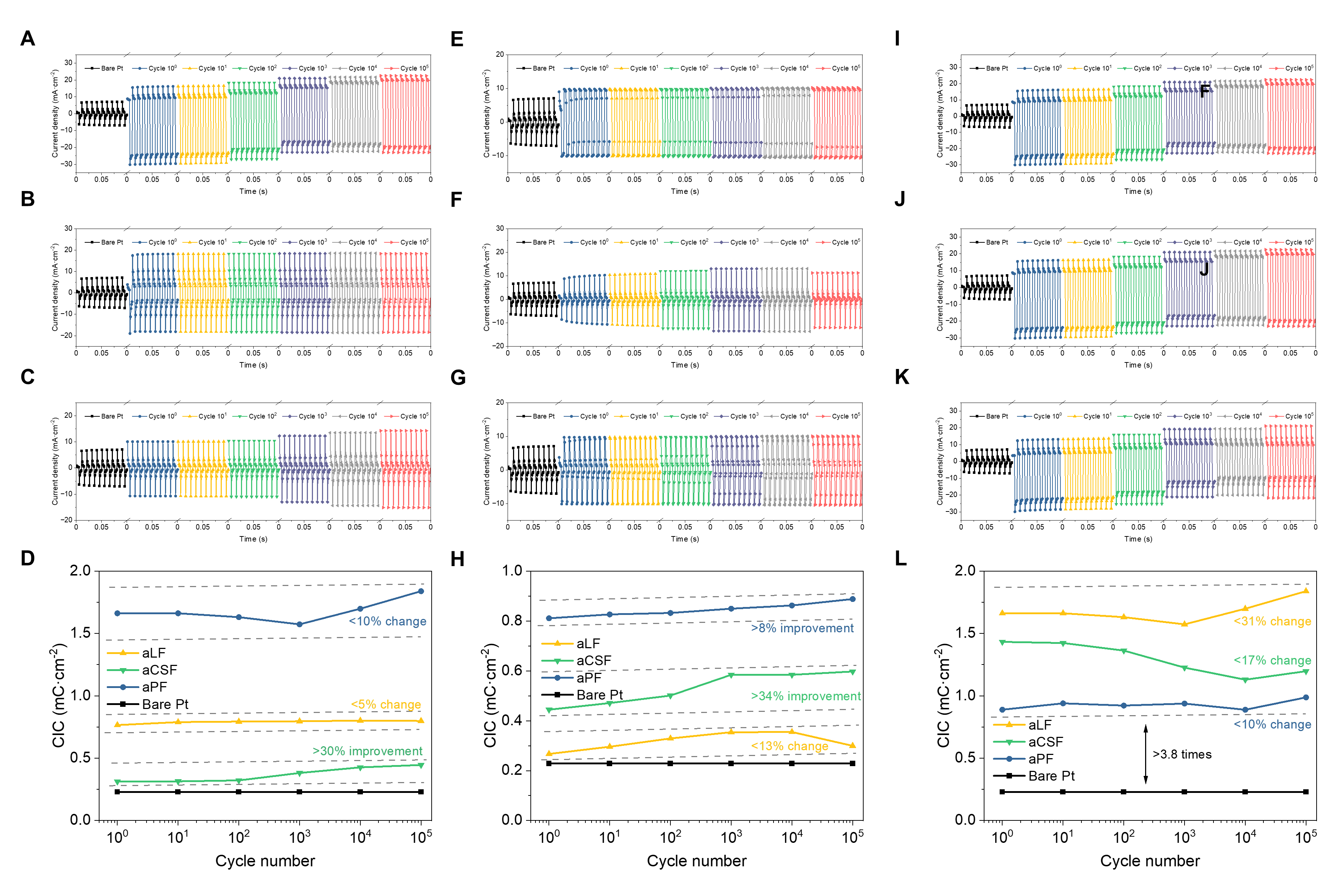


**Figure S35.** The charge injection capacity of the selected hydrogels swollen in artificial perspiration fluid (aPF), artificial lymphatic fluid (aLF), and artificial cerebrospinal fluid (aCSF). A-C) Current density vs time curves of biphasic input pulse test for the IPNCH@CAPAM swollen in aPF, aLF, and aCSF from 1 to 100,000 cycles. D) CIC against multiple cycling for the IPNCH@CAPAM hydrogel. E-G) Current density vs time curves of biphasic input pulse test for the PSCH@SBMA swollen in aPF, aLF, and aCSF from 1 to 100,000 cycles. H) CIC against multiple cycling for the PSCH@SBMA hydrogel. I-K) Current density vs time curves of biphasic input pulse test for the PCH@EG swollen in aPF, aLF, and aCSF from 1 to 100,000 cycles. L) CIC against multiple cycling for the PCH@EG hydrogel.


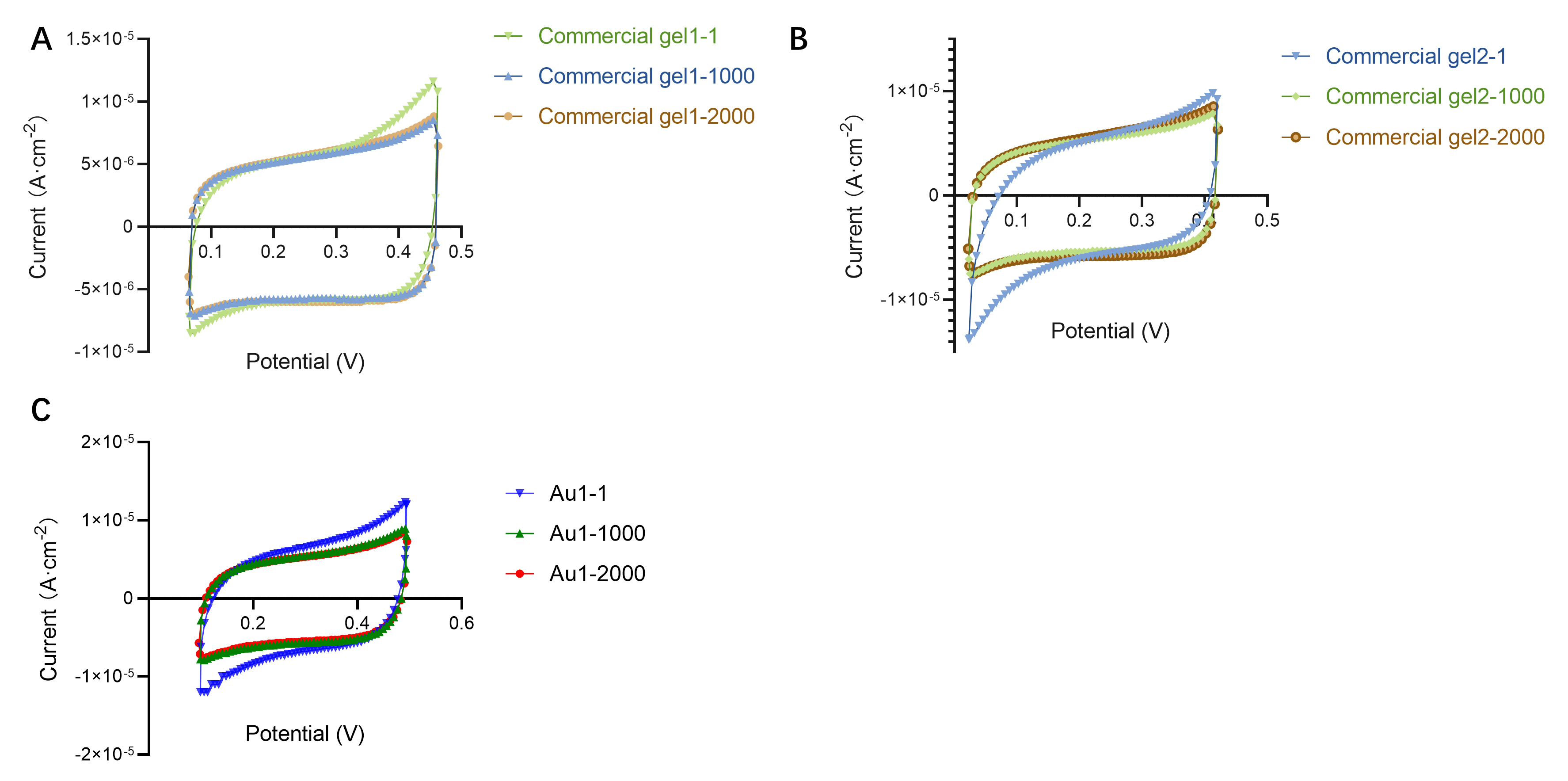


**Figure S36.** Cyclic voltammetry curves of commercial gel and metal electrode for 2000 cycles. A. Cyclic voltammetry curves of commercial gel. B. Cyclic voltammetry curves of commercial gel after 50 days at room temperature. C. Cyclic voltammetry curves of metal electrode.


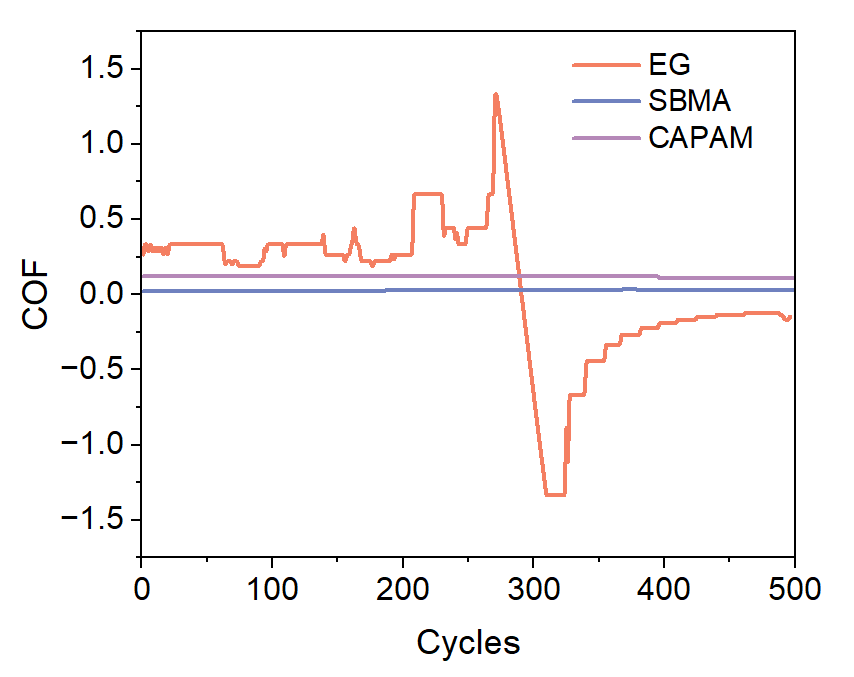


**Figure S37.** Friction coefficient of IPNCH@CAPAM, PSCH@SBMA, and PCH@EG.

**Notes: Changes in Friction Coefficient with Repeated Cycles**

The results indicate that the IPNCH@CAPAM and PSCH@SBMA systems exhibit relatively stable performance during friction testing, demonstrating good friction resistance under specific sliding conditions. However, the PSCH@SBMA system has a low friction coefficient, approaching zero. This characteristic may lead to excessive sliding in vivo, potentially compromising its ability to maintain stable fixation. Consequently, while PSCH@SBMA is not ideal for applications requiring a high coefficient of friction, it may be suitable for low-friction applications, such as external sliding interfaces or self-lubricating surfaces.

In contrast, the PCH@EG system displayed significant fluctuations after prolonged use due to its relatively loose internal molecular network, making it unsuitable for applications involving internal friction. This observation aligns with our conclusion that PCH@EG is not suitable for implantable applications due to its weak modulus and low toughness.


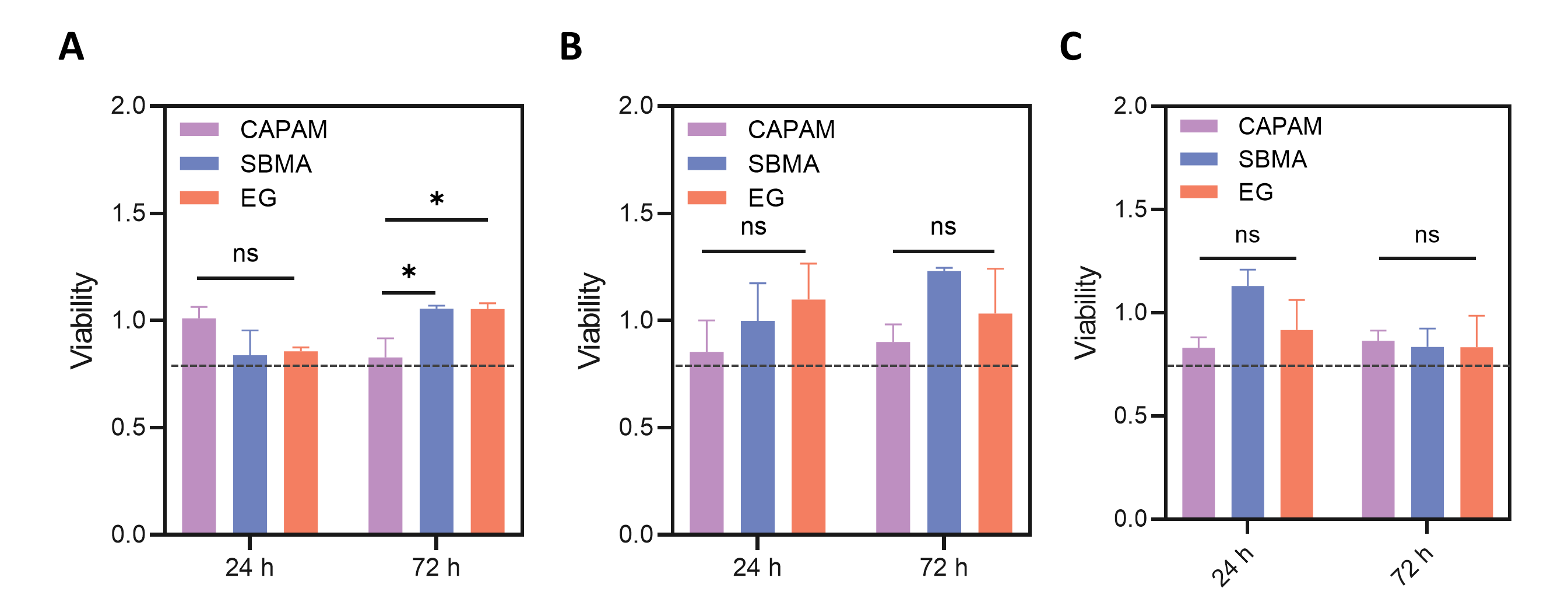


**Figure S38.** Cell viability of cells cultured in hydrogel extraction medium for 24h and 72h. A. Cell viability of BHK21 cells cultured in hydrogel extraction medium for 24h and 72h. B. Cell viability of HEK293T cells cultured in hydrogel extraction medium for 24h and 72h. C. Cell viability of HUVEC cells cultured in hydrogel extraction medium for 24h and 72h. Data are mean ± SD (n=4). *p<0.05 (one-way ANOVA with Tukey’s test). The dashed line indicates the 80% biocompatibility threshold.


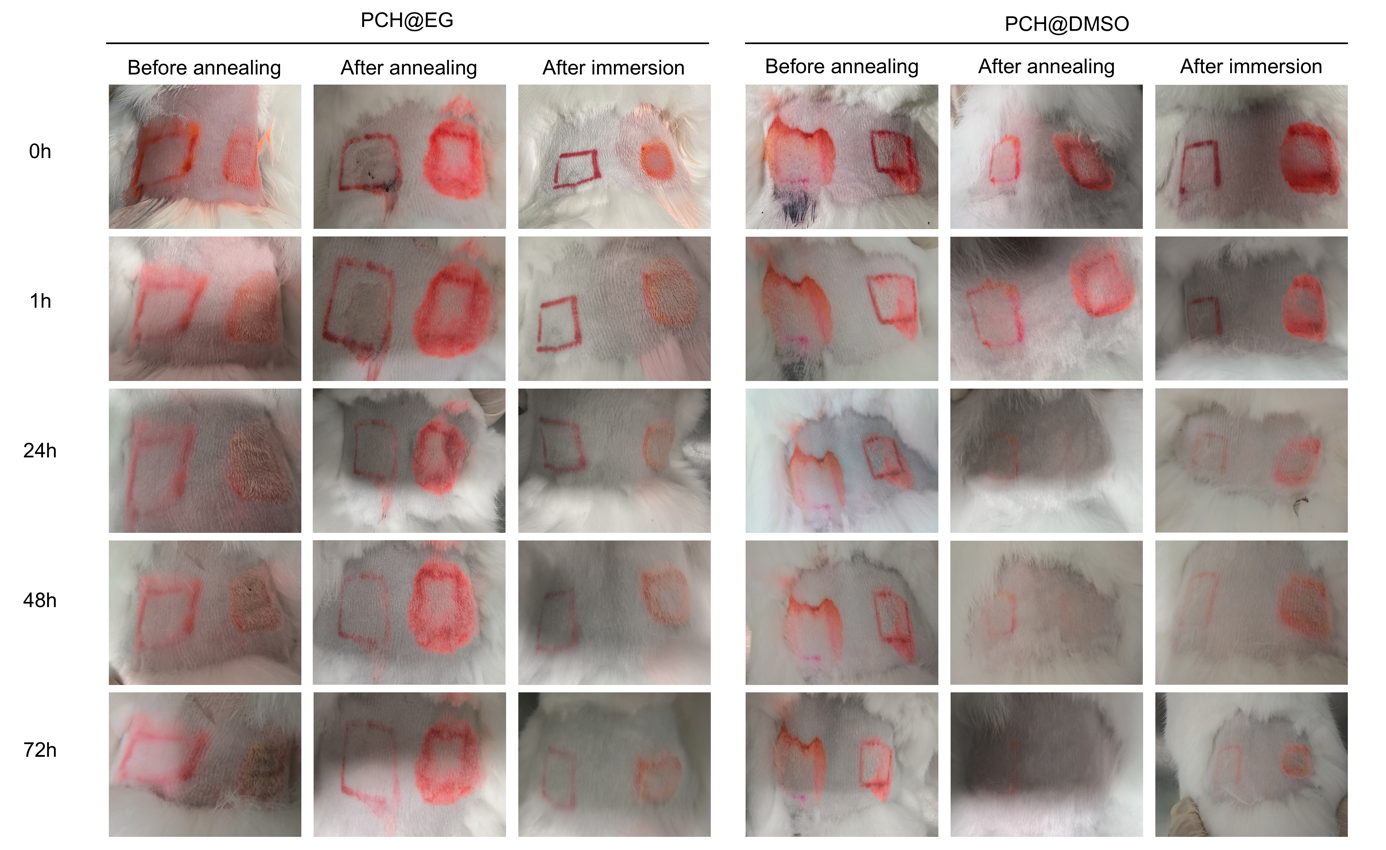


**Figure S39.** Optical photos of the rabbit skin irritation experiments with PCH@EG and PCH@DMSO.


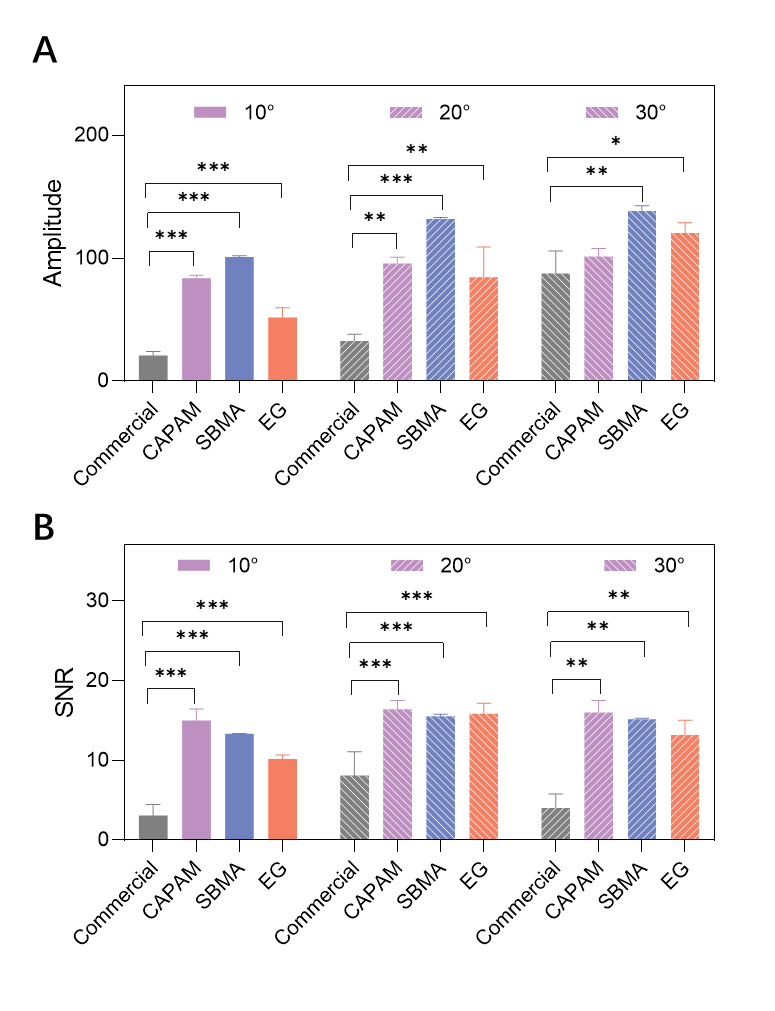


**Figure S40.** Statistical significance analysis of EMG data generated by palm lifting at different angles. A. Signal amplitude. B. Signal to noise ratio (SNR).


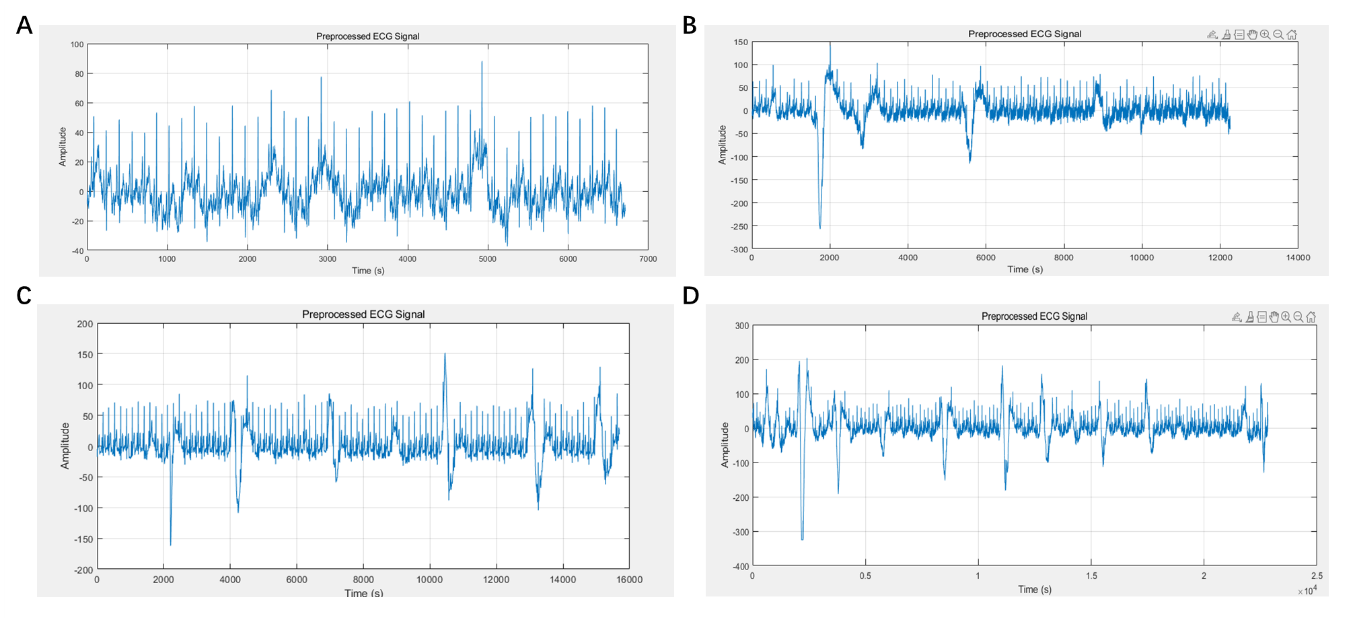


**Figure S41.** Preprocessed epidermal ECG signals recorded by hydrogel electrodes and commercial gel electrode after noise reduction treatment. A. Preprocessed epidermal ECG signals recorded by commercial gel electrode. B. Preprocessed epidermal ECG signals recorded by IPNCH@CAPAM. C. Preprocessed epidermal ECG signals recorded by PSCH@SBMA. D. Preprocessed epidermal ECG signals recorded by PCH@EG.


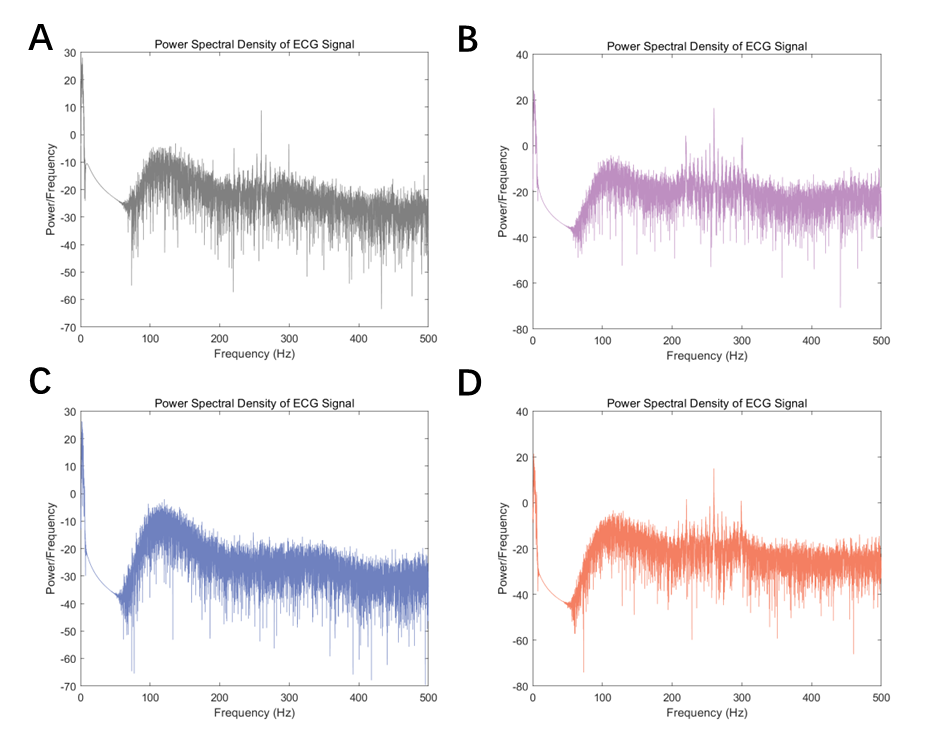


**Figure S42.** Power spectral density of epidermal ECG signals recorded by hydrogel electrodes and commercial gel electrode. A. Power spectral density of epidermal ECG signals recorded by commercial gel electrode. B. Power spectral density of epidermal ECG signals recorded by IPNCH@CAPAM. C. Power spectral density of epidermal ECG signals recorded by PSCH@SBMA. D. Power spectral density of epidermal ECG signals recorded by PCH@EG.


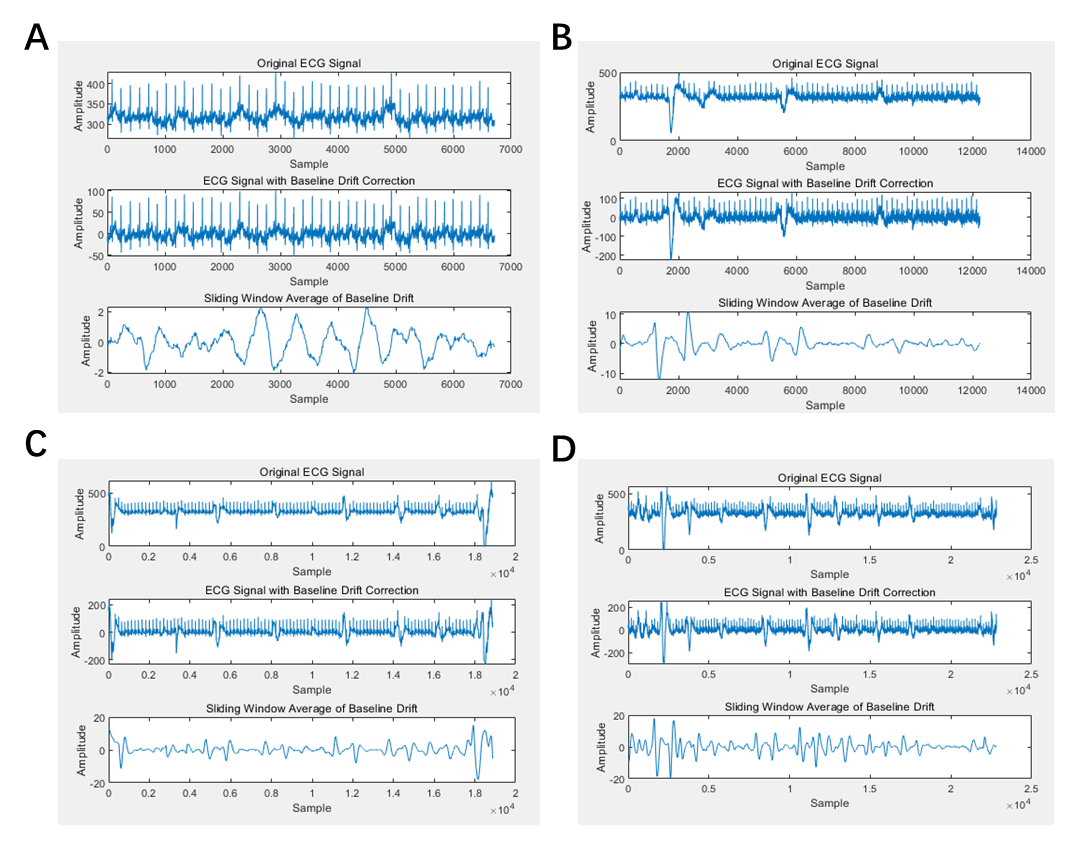


**Figure S43.** Baseline drift of epidermal ECG signals recorded by hydrogel electrodes and commercial gel electrode. A. Original epidermal ECG signals, epidermal ECG signals with baseline drift correction, and sliding window average of baseline drift of epidermal ECG signals recorded by commercial gel electrode. B. Original epidermal ECG signals, epidermal ECG signals with baseline drift correction, and sliding window average of baseline drift of epidermal ECG signals recorded by IPNCH@CAPAM. C. Original epidermal ECG signals, epidermal ECG signals with baseline drift correction, and sliding window average of baseline drift of epidermal ECG signals recorded by PSCH@SBMA. D. Original epidermal ECG signals, epidermal ECG signals with baseline drift correction, and sliding window average of baseline drift of epidermal ECG signals recorded by PCH@EG.


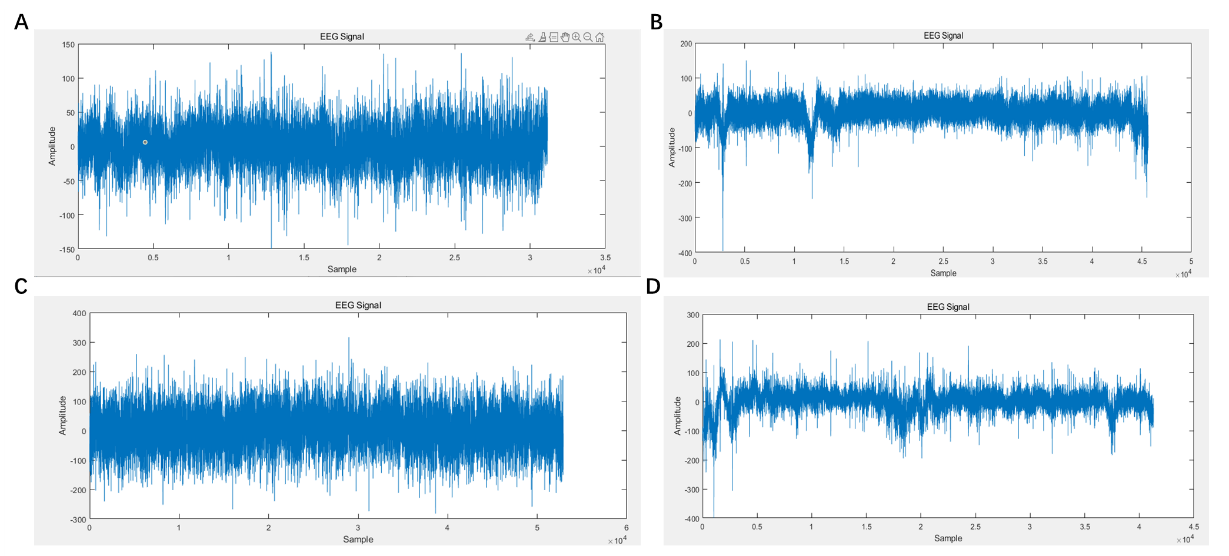


**Figure S44.** Epidermal EEG signals recorded by hydrogel electrodes and commercial gel electrode. A. Epidermal EEG signals recorded by commercial gel electrode. B. Epidermal EEG signals recorded by IPNCH@CAPAM. C. Epidermal EEG signals recorded by PSCH@SBMA. D. Epidermal EEG signals recorded by PCH@EG.


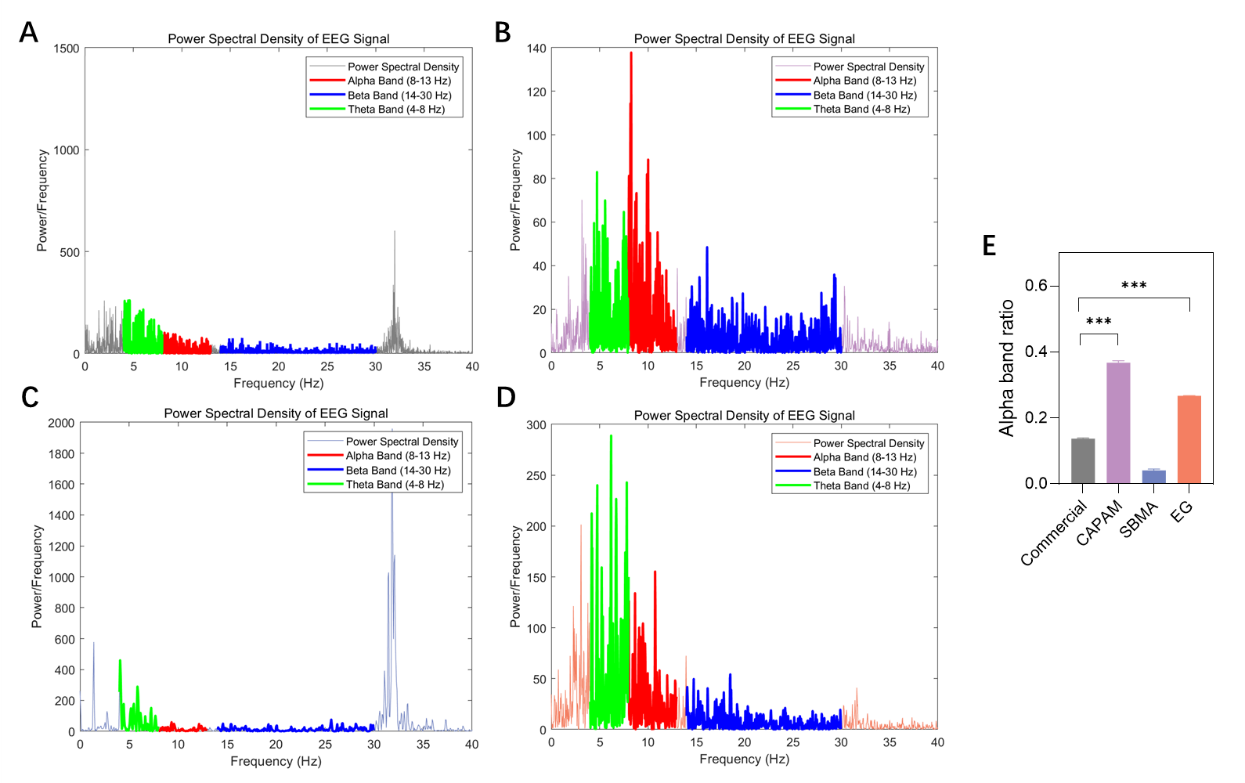


**Figure S45.** Power spectral density of epidermal EEG signals recorded by hydrogel electrodes and commercial gel electrode, where the alpha band was labeled red, the beta band was labeled blue, and the theta band was labeled green. A. Power spectral density of epidermal ECG signals recorded by commercial gel electrode. B. Power spectral density of epidermal ECG signals recorded by IPNCH@CAPAM. C. Power spectral density of epidermal ECG signals recorded by PSCH@SBMA. D. Power spectral density of epidermal ECG signals recorded by PCH@EG. E. Ratio of alpha bands in epidermal EEG signals recorded by hydrogel electrodes and commercial gel electrodes.


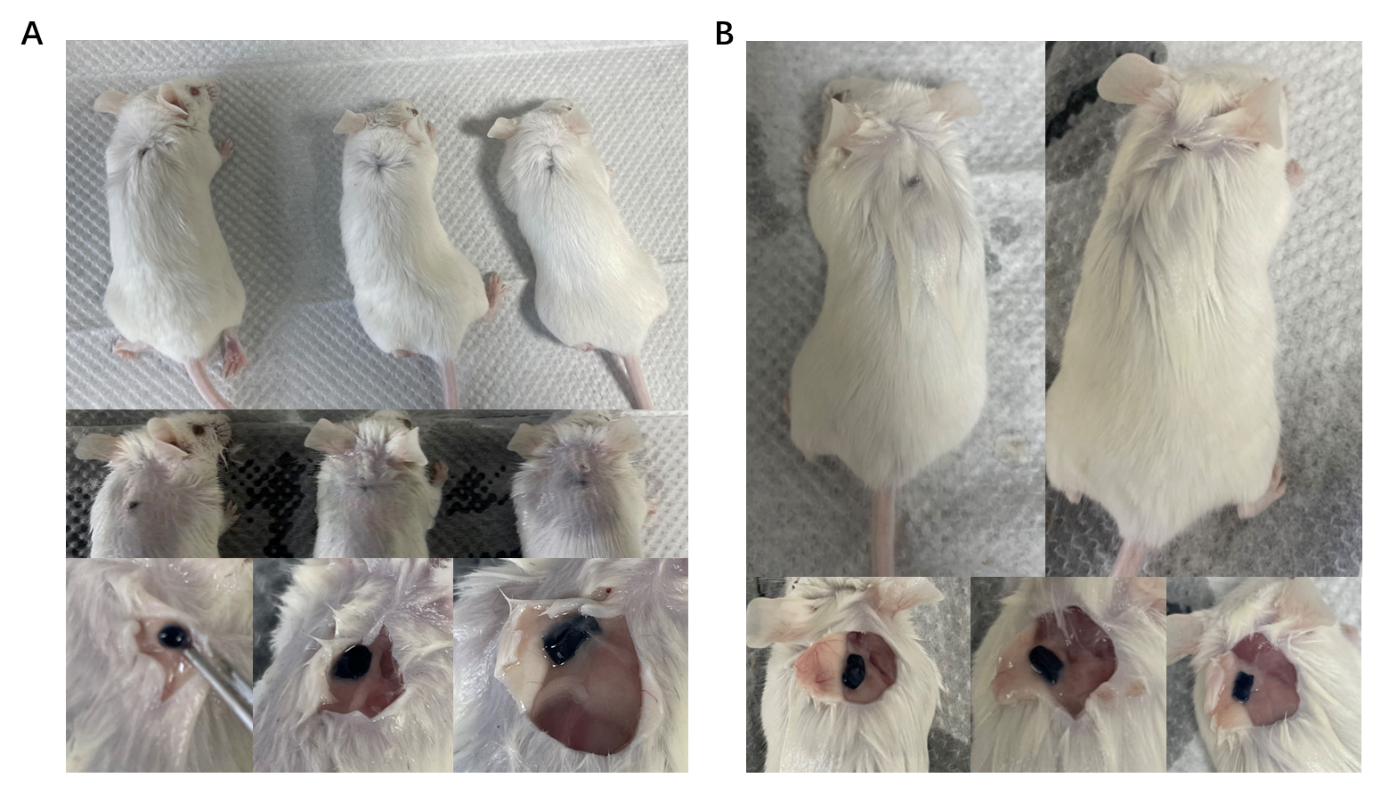


**Figure S46.** Optical images of inflammation in mice implanted subcutaneous hydrogel IPNCH@CAPAM. A. Optical images of inflammation in mice implanted subcutaneous hydrogel IPNCH@CAPAM for 1 week. B. Optical images of inflammation in mice implanted subcutaneous hydrogel IPNCH@CAPAM for 2 weeks.


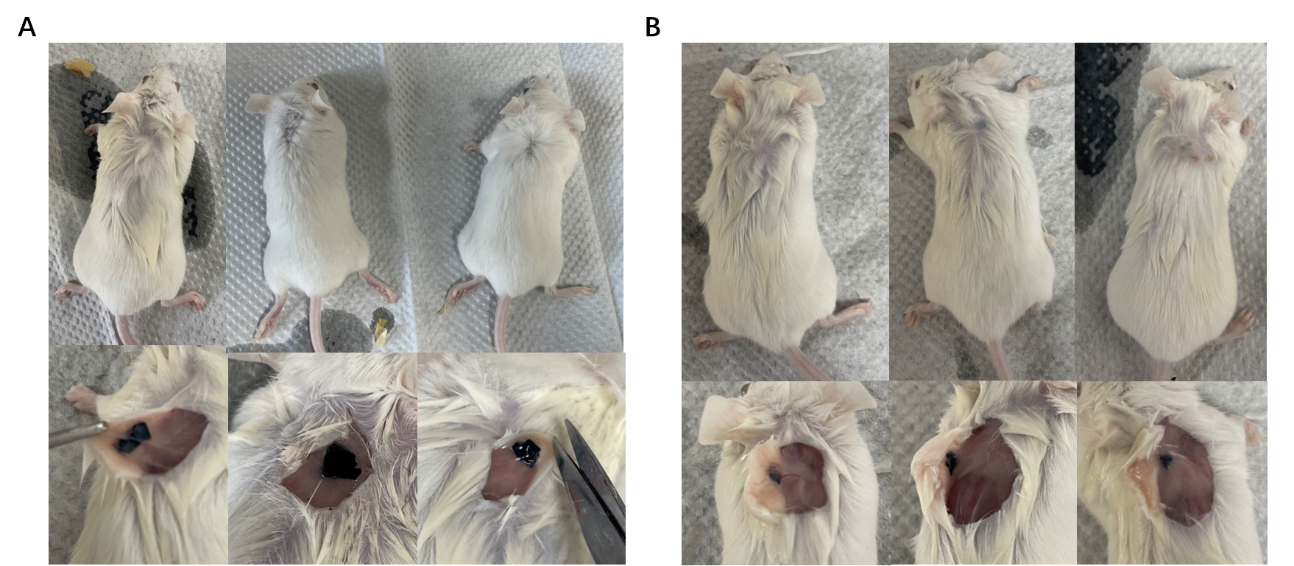


**Figure S47.** Optical images of inflammation in mice implanted subcutaneous hydrogel PSCH@SBMA. A. Optical images of inflammation in mice implanted subcutaneous hydrogel PSCH@SBMA for 1 week. B. Optical images of inflammation in mice implanted subcutaneous hydrogel PSCH@SBMA for 2 weeks.

**
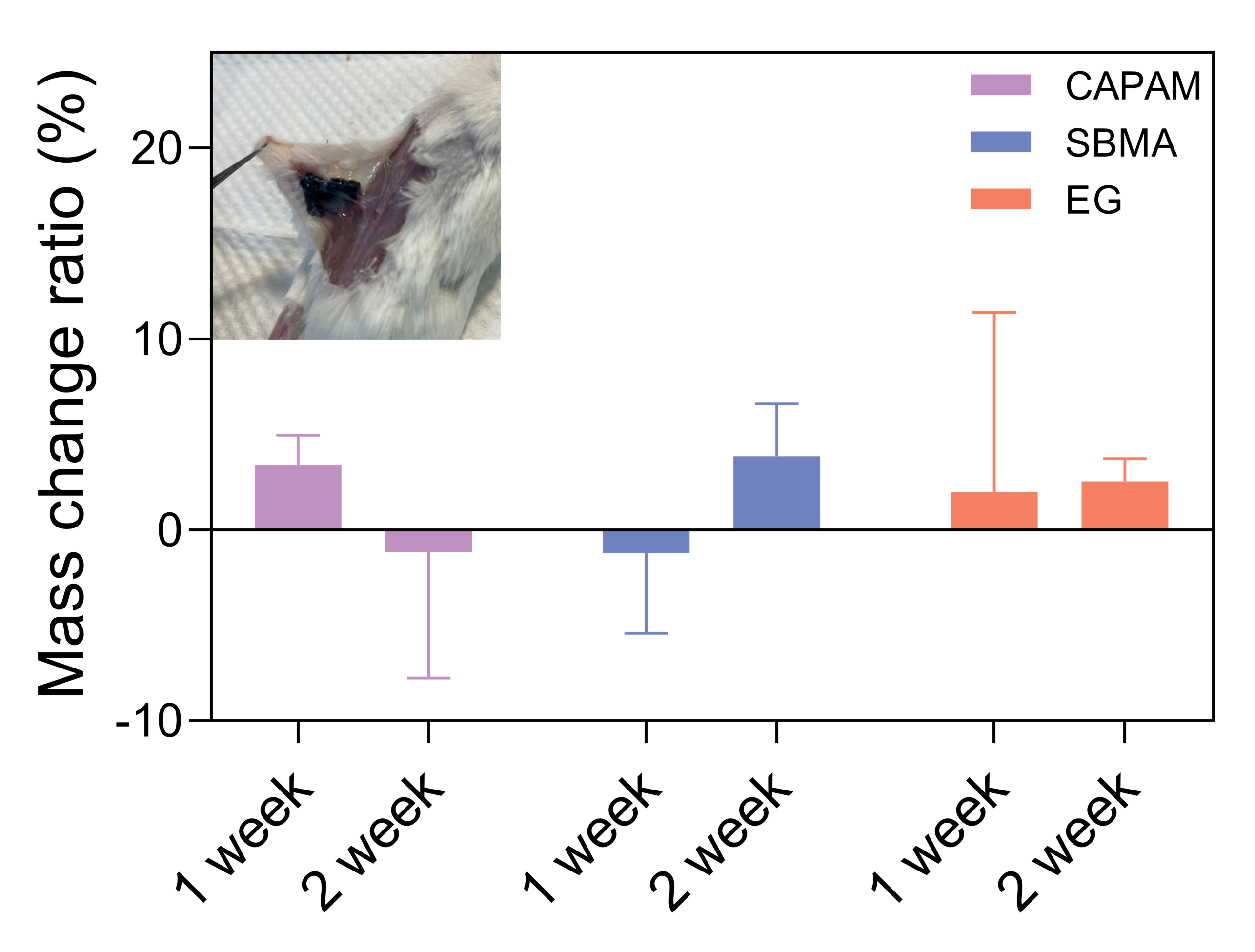
**

**Figure S48.** Mass change ratio of hydrogels after one and two weeks of subcutaneous implantation in mice.


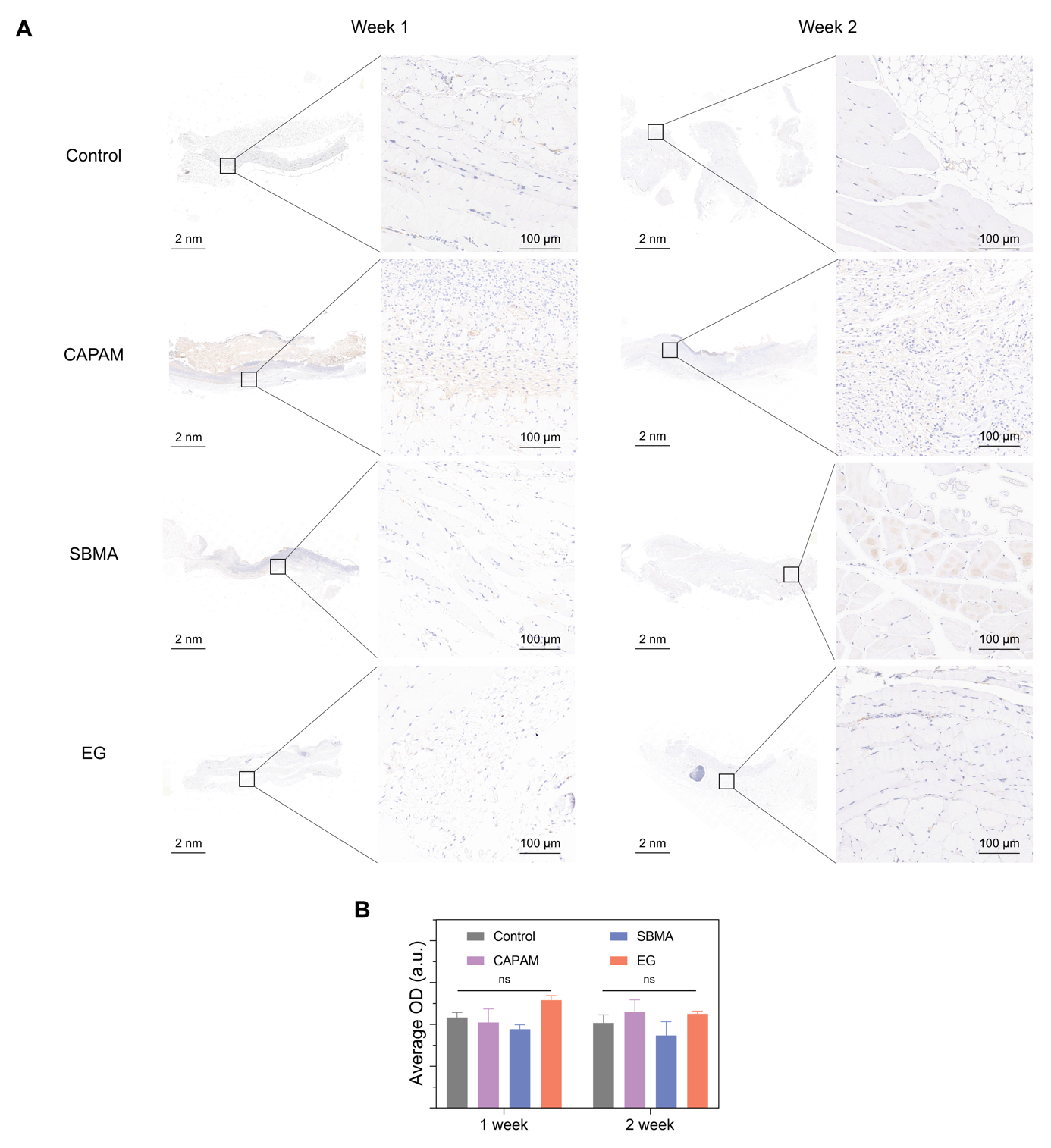


**Figure S49.** The immunohistochemical results of CD68 after subcutaneous implantation of hydrogels. A. Representative immunohistochemistry images of CD68 with or without hydrogels after mouse subcutaneous implantation for 7 and 14 days, respectively. B. Quantitative analysis of the average OD value in the positive area.


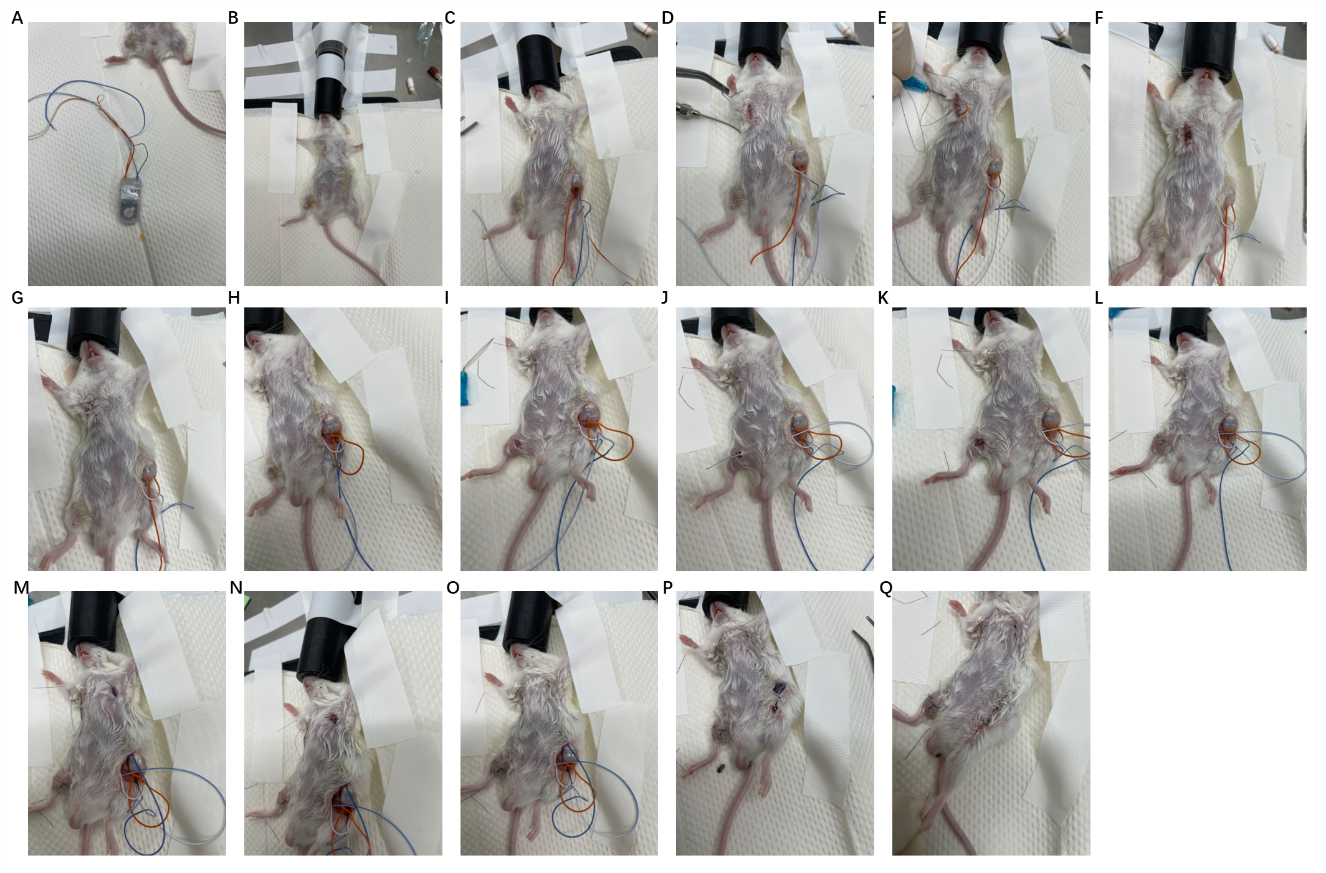


**Figure S50.** The surgical procedure for the ultra-small animal specific HD-X02 implant in mice. A. Optical image of the HD-X02 implant for implantable ECG acquisition and monitoring. B. After anesthesia, the mice were connected to the anesthesia machine and fixed. C. Cut the skin on the inside of the left leg of the mouse and implant the HD-X02 back subcutaneously. D-F. The skin of the right limb of the mouse was cut, an electrocardiogram electrode wrapped with hydrogel was guided subcutaneously to the site, and the muscle was fitted, and the skin was sutured after the auxiliary surgical suture. GH. Attach the other ECG electrode coated with hydrogel to the inner muscle of the left leg. Make sure that the connection between the two electrodes passes through the apex of the heart. I-L. The skin of the right leg of the mouse was cut, a myoelectric electrode wrapped with hydrogel was guided subcutaneously to the site, and the muscle was fitted, and the skin was sutured after the auxiliary surgical suture. M-O. The skin of the left limb of the mouse was cut, another electromyography electrode wrapped with hydrogel was guided subcutaneously to the site, and the muscle was fitted, and the skin was sutured after the auxiliary surgical suture. PQ. After plugging all the electrode wires into the subcutaneous space of the back, the incision of the skin of the left leg was closed, and biological glue was applied to prevent the electrode wires from being exposed to complete the operation.

**Table S1.** Screening of hydrogel formulations to identify compositions with high conductivity, low impedance, and low modulus.

| Strategy | Name | Elastic modulus (kPa) | Conductivity (S/m) | Impedance at 10 Hz (kΩ) |
| --- | --- | --- | --- | --- |
| IPNCH | IPNCH@PAM | 1.36 | 0.29 | 0.92 |
|  | IPNCH@CAPAM | 0.11 | 0.62 | 38.04 |
|  | IPNCH@PAAc | 2.30 | 0.20 | 44646.16 |
|  | IPNCH@PAAc-PVA | 11.86 | 0.87 | 0.14 |
| PSCH | PSCH@IL1 | 17.88 | - | - |
|  | PSCH@IL2 | 68.12 | 0.27 | 1.60 |
|  | PSCH@IL3 | 12.56 | - | - |
|  | PSCH@SBMA | 33.27 | 0.44 | 4.76 |
|  | PSCH@SBAA | 7.75 | 0.47 | 0.23 |
| PCH | PCH@DMSO | 117.00 | 5.20 | 65.77 |
|  | PCH@EG | 15.71 | 4.49 | 22.44 |
|  | PCH@Glycerol | 37.68 | 2.21E-05 | 2.46E07 |
|  | PCH@DMF | 131.00 | 0.19 | 373.22 |
|  | PCH@THF | 23.47 | - | 0.18 |

**Table S2.** Performance comparison of finally selected hydrogel.

|  | Property | IPNCH  @CAPAM | PSCH  @SBMA | PCH@EG |
| --- | --- | --- | --- | --- |
| Mechanical property | Elastic modulus (kPa) | 0.28 | 0.44 | 15.71 |
|  | Tensile strain (%) | 530 | 789 | - |
|  | Tensile stress (kPa) | 8.02 | 5.85 | - |
|  | Compressive strain (%) | 80.45 | 82.31 | 80.63 |
|  | Compressive stress (kPa) | 100.12 | 1161.75 | 1689.11 |
|  | Peel-off strength (N/m) | 17.38 | 8.09 | - |
| Electrical property | Conductivity (S/m) | 1.99 | 5.25 | 3.8 |
|  | Impedance at 10 Hz (kΩ) | 0.09 | 0.04 | 0.02 |
|  | Interfacial resistance (MΩ) | 0.26 | 0.66 | 1.56 |
|  | Initial CSC (μC·cm^-2^) | 12.84 | 11.22 | 56.90 |
|  | ΔCSC for 2000 cycles (%) | 8.13 | 16.38 | 32.63 |
| Viability (%) | | 82.8 | 106 | 105 |

**Table S3.** BET specific surface area analysis of conducting polymer hydrogels.

| Hydrogels | BET specific surface area (m^2^/g) |
| --- | --- |
| IPNCH@CAPAM | 1.181 |
| 5 wt.% PSCH@SBMA | 31.315 |
| 15 wt.% PSCH@SBMA | 8.511 |
| 25 wt.% PSCH@SBMA | 5.735 |
| 35 wt.% PSCH@SBMA | 0.778 |
| 45 wt.% PSCH@SBMA | 0.801 |
| 10 vol.% PCH@EG | 1.371 |
| 15 vol.% PCH@EG | 1.535 |
| 20 vol.% PCH@EG | 4.319 |
| 25 vol.% PCH@EG | 3.590 |
| 30 vol.% PCH@EG | 3.334 |
| 35 vol.% PCH@EG | 2.418 |
| IPNCH@CAPAM after 50 days | 7.038 |
| 25 wt.% PSCH@SBMA after 50 days | 15.044 |
| 25 vol.% PCH@EG after 50 days | 3.680 |

**Table S4.** The average surface roughness of PSCH@SBMA and PCH@EG.

| Hydrogels | The average surface roughness Ra (nm) |
| --- | --- |
| 25 wt.% PSCH@SBMA | 31.7 |
| 35 wt.% PSCH@SBMA | 14.2 |
| 10 vol.% PCH@EG | 5.12 |
| 15 vol.% PCH@EG | 7.14 |
| 20 vol.% PCH@EG | 3.45 |
| 25 vol.% PCH@EG | 12.0 |
| 30 vol.% PCH@EG | 3.62 |
| 35 vol.% PCH@EG | 4.00 |

**Table S5.** Summary of preprocessing steps tailored to signal frequency characteristics and noise sources.

| **Signal Type** | **Major Noise Sources** | **Preprocessing Methods** | **Parameter Rationale** |
| --- | --- | --- | --- |
| EMG | High-frequency noise, motion artifacts | 1. Wavelet denoising (db4, level = 5)  2. High-pass filtering (cutoff optimized iteratively) | EMG signal energy lies in 20–500 Hz; high frequencies must be preserved |
| ECG | Baseline drift, powerline interference | 1. Median filtering (5-point window)  2. High-pass filter (0.5 Hz)  3. Notch filter (50/60 Hz) | ECG components mainly in 0.5–40 Hz; powerline noise is narrowband |
| EEG | Low-frequency drift, ocular artifacts | 1. Artifact removal by dynamic thresholding  2. Bandpass filter (0.4–40 Hz)  3. Notch filter (50/60 Hz) | EEG features (alpha, beta, etc.) typically in 8–30 Hz; full EEG range: 0.4–40 Hz |

**Table S6.** Performance comparison between conducting hydrogels for electrophysiological signal recording.

| **Tensile strain**  **(%)** | **Compressive strain**  **(%)** | **Conductivity (S/m)** | **Elastic modulus (kPa)** | **Tensile/Compressive cycle** | **Cycle strain** | **Stress retention rate** | **Biocompatibility (>80%)** | **Skin adhesion strength (kPa)** | **Cycles and CSC retention rate** | **Cycles and CIC retention rate** | **Epidermal** | **Implantable** | **Ref** |
| --- | --- | --- | --- | --- | --- | --- | --- | --- | --- | --- | --- | --- | --- |
| **530** | **80** | **1.99** | **0.28** | **T 1,000** | **30%** | **83.45%** | **Y** | **11.83** | **10,000 >94%** | **100,000**  **>95%** | **Y** | **Y** | **This work - CAPAM** |
| **789** | **82** | **5.25** | **0.44** | **C 1,000** | **30%** | **79.21%** | **Y** | **5.66 N/m** | **10,000 >83%** | **100,000**  **>87%** | **Y** | **Y** | **This work – SBMA** |
| **-** | **81** | **3.8** | **15.71** | **C 1,000** | **30%** | **83.14%** | **Y** | **N** | **10000,**  **>100%** | **100,000  >100%** | **Y** | **N** | **This work - EG** |
| >10,000 | - | 0.01 | 2000 | T 10 | 500% | 63% | N | N | - | - | Y | N | Nat. Commun. 2019, 10, 1043[1] |
| 1260 | - | N | 45 | N | - | - | Y | 400 J/m^2^ | - | - | N | Y | Adv. Funct. Mater. 2021, 31, 2106446[2] |
| 60 | 60 | 2.47 × 10^−5^ | 1100 | T 1,000 | 15% | 97% | N | 20.78 J /m^2^ on Au | - | - | N | N | Nat. Mater. 2024, 23, 1107–1114[3] |
| 63 | - | 1.44 | 160 | T 250 | 50% | 67.16% | Y | 43 | - | - | Y | Y | Adv. Funct. Mater. 2022, 32, 2208024[4] |
| 200 | - | 2.6 | 293 | N | - | - | Y | 60 | - | 10,000  >95% | Y | Y | Nat. Mater. 2021, 20, 229–236[5] |
| 540 | - | 0.013 | 240 | T 1,000 | 100% | Qualitative | N | 22 kPa  On Glass | - | - | Y | N | Mater. Horiz. 2020,7, 2994-3004[6] |
| 2000 | - | 0.1 | 210 | N | - | - | - | - | - | - | N | N | Nat. Electron. 2019, 2, 75–82[7] |
| 35 | - | 2000 | 2000 | N | - | - | - | - | - | - | N | N | Nat. Commun. 2019, 10, 1043[8] |
| 290 | - | 600 | 7.25 | N | - | - | Y | 78.3 J m–2 for skin | 1,000  >90% | 10,000  >95% | Y | Y | ACS Nano 2023, 17, 2, 885–895[9] |
| 399 | - | 12 | 23 | T 10 | 100% | Qualitative | N | N | - | - | N | N | Nat. Commun. 2018, 9, 2740[10] |
| 390 | 70 | 0.08 | 8 | T 10 | 50% | 96% | Y | 6.5 | - | - | Y | Y | Adv. Funct. Mater. 2023, 33: 2302846.[11] |
| 316 | 80 | 10 | 31.6 | N | - | - | Y | 4 kPa. | - | - | N | Y | Nano Today 2023, 51, 101934.[12] |
| 336 | 80 | 0.125 | 8 | C 70 | 50% | 76.19% | Y | N | - | - | Y | N | ACS Appl. Mater. Interfaces 2022, 14, 45, 51420–51428[13] |
| 95 | - | - | 160 | T 15 | 50% | 71.88% | N | 8.5 | - | - | Y | N | ACS Appl. Polym. Mater. 2021, 3, 10, 5268–5276[14] |
| 280 | - | - | - | N | - | - | N | Qualitative | - | - | Y | N | 2024 IEEE 3rd International Conference on Micro/Nano Sensors for AI, Healthcare, and Robotics (NSENS). IEEE, 2024. p. 86-90[15] |
| 305 | 80 | 2.4 | - | T 500 | 50% | 94% | N | N | - | - | Y | N | Biomater. Sci., 2023,11, 3603-3615[16] |
| 73 | - | - | 5.74 | T 80 | 50% | 85% | Y | N | - | - | Y | N | Gels 2023, 9, 957[17] |
| 105 | - | 0.91 | 170 | T 10 | 80% | 87.9% | N | 7 N/m | - | - | Y | N | COLLOID SURFACE A 2023, 662, 130998[18] |
| 450 | - | 2 | 3 | T 6 | 100% | 82.69% | Y | 13.5 | - | - | Y | N | ACS Appl. Mater. Interfaces 2023, 15, 41, 48744–48753[19] |
| 200 | - | - | 20 | N | - | - | Y | 0.85 N | - | - | Y | N | Sensors 2020, 20, 5737[20] |
| 349 | 80 | 1.35 | 3.43 | T 10 | 80% | 80.77% | N | N | - | - | Y | N | Macromol. Mater. Eng. 2021, 306: 2100341[21] |
| 2400 | - | 1.12 | 4.5 | T 500 | 100% | 50% | Y | 388.1 | - | - | Y | N | ACS Appl. Mater. Interfaces 2023, 15, 9, 11549–11562[22] |
| 82 | 80 | 0.1 | 74.67 | T 3,000 | 80% | Qualitative | Y | 98 | - | - | Y | N | Adv. Mater. Technol. 2024, 9, 2302072[23] |
| 442.3 | 80 | 0.173 | - | T 15;  C 50 | 100%;  15% | Qualitative | N | N | - | - | Y | N | INT J BIOL MACROMOL 2023, 230, 123117[24] |
| 240 | 80 | 0.1074 | 1.1 | N | - | - | Y | 1,000 kPa on glass | - | - | Y | N | COMPOS SCI TECHNOL 2023, 231, 109793[25] |
| 407 | 57 | 5.26 | - | T 10 | 100% | 85% | N | N | - | - | N | N | e-Polymers 2021, 21, 1, 391-397[26] |
| - | - | 2.1 | 16 | N | - | - | N | 3.8 | - | - | N | Y | Small 2023, 19, 2300250[27] |
| 560 | 80 | 1.04 | 4 | T 10 | 100% | 88% | N | 8.01 | - | - | Y | N | J. Mater. Chem. C, 2023,11, 16135-16142[28] |

* N indicates "No";

Y indicates "Yes";

- indicates "Not tested yet"

**Supplementary data processing program**

MATLAB program for preprocessing of electrophysiological signals and calculation of signal-to-noise ratio:

**For EMG processing:**

% Acquisition Configuration

filename = 'EMG.xlsx';

sheet = 'EMG';

% Signal Extraction (Column 2: EMG, Column 1: Timestamps)

emg_signal = emg_data(:, 2);

time_vector = emg_data(:, 1);

% Visualization: Raw Signal

figure;

subplot(4, 1, 1);

plot(time_vector, emg_signal);

title('Raw EMG Signal');

xlabel('Time');

ylabel('Amplitude');

% Wavelet Denoising Configuration

level = 5;% Decomposition levels (trade-off: noise vs detail)

wname = 'db4';% Daubechies4 wavelet - optimal for EMG spikes

denoised_signal = wden(emg_signal, 'rigrsure', 's', 'mln', level, wname);% Rigorous SURE thresholding

% Baseline Correction

baseline_removed_signal = denoised_signal - mean(denoised_signal);

% Downsampling (500Hz -> 250Hz)

downsampled_signal = downsample(baseline_removed_signal, 2);

% SNR Calculation Function (Power Ratio in dB)

calculate_SNR = @(signal, noise) 20 * log10(rms(signal) / std(noise));

% Adaptive Filter Tuning Parameters

cutoff_frequency = 10;

max_iterations = 100;

learning_rate = 0.01;

for i = 1:max_iterations

sampling_frequency = 500;

[b, a] = butter(4, cutoff_frequency / (sampling_frequency / 2), 'high');

filtered_signal = filtfilt(b, a, baseline_removed_signal);

noise = baseline_removed_signal - filtered_signal;

SNR = calculate_SNR(filtered_signal, noise);

% Iterative Filter Optimization

if i > 1

gradient = (SNR - prev_SNR) / (cutoff_frequency - prev_cutoff_frequency);

else

gradient = 0;

end

prev_cutoff_frequency = cutoff_frequency;

prev_SNR = SNR;

cutoff_frequency = cutoff_frequency + learning_rate * gradient;

fprintf('Iteration %d: Cutoff = %.2f Hz, SNR = %.2f dB\n', i, cutoff_frequency, SNR);

end

% 4th-order Butterworth high-pass filter

[b, a] = butter(4, cutoff_frequency / (sampling_frequency / 2), 'high');

% Zero-phase filtering (forward + reverse)

filtered_signal = filtfilt(b, a, baseline_removed_signal);

noise = baseline_removed_signal - filtered_signal;

**For ECG processing:**

% Data Acquisition Configuration

filename = 'ECG.xlsx';

sheet = 'Sheet1';

try

ecg_data = xlsread('XXX.xlsx',1);

catch

error('Failed to read Excel file or specified sheet not exist');

end

% Signal Extraction (Column 2: ECG voltage, Column 1: Timestamps)

ecg_signal = ecg_data(:, 2);

time_vector = ecg_data(:, 1);

% Median Filter for Spike Removal

ecg_signal_denoised = medfilt1(ecg_signal, 5);% 5-sample window removes pacing artifacts

% Baseline Wander Removal (5s moving average)

baseline_window = 5 * 1000;% 5-second window for LF drift (0.2Hz cutoff)

baseline_drift_corrected = ecg_signal_denoised - movmean(ecg_signal_denoised, baseline_window);

% Sliding Mean for Trend Analysis

window_size = 1000;% 1-second window for rhythm analysis

sliding_mean = movmean(baseline_drift_corrected, window_size);

% High-Pass Filtering for Final Cleanup

fs = 1000;% Sampling frequency

fc_low = 0.5;% Cutoff for final baseline removal

[b, a] = butter(4, fc_low/(fs/2), 'high');% 4th-order Butterworth

ecg_signal_filtered = filtfilt(b, a, baseline_drift_corrected);% Zero-phase filtering

% 50Hz Notch Filter for Powerline Interference

notch_freq = 50;

bw = 2;

[b_notch, a_notch] = iirnotch(notch_freq/(fs/2), bw/(fs/2));

ecg_signal_filtered = filtfilt(b_notch, a_notch, ecg_signal_filtered);

% SNR Calculation Protocol

qrs_segment = ecg_signal_filtered(1001:2000);% QRS complex region (signal of interest)

noise_segment = ecg_signal_filtered(1:1000);% TP segment (isoelectric baseline)

SNR = 20*log10(rms(qrs_segment)/rms(noise_segment));% RMS-based SNR

% Spectral Analysis Configuration

N = length(ecg_signal_filtered);

f = (0:N/2-1)*(fs/N);

ecg_fft = fft(ecg_signal_filtered);

ecg_psd = (1/(fs*N)) * abs(ecg_fft(1:N/2)).^2;

**For EEG processing:**

% Data Acquisition Configuration

filename = 'EEG.xlsx';

sheet = 'Sheet1';

try

eeg_data = xlsread('XXX.xlsx',1);

catch

error('Failed to read Excel file or specified sheet not exist');

end

% Signal Extraction (Column 2: EEG voltage, Column 1: Timestamps)

eeg_signal = eeg_data(:, 2);

time_vector = eeg_data(:, 1);

% DC Offset Removal

eeg_signal = eeg_signal - mean(eeg_signal);

% Artifact Rejection (3 MAD threshold)

threshold_upper = median(eeg_signal) + 3*mad(eeg_signal,1); % Median Absolute Deviation

threshold_lower = median(eeg_signal) - 3*mad(eeg_signal,1);

eeg_signal(eeg_signal > threshold_upper | eeg_signal < threshold_lower) = NaN;

eeg_signal = fillmissing(eeg_signal, 'movmedian', 50);% 50-sample window for artifact repair

% Initial Denoising

eeg_signal_denoised = medfilt1(eeg_signal, 5);% 5-point median filter for transient noise

% Bandpass Filtering (Neurophysiological Range)

fs = 1000;% Sampling frequency

fc_low = 0.4;% Lower cutoff (slow waves)

fc_high = 40; % Upper cutoff (gamma waves)

[b, a] = butter(4, [fc_low fc_high] / (fs / 2), 'bandpass');% 4th-order Butterworth

eeg_signal_filtered = filtfilt(b, a, eeg_signal_denoised);% Zero-phase filtering

% Dual Notch Filtering (Global Powerline Standards)

notch_freq_50 = 50;

bw_50 = 2;

[b_notch50, a_notch50] = iirnotch(notch_freq_50/(fs/2), bw_50/(fs/2));

eeg_signal_filtered = filtfilt(b_notch50, a_notch50, eeg_signal_filtered);

notch_freq_60 = 60;

bw_60 = 2;

[b_notch60, a_notch60] = iirnotch(notch_freq_60/(fs/2), bw_60/(fs/2));

eeg_signal_filtered = filtfilt(b_notch60, a_notch60, eeg_signal_filtered);

% Final Detrending

eeg_signal_corrected = detrend(eeg_signal_filtered);% Remove linear trends

% Adaptive Filter Optimization

fc_low_initial = 1;

fc_high_initial = 50;

fc_low_range = [0.5, 1, 2];

fc_high_range = [30, 40, 50];

best_snr = -Inf;

% Grid Search for Optimal Bandstop Parameters

for i = 1:length(fc_low_range)

for j = 1:length(fc_high_range)

[b, a] = butter(4, [fc_low_range(i), fc_high_range(j)] / (fs / 2), 'stop');

eeg_signal_filtered_temp = filtfilt(b, a, eeg_signal_denoised);

% SNR Calculation Protocol

noise_segment = eeg_signal_corrected(1:10*fs);

signal_segment = eeg_signal_corrected(10*fs+1:end);

snr = 20*log10(rms(signal_segment)/rms(noise_segment));

if snr > best_snr

best_snr = snr;

best_fc_low = fc_low_range(i);

best_fc_high = fc_high_range(j);

end

end

end

% Spectral Analysis Preparation

fs = 1000;

win = hamming(length(eeg_signal_corrected));

eeg_signal_windowed = eeg_signal_corrected .* win;

% FFT Configuration

nfft = 2^nextpow2(length(eeg_signal));

f = fs/2*linspace(0,1,nfft/2+1);

eeg_fft = fft(eeg_signal_windowed, nfft);

eeg_psd = abs(eeg_fft(1:nfft/2+1)).^2/(fs*nfft);

alpha_band_power = sum(eeg_psd(f >= alpha_band(1) & f <= alpha_band(2));

other_band_power = sum(eeg_psd(f < alpha_band(1) & f>=1 | f > alpha_band(2) & f < 40));

alpha_to_other_ratio = alpha_band_power / other_band_power;

**Supporting Notes**

1. **Discussion on the basic principle of modification strategy**

The basic principles behind the three strategies proposed in this paper can be summarized as follows:

1. Interpenetrating Polymer Network (IPN) Strategy

In the first strategy, we created an interpenetrating polymer network (IPN) by embedding a conductive polymer, such as PEDOT, into a non-conductive hydrogel matrix. The mechanisms behind this modification include:

1) Hydrogen Bonding and Ionic Interactions: PEDOT's hydrophilic component, PSS, forms hydrogen bonds with hydroxyl or amino groups in the hydrogel, which enhances polymer chain adhesion and reduces interfacial tension. This allows PEDOT to penetrate the hydrogel matrix more effectively, forming a stable cross-linked network.

2) Carrier Mobility and Chain Conjugation: The conjugated structure of PEDOT, where carbon atoms form covalent bonds through sp² hybridization, enhances electron mobility between polymer chains.[10] This structure not only improves conductivity but also strengthens the mechanical properties of the hydrogel through cross-linking.

3) Interface Flexibility: The IPN structure consists of two interpenetrating polymer networks that interact minimally, enabling free movement between them.[29] For example, in the PEDOT:PSS system, weak interactions like hydrogen bonds and van der Waals forces between sulfonic acid groups and polar groups in the hydrogel matrix maintain the material's flexibility without increasing rigidity. The hydrophilic nature of PSS also retains the hydrogel’s softness in high-water-content environments.

In summary, the IPNs strategy not only improves the electrical properties through the intermolecular interaction, but also significantly reduces the modulus of the material and enhances its flexibility and softness through the high water content and dual network structure of the material. This dual nature is the core advantage of IPNs for applications in areas such as biomedicine and wearable electronics.

1. Phase Separation Strategy

In this strategy, ionic compounds are introduced as dopants, triggering phase separation and promoting polymer chain aggregation through several key mechanisms:

1) Reduction of Ion Exchange and Interaction Energy: The anions in ionic compounds form ion pairs with the positively charged PEDOT chains via electrostatic attraction. This ion pairing reduces the overall interaction energy between polymer chains.[30, 31] By mediating electrostatic forces, the ionic compounds mitigate repulsive forces between the chains, allowing the PEDOT chains to draw closer together. This enhanced interaction increases the degree of polymerization, leading to the formation of a denser, more interconnected network structure. The tighter arrangement of polymer chains strengthens interchain attraction, further encouraging the aggregation of PEDOT chains.

2) Enhanced Aggregation and Microstructural Changes: The introduction of ionic compounds amplifies electrostatic interactions between PEDOT chains, facilitating their aggregation. At a molecular level, these dopants raise the ion concentration within the electrolyte, intensifying the mutual attraction between polymer chains. As a result, the PEDOT chains cluster more readily, forming a compact, conductive network. This dense aggregation increases the number of conductive pathways while enhancing electron mobility, which, in turn, boosts both the electrical conductivity and the mechanical integrity of the material.[32]

3) Improved Mechanical Strength and Elastic Modulus: As the interactions between polymer chains grow stronger, the resulting network structure within the hydrogel becomes more robust. This tighter network directly enhances the mechanical strength of the hydrogel. Specifically, the increased interaction between PEDOT chains raises the elastic modulus, as evidenced in Figure 4A. Compared to the interpenetrating polymer network (IPNCH) strategy, this approach results in better toughness and flexibility under mechanical stress, such as tension and compression (Figure 4C, 4E). The ionic compounds also enhance crosslinking between PEDOT chains, reducing the chains' freedom of movement and tightening interchain interactions. This reinforces the structural stability and load-bearing capacity of the hydrogel.

In summary, the incorporation of ionic compounds not only improves the electrical conductivity of the hydrogel but also significantly strengthens its mechanical properties, including elasticity and durability. These modifications highlight the critical role of ionic compounds in optimizing the overall performance of conducting polymer hydrogels for practical applications.

1. Polar Solvent Additives Strategy

In this strategy, the performance of the conducting polymer hydrogel (PCH) is enhanced through the addition of polar solvents (such as DMSO and THF) and subsequent drying and annealing processes. The mechanisms behind these improvements are outlined as follows:

1) Solvent-Polymer Chain Interactions: Polar solvents interact with PEDOT chains by forming hydrogen bonds with hydrogen or oxygen atoms in the polymer. These hydrogen bonds reduce the interaction energy between polymer chains, allowing the solvent molecules to disperse more evenly between them. This increased spacing between chains prevents aggregation and ensures a more uniform dissolution of the PEDOT in the solvent.[33] Additionally, the introduction of polar solvents can alter the conformation of the polymer, increasing the freedom of rotation between chain segments.[34] This greater flexibility allows the polymer to respond more efficiently to external forces, leading to improved mechanical properties such as stretchability and resistance to compression.

2) Chain Reordering During the dry annealing process, thermal excitation increases the mobility of the polymer chains.[35-37] As the temperature rises, molecular motion promotes the reorganization of the chains, allowing the PEDOT chains to adopt a more orderly arrangement in a molten state. This ordered structure forms a stable polymer network, enhancing the material's ability to transfer external stresses. The improved chain alignment directly impacts the polymer’s mechanical resilience, enabling it to better absorb and distribute mechanical loads.

3) Hydration-Induced Network Optimization: Hydration further optimizes the polymer network by affecting interchain interactions. Water molecules, acting as polar solvents, strengthen the connections between polymer chains through hydrogen bonding and electrostatic interactions. This enhanced bonding increases the flexibility and elasticity of the hydrogel. Hydration can also promote additional cross-linking between polymer chains, resulting in a more stable network. This increased cross-linking improves the elastic modulus and tensile strength of the material, enabling it to withstand greater external stresses without failure.

In summary, the combined effects of polar solvent addition, thermal annealing, and hydration significantly improve the mechanical and electrical properties of conducting polymer hydrogels. These modifications allow the hydrogel to meet the demands of various application scenarios, offering enhanced performance and durability.

The common goal of these strategies is to optimize the microstructure and phase behavior of conductive polymers within hydrogel matrices to enhance mechanical and electrical properties.

1. **Test and analysis of yield of three hydrogels.**

After freeze-drying the hydrogels to remove all moisture and solvents, we measured the weight of the dried hydrogel to represent the actual product yield. The yield was calculated using the following formula:

$$\begin{aligned} Yield=\frac{Mass of dried hydrogel}{Mass of solid components in raw material}\times100\%\#\left( S6 \right) \end{aligned}$$

According to the above experiments and calculations, the yield of the three strategies were:

|  | Yield (%) |
| --- | --- |
| IPNCH@CAPAM | 95.22 |
| PSCH@SBMA | 89.84 |
| PCH@EG | 85.48 |

These values reflect the material utilization rate during hydrogel synthesis, specifically the conversion efficiency of raw materials into the final dried product.

The high yield of IPNCH@CAPAM (95.22%) is largely attributed to its stable and efficient preparation process. The carrageenan (CA) matrix forms a robust gel under alkaline conditions, minimizing material loss. Dopamine (DA) polymerizes to polydopamine in this environment, which enhances hydrogel crosslinking. PEDOT:PSS, added as a freeze-dried powder, disperses uniformly, reducing waste. Acrylamide (AM) and the crosslinker BIS undergo efficient polymerization, with minimal loss of unreacted monomers. Moreover, the reaction occurs at room temperature, with a moderate exothermic rise that peaks below 75°C, avoiding excessive decomposition or volatilization.

In contrast, the yield for the PSCH@SBMA system (89.84%) is slightly lower. This reduction is primarily due to the higher reaction temperature (90°C), which causes some volatilization and degradation of organic components. The PEDOT dispersion process can also be suboptimal due to its complex structure and high viscosity, leading to aggregation. SBMA, an ionic compound with strong polarity, improves conductivity but may also volatilize or undergo side reactions at elevated temperatures, resulting in some material loss.

The PCH@EG system exhibited the lowest yield (85.48%), likely due to the decomposition of PEDOT and volatilization of ethylene glycol during high-temperature annealing. Ethylene glycol, a polar solvent, is particularly prone to evaporation at temperatures of 60°C (during drying) and 130°C (during annealing), reducing the overall solid content. Although high-temperature annealing improves the conductivity of PEDOT, repeated cycles can cause material degradation, further impacting the yield.

The variation in yields across these strategies is closely tied to material reactivity, the gentleness of the preparation process, and the impact of high-temperature treatments. A higher yield, as observed in IPNCH@CAPAM, suggests a more efficient material conversion process and a more straightforward, scalable synthesis method. This makes it suitable for large-scale applications, particularly in areas requiring stable performance, such as electrophysiological signal recording. Conversely, the lower yield and more complex preparation of PCH@EG limit its feasibility for mass production. However, its unique electrical properties may still make it a strong candidate for specialized high-performance electronic applications where conductivity is critical. Additionally, yield discrepancies can affect the amount of waste produced, raising environmental concerns. As a result, further attention is needed to optimize these processes, not only to improve yield but also to address sustainability.

1. **The measurement and analysis of crosslink efficiency of three different strategies.**

1) IPNCH@CAPAM system

In this system, the conductive polymer serves as a dopant to establish an interpenetrating polymer network (IPN). Carrageenan (CA) contributes a robust hydrating hydrogen bond network, while PEDOT enhances electrical conductivity. The characteristic of the IPN—where multiple networks intersperse—results in a relatively low crosslinking density of 0.11 mol/cm³. This lower density correlates with a higher swelling ratio, indicating that fewer chemical crosslinking points facilitate the ingress of water molecules into the network, leading to considerable volume expansion. The gel fraction of 65.75% further supports this observation, suggesting that the system relies heavily on physical rather than chemical crosslinking, thus reducing overall crosslinking efficiency.

Despite its lower crosslinking density, the combination of PEDOT and acrylamide (AM) within this structure allows for commendable electrical conductivity. However, this may compromise the elastic modulus, resulting in a potentially weaker mechanical profile. The advantageous aspect of the IPN configuration is its ability to balance flexibility and conductivity without excessive crosslinking, allowing the hydrogel to adapt to tissue deformation and minimize mechanical stress on surrounding biological structures. Therefore, this hydrogel is particularly suitable for applications requiring significant deformation or flexibility, such as long-term implanted electrophysiological signal recording devices. Its reduced stiffness fosters better compatibility with cell movement in dynamic environments, such as cardiac or muscular tissues, mitigating biological incompatibility concerns associated with rigid materials.

2) PSCH@SBMA system

The incorporation of the ionic compound SBMA into the PSCH@SBMA system facilitates the aggregation of PEDOT and significantly enhances conductivity. The ionic groups in SBMA engage in ionic interactions with PEDOT, resulting in a denser internal structure and a slight increase in crosslinking density to 0.18 mol/cm³ compared to IPNCH@CAPAM. The swelling ratio of 2388.71% indicates that while the system retains hydration capacity, it is markedly lower than that of the IPNCH@CAPAM system. This reduction is attributable to SBMA's role in augmenting crosslinking efficiency through ionic interactions, thereby restricting water molecule infiltration.

Additionally, the gel fraction of 71.80% aligns with the increased crosslinking density, illustrating that the incorporation of ionic compounds enhances the hydrogel's crosslinking efficiency. The heightened aggregation and physical crosslinking brought about by SBMA lead to improved electrical conductivity and mechanical properties, including a higher elastic modulus. However, the presence of strong ionic interactions may contribute to increased rigidity, thus diminishing swelling behavior. The synergistic effect of conductive and ionic components bestows the PSCH@SBMA hydrogel with excellent conductive properties and mechanical strength, making it apt for electrophysiological signal sensing and recording, especially in short- to medium-term implant scenarios. This hydrogel's balanced properties ensure stable mechanical strength and effective signal transmission, making it suitable for applications such as electroencephalogram (EEG) and electrocardiogram (ECG) sensors.

3) PCH@EG system

In the PCH@EG system, the addition of ethylene glycol (EG) as a polar solvent significantly promotes the rearrangement of PEDOT molecular chains, leading to a compact polymer network. This results in a remarkably high crosslinking density of 6.34 mol/cm³, surpassing that of both the IPNCH@CAPAM and PSCH@SBMA systems. The increased chemical crosslinking points within the system, attributed to the rearrangement in the polar solvent, restrict water molecule infiltration, resulting in a relatively low swelling ratio of 1499.10%. Correspondingly, the gel fraction of 72.59% indicates a high crosslinking efficiency, reflecting the retention of most materials in the gel structure through chemical crosslinking or physical interactions.

The high crosslinking density and low swelling ratio in the PCH@EG system are indicative of the tight aggregation of PEDOT molecular chains facilitated by annealing and solvent interactions. This configuration grants the system superior mechanical properties and enhanced electrical conductivity, positioning it as a robust candidate for applications requiring high stability. However, the increased rigidity associated with this dense structure may compromise flexibility and limit water molecule infiltration, resulting in reduced swelling capabilities. As a result, this hydrogel is more suited for environments demanding high mechanical stability, such as electrophysiological signal recording devices that operate under strict external conditions. Its impressive mechanical strength ensures that the hydrogel maintains its shape and integrity over extended use, making it ideal for applications such as fixed sensing devices, short-term surgical aids, or any scenario that does not require significant deformation.

1. **The consistency of polymerization reactions across different non-conductive polymer templates in conductive hydrogels and their impact on mechanical and electrical properties.**

1) Consistency of Polymerization

In our experiments involving the interpenetrating polymer network of conductive hydrogels (IPNCH), we maintained consistent polymerization conditions across all non-conductive templates. This included using the same type and concentration of crosslinkers, initiators, and conductive polymers. Specifically, for the polyvinyl alcohol (PVA) template, which undergoes crosslinking via hydroxyl groups rather than traditional radical polymerization, we introduced acrylic copolymerization to establish a three-dimensional cross-linked network. Overall, we ensured that the total concentration of monomers in the IPNCH was uniform. These standardized reaction conditions allow for meaningful comparisons between hydrogels formed with different non-conductive polymer templates.

2) Influence of Non-Conductive Polymer Template Type

While the reaction conditions were identical, variations in the mechanical and electrical properties of the resulting systems primarily stem from the inherent chemical structures and physical crosslinking characteristics of the templates used. For example, the high molecular weight and robust physical crosslinking capability of PVA enable it to create a stable network structure, thereby enhancing both the mechanical properties and electrical conductivity of the hydrogels. In contrast, carrageenan (CAPAM) yields lower elastic modulus and conductivity due to its softer structure.

The carboxyl groups in polyacrylamide (PAM) and polyacrylic acid (PAAc) form physical crosslinks through hydrogen bonding, whereas PVA relies on a combination of hydrogen bonds and van der Waals forces, which further stabilizes its network structure. CAPAM's lower degree of physical crosslinking results in diminished mechanical properties and conductivity. Additionally, the hydrophilicity of the polymers impacts hydrogel conductivity. PAM's excellent hydration capability facilitates uniform dispersion of PEDOT, enhancing conductivity, while the carboxyl group in PAAc exerts a less significant effect on hydration.

Moreover, the differences in non-conductive templates affect their interactions with conductive polymers, significantly influencing the arrangement of polymer chains, network structure, and resulting properties. As a linear polymer, PAM exhibits long chain lengths and good fluidity during polymerization, promoting a relatively uniform network structure; however, this may compromise the final mechanical strength. CAPAM enhances mechanical properties and toughness by increasing chain interactions, resulting in complex crosslinked structures. The electronegativity of PAAc introduces repulsion in the polymer chain arrangement, promoting a tighter network structure and improving interactions with PEDOT, thus enhancing electrical conductivity.

In the PAAc-PVA copolymerization system, PVA contributes to improved water resistance and mechanical properties, leading to a more stable network structure. Differences in polymerization reactions and crosslinking processes among various template systems directly influence the network structures, which in turn affect the mechanical and electrical properties of the hydrogels.

3) Influence of Non-Conductive Polymer Chain Length

The length of polymer chains significantly impacts the flexibility and ductility of hydrogels. Longer polymer chains enhance the flexibility of the hydrogels, reduce brittleness, and improve compatibility with biological tissues. For instance, the extended chain structures of polyacrylamide (PAM) and polyacrylic acid (PAAc) create more crosslinking points, thereby enhancing the mechanical properties of the hydrogels.

The conductivity of conductive hydrogels is influenced not only by the distribution of PEDOT but also by the polymer chain length. Longer chains increase the network crosslinking density, resulting in a more uniform dispersion of PEDOT throughout the hydrogels, which, in turn, enhances overall conductivity. Conversely, shorter chains can disrupt the conductive pathways, negatively affecting the stability of the hydrogel's conductive properties.

Although the polymerization conditions are consistent across different templates, variations in polymer chain length arise from the unique chemical structures and physical properties of these non-conductive templates. These differences manifest in the final mechanical and electrical properties of the hydrogels.

4) Differential Analysis of Mechanical Properties

The experimental results (Figure 2B) demonstrate that different non-conductive polymer templates significantly influence the mechanical properties of conductive hydrogels. Variations in mechanical properties, such as elastic modulus (G'), primarily stem from the chemical structure and physical crosslinking characteristics of the polymers. The specific data are as follows:

PAM-based conductive hydrogel: elastic modulus of approximately 1.4 kPa;

CAPAM-based conductive hydrogel: the lowest elastic modulus at around 0.1 kPa;

PAAc-based conductive hydrogel: elastic modulus of about 2.3 kPa;

PAAc-PVA-based conductive hydrogel: the highest elastic modulus at approximately 11.9 kPa.

Among these, IPNCH@CAPAM, with the lowest elastic modulus, exhibits ultra-soft mechanical properties. This low modulus is primarily attributed to the inhibition of PAM chain growth caused by catechol groups in dopamine (PDA), leading to shortened chain lengths and reduced entanglement. Additionally, the sulfate group in CA (−SO₄²⁻) forms weaker physical crosslinks with the amide groups in PAM, further decreasing the rigidity of the system. This ultra-soft characteristic closely aligns with the mechanical properties of brain tissue, making it suitable for applications such as brain implants in biomedicine.

In contrast, the PAAc-PVA copolymerization system demonstrates the highest elastic modulus, which can be linked to the strong physical crosslinking provided by PVA. The robust network formed by hydrogen bonding in PVA facilitates the uniform dispersion and embedding of PEDOT, significantly enhancing the overall mechanical strength. The mechanical properties of PAM and PAAc-based conductive hydrogels fall between those of CAPAM and PVA. As a linear polymer, PAM features a loose crosslinked network and high water content, which contribute to its flexibility, although its elastic modulus is lower than that of PAAc-based hydrogels. The introduction of carboxyl groups in PAAc creates a certain physical crosslinking structure, thereby enhancing its mechanical properties.

5) Analysis of Electrical Performance

In all systems, the conductive polymer serves as the primary source of conductivity for the hydrogels. We standardized the initial addition of conductive polymer to 0.06 wt.% across all systems. The conductivity data (Figure 2C) indicate that while different non-conductive polymer templates yield varying conductivity levels, they generally meet the requirements for bioelectronic device applications. The specific conductivity values are as follows:

PAM-based conductive hydrogel: 0.3 S/m;

CAPAM-based conductive hydrogel: 0.6 S/m;

PAAc-based conductive hydrogel: 0.2 S/m;

PAAc-PVA-based conductive hydrogel: 0.9 S/m.

The conjugated π-electron structure of conducting polymers, such as PEDOT:PSS, contributes to their exceptional electrical conductivity. In all systems, the dispersion and crosslinking of PEDOT directly influence the conductivity of the hydrogel. The sulfonic acid group in PSS enhances its dispersion in aqueous solutions, thereby improving the overall conductivity of the hydrogels.

In the PAAc-PVA system, the relatively tight PVA crosslinking network allows for an even distribution of PEDOT within the hydrogel, resulting in the highest conductivity. PAM, with its high water content and softness, maintains a relatively good electrical conductivity. Although the carboxyl group in PAAc can interact with PEDOT, its weaker ionic bond somewhat limits conductivity. Despite having lower conductivity than the PAAc-PVA system, CAPAM remains suitable for long-term electrophysiological signal transmission with biological tissues due to its ultra-soft mechanical properties and good biocompatibility.

In summary, our study clarifies the consistency of polymerization reactions across different non-conductive polymer templates in conductive hydrogels and their impact on mechanical and electrical properties. These differences arise primarily from the chemical structure and crosslinking mechanisms of the templates, reflecting the combined effects of polymer type and chain length. The PAAc-PVA-based system exhibits the highest mechanical modulus and conductivity, while the CAPAM system demonstrates the lowest modulus due to the polymerization-inhibiting effects of PDA, making it particularly suitable for applications that require soft tissue matching. Overall, the electrical conductivity of all systems is appropriate for electrophysiological signal detection and bioelectronic device applications.

**Reference**

[1] Z. Lei, P. Wu, A highly transparent and ultra-stretchable conductor with stable conductivity during large deformation, Nature Communications 10(1) (2019) 3429.

[2] Y. Xue, J. Zhang, X. Chen, J. Zhang, G. Chen, K. Zhang, J. Lin, C. Guo, J. Liu, Trigger-Detachable Hydrogel Adhesives for Bioelectronic Interfaces, Advanced Functional Materials 31(47) (2021) 2106446.

[3] Y. He, Y. Cheng, C. Yang, C.F. Guo, Creep-free polyelectrolyte elastomer for drift-free iontronic sensing, Nature Materials 23(8) (2024) 1107-1114.

[4] X. Xia, Q. Liang, X. Sun, D. Yu, X. Huang, S.M. Mugo, W. Chen, D. Wang, Q. Zhang, Intrinsically Electron Conductive, Antibacterial, and Anti-swelling Hydrogels as Implantable Sensors for Bioelectronics, Advanced Functional Materials 32(48) (2022) 2208024.

[5] J. Deng, H. Yuk, J. Wu, C.E. Varela, X. Chen, E.T. Roche, C.F. Guo, X. Zhao, Electrical bioadhesive interface for bioelectronics, Nature Materials 20(2) (2021) 229-236.

[6] X. Qu, W. Niu, R. Wang, Z. Li, Y. Guo, X. Liu, J. Sun, Solid-state and liquid-free elastomeric ionic conductors with autonomous self-healing ability, Materials Horizons 7(11) (2020) 2994-3004.

[7] Y. Cao, Y.J. Tan, S. Li, W.W. Lee, H. Guo, Y. Cai, C. Wang, B.C.K. Tee, Self-healing electronic skins for aquatic environments, Nature Electronics 2(2) (2019) 75-82.

[8] B. Lu, H. Yuk, S. Lin, N. Jian, K. Qu, J. Xu, X. Zhao, Pure PEDOT:PSS hydrogels, Nature Communications 10(1) (2019) 1043.

[9] M. Yang, P. Chen, X. Qu, F. Zhang, S. Ning, L. Ma, K. Yang, Y. Su, J. Zang, W. Jiang, T. Yu, X. Dong, Z. Luo, Robust Neural Interfaces with Photopatternable, Bioadhesive, and Highly Conductive Hydrogels for Stable Chronic Neuromodulation, ACS Nano 17(2) (2023) 885-895.

[10] V.R. Feig, H. Tran, M. Lee, Z. Bao, Mechanically tunable conductive interpenetrating network hydrogels that mimic the elastic moduli of biological tissue, Nature Communications 9(1) (2018) 2740.

[11] X. Huang, C. Chen, X. Ma, T. Zhu, W. Ma, Q. Jin, R. Du, Y. Cai, M. Zhang, D. Kong, M. Wang, J.a. Ren, Q. Zhang, X. Jia, In Situ Forming Dual-Conductive Hydrogels Enable Conformal, Self-Adhesive and Antibacterial Epidermal Electrodes, Advanced Functional Materials 33(38) (2023) 2302846.

[12] L. Yan, C. Zhao, Y. Wang, Q. Qin, Z. Liu, Y. Hu, Z. Xu, K. Wang, X. Jiang, L. Han, X. Lu, Adhesive and conductive hydrogel-based therapy simultaneously targeting neuroinflammation and neurofunctional damage after brain injury, Nano Today 51 (2023) 101934.

[13] Y. Gao, Y. Gao, Z. Zhang, F. Jia, G. Gao, Acetylated Distarch Phosphate-Mediated Tough and Conductive Hydrogel for Antibacterial Wearable Sensors, ACS Applied Materials & Interfaces 14(45) (2022) 51420-51428.

[14] D. Wang, L. Qin, W. Yang, Y. He, S. Zhang, Y. Yang, K. Xu, P. Gao, J. Yu, K. Cai, A Conductive Hydrogel Based on GaIn and PVA/PAA/Fe3+ for Strain Sensor and Physiological Signal Detection, ACS Applied Polymer Materials 3(10) (2021) 5268-5276.

[15] G. Zhao, Q. Zhang, Z. Li, J. Wang, G. Guo, Z. Zhao, X. Yu, High Performance Conductive Composite Hydrogel Interface for Epidermal Electrophysiological Monitoring, 2024 IEEE 3rd International Conference on Micro/Nano Sensors for AI, Healthcare, and Robotics (NSENS), 2024, pp. 86-90.

[16] Q. Pang, K. Wu, Z. Jiang, F. Yang, Z. Shi, H. Gao, C. Zhang, R. Hou, Y. Zhu, Nanostructured ionic hydrogel with integrated conductivity, stretchability and thermal responsiveness for a high-performance strain and temperature sensor, Biomaterials Science 11(10) (2023) 3603-3615.

[17] D. Lee, J. Song, J. Kim, J. Lee, D. Son, M. Shin, Soft and Conductive Polyethylene Glycol Hydrogel Electrodes for Electrocardiogram Monitoring, Gels 9(12) (2023) 957.

[18] Y. Shi, X. Fu, W. Wang, D. Yu, Stretchable, adhesive and low impedance hydrogel prepared by one-pot method used as ECG electrodes, Colloids and Surfaces A: Physicochemical and Engineering Aspects 662 (2023) 130998.

[19] Y. Bai, S. Yan, Y. Wang, Q. Wang, X. Duan, Facile Preparation of a Self-Adhesive Conductive Hydrogel with Long-Term Usability, ACS Applied Materials & Interfaces 15(41) (2023) 48744-48753.

[20] Y. Lee, S.-G. Yim, G.W. Lee, S. Kim, H.S. Kim, D.Y. Hwang, B.-S. An, J.H. Lee, S. Seo, S.Y. Yang, Self-Adherent Biodegradable Gelatin-Based Hydrogel Electrodes for Electrocardiography Monitoring, Sensors 20(20) (2020) 5737.

[21] K. Chen, Y. Hu, M. Liu, F. Wang, P. Liu, Y. Yu, Q. Feng, X. Xiao, Highly Stretchable, Tough, and Conductive Ag@Cu Nanocomposite Hydrogels for Flexible Wearable Sensors and Bionic Electronic Skins, Macromolecular Materials and Engineering 306(10) (2021) 2100341.

[22] H. Huang, J. Shen, S. Wan, L. Han, G. Dou, L. Sun, Wet-Adhesive Multifunctional Hydrogel with Anti-swelling and a Skin-Seamless Interface for Underwater Electrophysiological Monitoring and Communication, ACS Applied Materials & Interfaces 15(9) (2023) 11549-11562.

[23] X. Guo, W. Qin, C. Gu, X. Li, M. Chen, H. Zhai, X. Zhao, H. Liu, B. Zhao, Y. Zhang, Y. Wang, S. Yin, High-Adhesion, Weather Resistance, Reusable PAM/Gly/Gel/TA/Fe3+ Biopolymer Dual-Network Conductive Hydrogel for Flexible Bioelectrode, Advanced Materials Technologies 9(9) (2024) 2302072.

[24] Z. Li, F. Yin, W. He, T. Hang, Z. Li, J. Zheng, X. Li, S. Jiang, Y. Chen, Anti-freezing, recoverable and transparent conductive hydrogels co-reinforced by ethylene glycol as flexible sensors for human motion monitoring, International Journal of Biological Macromolecules 230 (2023) 123117.

[25] J. Wei, X. Zhang, F. Wang, Y. Shao, W.-B. Zhang, H. Wu, One-step preparation of highly viscoelastic, stretchable, antibacterial, biocompatible, wearable, conductive composite hydrogel with extensive adhesion, Composites Science and Technology 231 (2023) 109793.

[26] T. Liu, R. Zhang, J. Liu, L. Zhao, Y. Yu, High strength and conductive hydrogel with fully interpenetrated structure from alginate and acrylamide, e-Polymers 21(1) (2021) 391-397.

[27] J. Park, S. Lee, M. Lee, H.-S. Kim, J.Y. Lee, Injectable Conductive Hydrogels with Tunable Degradability as Novel Implantable Bioelectrodes, Small 19(21) (2023) 2300250.

[28] Y. Zhao, Q. Zhao, S. Peng, H. Zhou, L. Yang, A robust conductive organohydrogel with adhesive and low-hysteresis properties for all-weather human motion and wireless electrocardiogram sensing, Journal of Materials Chemistry C 11(46) (2023) 16135-16142.

[29] J. Chen, Q. Peng, T. Thundat, H. Zeng, Stretchable, Injectable, and Self-Healing Conductive Hydrogel Enabled by Multiple Hydrogen Bonding toward Wearable Electronics, Chemistry of Materials 31(12) (2019) 4553-4563.

[30] D. Liu, Y. Cao, P. Jiang, Y. Wang, Y. Lu, Z. Ji, X. Wang, W. Liu, Tough, Transparent, and Slippery PVA Hydrogel Led by Syneresis, Small 19(14) (2023) 2206819.

[31] E. Marwanta, T. Mizumo, H. Ohno, Improved ionic conductivity of nitrile rubber/Li(CF3SO2)2N composites by adding imidazolium-type zwitterion, Solid State Ionics 178(3) (2007) 227-232.

[32] R.L. Manzanares, T.-H. Huang, O. Setiawan, H.F.M. Austria, H.-C. Chang, L.L. Tayo, W.-S. Hung, C.C. Hu, K.-R. Lee, J.-Y. Lai, Investigation of the effects of different zwitterions in fabricating smart PVDF/graphene pervaporation membranes, Separation and Purification Technology 335 (2024) 126133.

[33] Y. Li, X. Deng, L. Zheng, X. Jiao, X. Zhao, Preparation of Conductive Hydrogels by Using the Triple Helix Structure of Gels, ACS Applied Polymer Materials 6(1) (2024) 746-755.

[34] G.L. Mario Cheong, K.S. Lim, A. Jakubowicz, P.J. Martens, L.A. Poole-Warren, R.A. Green, Conductive hydrogels with tailored bioactivity for implantable electrode coatings, Acta Biomaterialia 10(3) (2014) 1216-1226.

[35] B. Zhang, G. Fu, Y. Li, L. Liang, N.S. Grundish, Y. Tang, J.B. Goodenough, Z. Cui, General Strategy for Synthesis of Ordered Pt3M Intermetallics with Ultrasmall Particle Size, Angewandte Chemie International Edition 59(20) (2020) 7857-7863.

[36] B. Chen, Q. Chen, S. Xiao, J. Feng, X. Zhang, T. Wang, Giant negative thermopower of ionic hydrogel by synergistic coordination and hydration interactions, Science Advances 7(48) (2021) eabi7233.

[37] Y. Zhai, X. Meng, H. Duan, Z. Ding, Y. Liu, L. Lucia, Super Stable and Tough Hydrogel Containing Covalent, Crystalline, and Ionic Cross-Links, Macromolecular Chemistry and Physics 217(1) (2016) 32-38.
